# Microwave-assisted C–C bond formation of diarylacetylenes and aromatic hydrocarbons on carbon beads under continuous-flow conditions

**DOI:** 10.1038/s42004-023-00880-y

**Published:** 2023-04-24

**Authors:** Tsuyoshi Yamada, Wataru Teranishi, Naoya Sakurada, Seiya Ootori, Yuka Abe, Tomohiro Matsuo, Yasuharu Morii, Masatoshi Yoshimura, Takeo Yoshimura, Takashi Ikawa, Hironao Sajiki

**Affiliations:** 1 Laboratory of Organic Chemistry, Gifu Pharmaceutical University 1-25-4 Daigaku-Nishi, Gifu, 501-1196 Gifu, Japan; 2 Product Division, Tokyo Rikakikai Co., Ltd. (Brand: EYELA), 1-15-17 Koishikawa, Bunkyo-Ku, 112-0002 Tokyo Japan; 3 R&D Center, N.E. Chemcat Corporation, 678 Ipponnmatsu, Numazu, 410-0314 Shizuoka Japan; 4 SAIDA FDS INC., 143-10 Isshiki, Yaizu, 425-0054 Shizuoka Japan

**Keywords:** Heterogeneous catalysis, Sustainability, Microwave chemistry, Flow chemistry

## Abstract

The synthesis of polycyclic aromatic compounds generally requires stoichiometric oxidants or homogeneous metal catalysts, however, the risk of contamination of inorganic residues can affect their properties. Here we present a microwave (MW)-assisted platinum on beaded activated carbon (Pt/CB)-catalyzed C–C bond formation of diarylacetylenes and aromatic hydrocarbons under continuous-flow conditions. Various fused aromatic compounds were continuously synthesized via dehydrogenative C(sp^2^)–C(sp^2^) and C(sp^2^)–C(sp^3^) bond formation with yields of up to 87% without the use of oxidants and bases. An activated, local reaction site on Pt/CB in the flow reaction channel reaching temperatures of more than three hundred degrees Celsius was generated in the catalyst cartridge by selective microwave absorption in CB with an absorption efficiency of > 90%. Mechanistic experiments of the transformation reaction indicated that a constant hydrogen gas supply was essential for activating Pt. This is an ideal reaction with minimal input energy and no waste production.

## Abstract (web summary)

The synthesis of polycyclic aromatic compounds generally requires stoichiometric oxidants or homogeneous metal catalysts, however, the risk of contamination of inorganic residues can affect their properties. Here, the authors develop a microwave-assisted platinum on beaded activated carbon-catalyzed C–C bond formation of aromatic compounds under continuous-flow conditions.

## Introduction

Recently, π-conjugated polyaromatic compounds, including nanographenes and polycyclic aromatic hydrocarbons (PAHs), have attracted much attention owing to their attractive properties^1–4^. π-Aromatics have been utilized as organic electronic materials, such as organic field-effect transistors (OFET)^5,6^, organic solar cells (OSC)^7,8^, and organic light-emitting diodes (OLED)^9–15^, owing to their unique conjugation, conductivity, redox activity, and luminescent properties^16^. Moreover, the development of sensors for the microanalysis of explosive, high-energy aromatic nitro compounds (picric acid, TNT, etc.)^17^ is expected to utilize the quenching effect of luminescent π-aromatics based on π–π interactions.

**Fig. 1:**
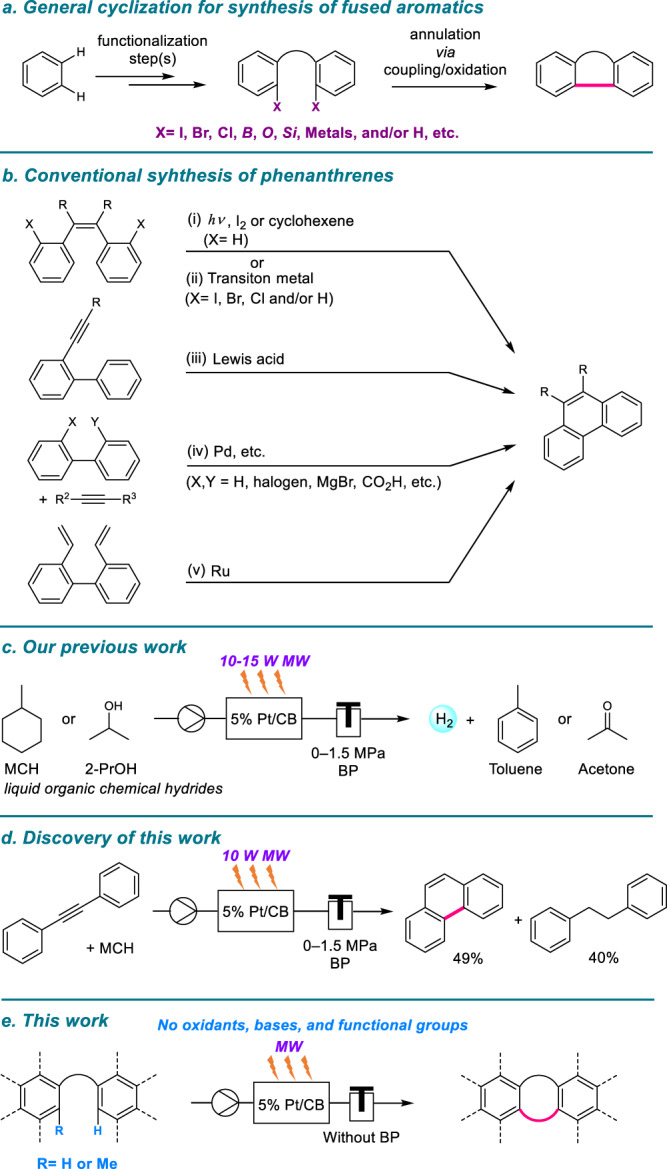
Overview of aromatic C–C bond formations and origins of this study. **a**, **b** Conventional strategy for the synthesis of fused aromatic hydrocarbons. **c** Pt/CB-catalyzed dehydrogenation reaction of liquid organic hydrates. **d**, **e** MW-assisted Pt/CB-catalyzed cyclization reactions (this work).

Polycyclic aromatic compounds are generally synthesized by various functionalization processes, such as oxidation, coupling, and/or cycloaromatization of alkanes and benzene, toluene, and xylenes (BTXs), derived from the catalytic reforming^18,19^ of petroleum (Fig. 1a)^20^. For example, several conventional methods for synthesizing phenanthrene derivatives, one of the smallest polycyclic aromatic hydrocarbons, have been developed (Fig. 1b). Photochemical-cyclization-oxidation reactions of diaryl ethylene derivatives under ultraviolet (UV) irradiation (i)^21^, called the Mallory reaction^22^ and transition-metal-catalyzed intramolecular cyclization reactions of styrene derivatives based on C–H/C–X (X = halogen) activation (ii)^23,24^ are well-developed methods for the synthesis of phenanthrene cores. Phenanthrene structures are also synthesized from biphenyls; for example, the acid-catalyzed intramolecular cyclization of 2-alkynyl biphenyl (iii)^25,26^, cross-coupling reactions of 2-halogenated biphenyl with alkynes (iv)^27–29^, and ring-closing metathesis of 2,2’-vinyl biphenyls (v)^30,31^. Itami et al. have developed various sophisticated molecular transformation methodologies for the synthesis of different π-aromatics, including phenanthrene derivatives^32^, based on transition-metal-catalyzed coupling and subsequent annulation reactions^33,34^. However, previously reported synthetic methodologies require stoichiometric amounts of oxidants and/or homogeneous metal catalysts; therefore, the risk of contamination of inorganic residues derived from catalysts and/or substrates into products is an issue that should be addressed to achieve super-high-purity organic electronic materials with excellent performance^35–37^. Alternatively, the use of heterogeneous metal catalysts could reduce metal contamination^38^ and the complicated workup process after the reaction^39^. Therefore, innovative and practical synthetic methodologies for π-aromatics based on heterogeneous catalysis are highly desirable.

Microwave (MW)-assisted organic synthesis (MAOS) has gained popularity in academic and industrial research because it can selectively and rapidly heat reactants^40–42^. The rapid and selective heating effects of MW often reduce the reaction time from an hour to minutes or even seconds, reducing the total reaction cost compared with conventional external heating methods^41^. However, despite the attractive advantages of MAOS, non-negligible side reactions often occur owing to the superabundant energy supply to the reaction materials (substrates, solvents, or products). For example, microwave flash pyrolysis (MFP)^43,44^, based on flash-flow pyrolysis (FFP)^45^ or flash-vacuum pyrolysis (FVP)^46^, is a known technique for instantaneously promoting various molecular transformations under high thermal conditions (typically 400–1100 °C). These methodologies can produce attractive intermediates and products by repeated cleavage reactions and the formation of various stable bonds. However, their application in organic synthesis is limited, and their selectivity and yields are often ignored in the case of functional materials^43,44,47^. Therefore, selective and well-controlled non-overheating irradiation of MW is essential to achieve efficient syntheses.

**Fig. 2:**
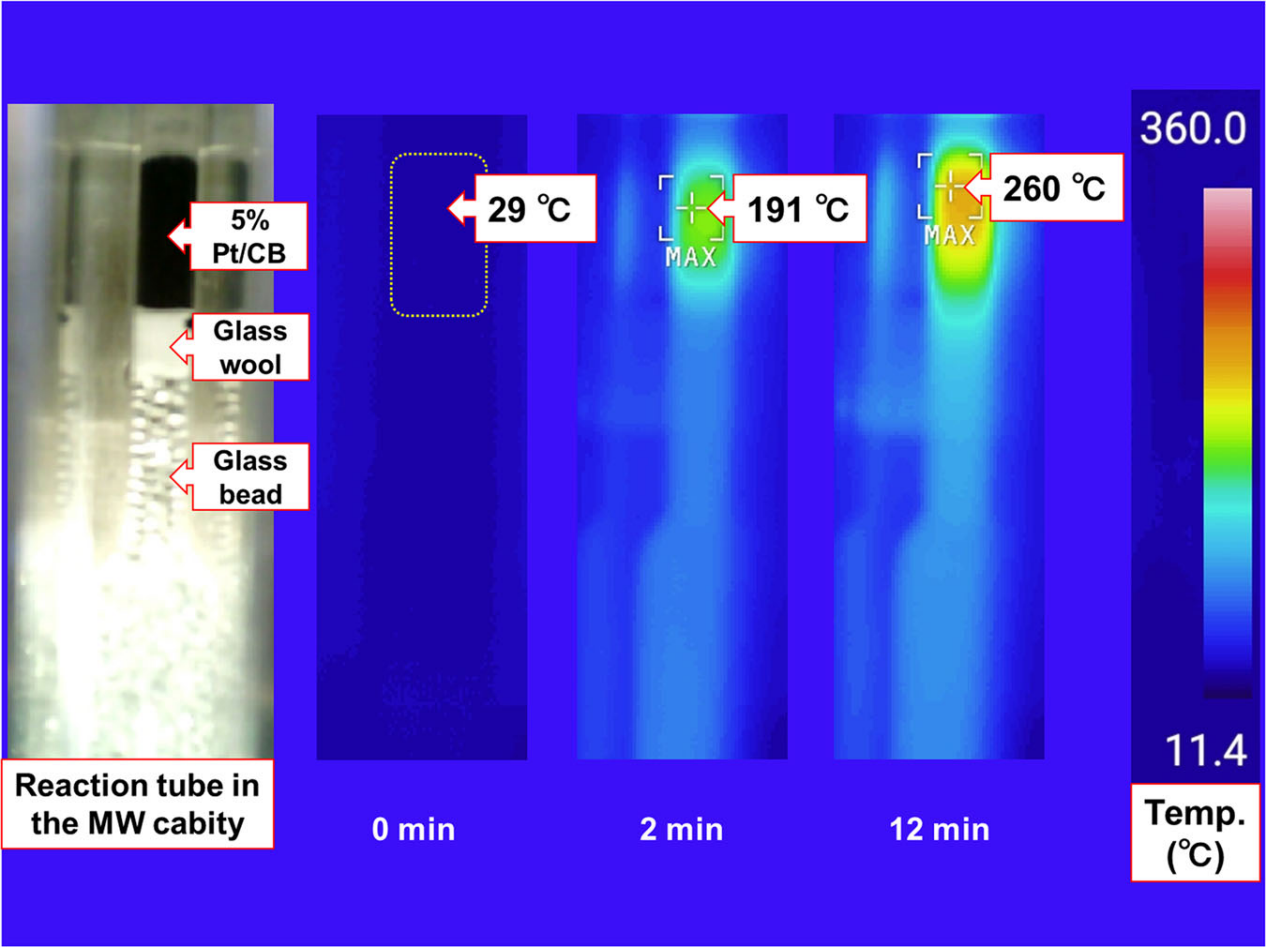
Selective MW-absorption effect on Pt/CB. Temperature transition of 5% Pt/CB under 10 W single mode-MW irradiation after 0, 2, and 12 min.

We have focused on the development of heterogeneous metal-catalyzed organic transformations under flow conditions^48,49^. In these studies, platinum on beaded activated charcoal (Pt/CB, CB; carbon bead) efficiently catalyzed the hydrogen extraction reactions of methylcyclohexanes (MCH)^50^ and 2-propanols (2-PrOH)^51^, the so-called liquid organic chemical hydrides (LOCHs)^52,53^, under single-mode MW irradiation conditions (Fig. 1c). These reactions proceeded under only 10–15 W MW irradiation, creating a local high-temperature reaction field on Pt/CB, using a selective MW-absorption effect (energy absorption of >90%) in beaded activated charcoal (Fig. 2)^54^. The flow system contributed to the smooth supply of reactants into the Pt/CB-packed cartridge and the immediate discharge of products from the local high-temperature reaction field to prevent side reactions^55^.

Taking advantage of the MW-assisted dehydrogenation (hydrogen generation) reaction of LOCHs, this study aimed to hydrogenate multiple bonds. An MCH solution of diphenylacetylene was pumped into a 10 W MW-irradiated 5% Pt/CB-packed catalyst cartridge. Phenanthrene and diphenylethane were obtained in yields of 49 and 40%, respectively (Fig. 1d). To the best of our knowledge, this direct transformation^56^ has not been reported thus far. Based on these unexpected but excellent results, the MW-mediated and Pt-catalyzed redox-neutral C–C bond formation of diarylacetylenes and dehydrogenative coupling of aromatic hydrocarbons without activated functional groups is developed for the first time (Fig. 1e). Unlike existing microwave-assisted organic synthesis in which reagents and solvents are heated by multi-frequency MW irradiation, in the present Pt/CB catalyst continuous flow reaction, a single-frequency 10 W MW was selectively absorbed (>90%) by the CB to generate a localized high-temperature reaction field at 5% Pt/CB inside the catalyst cartridge. In addition, continuous-flow reaction systems minimize non-negligible side reactions and byproducts. Thus, the present MW-assisted continuous-flow reaction exhibits high energy efficiency and can potentially be an environmentally benign chemical transformation method-, allowing input energy to be utilized without waste.

## Results and discussion

### Effects of platinum group metals and CB of catalysts

Initially, the effect of the catalyst using a single-mode MW flow apparatus was investigated (Table 1). A solution of **1a** (0.5 mmol) in MCH (0.25 M) flowed into the MW (10 W)-irradiated catalyst (80 mg)-packed glass cartridge at a flow rate of 0.25 mL/min. The 5% Pt/CB-catalyzed C–C bond formation reaction efficiently afforded **2a** in 42% yield, as well as 21% **5a** (entry 1). Catalyst efficiencies did not significantly change using 5% Pt/CB supported on different particle sizes of carbon beads (particle sizes: ~0.3 mm for 5% Pt/CB-SP and ~0.6 mm for 5% Pt/CB-LP, entries 2 and 3 vs. entry 1). The temperature of the 5% Pd/CB-packed catalyst cartridge (~270–360 °C measured with an infrared thermometer) and the reaction mixture at the cartridge outlet (~80–110 °C by a thermocouple) for Entries 1–3 also exhibited a similar trend. These results indicate that particle size does not affect the MW absorption efficiency. Furthermore, semi-hydrogenated *cis* and *trans*-stilbene were simultaneously observed in the reaction mixtures. When beaded SiO_2_ or Al_2_O_3_ was used instead of beaded carbon, the support dramatically reduced the MW energy absorption, and most of **1a** remained unchanged (entries 4 and 5). Palladium and rhodium were also ineffective as metal catalysts for MW-assisted cyclization reactions and afforded only the hydrogenated products (entries 6 and 7). Moreover, it was evident that CB without a metal catalyst had nearly no catalytic activity (Entry 8). This result strongly suggests that the present cyclization reaction does not proceed via flash pyrolysis but *via* MW-mediated Pt catalysis. The cyclization reaction using the platinum on activated carbon (powdered 5% Pt/C)-packed catalyst cartridge was also tested. 5% Pt/C in the cartridge was excessively heated during 10 W MW irradiation, and the cyclization reaction was dramatically suppressed due to a decrease in catalyst activity (entry 9 and Supplementary Fig. 7 for the excessively heated and red-colored 5% Pt/C).

**Table 1:** Effects of platinum group metals and CB of catalysts^a^.


Entry	Catalyst	Yield (%)^b^				
		1a	2a	3a	4a	5a
1	5% Pt/CB	0	42	2	7	21
2	5% Pt/CB-SP^*c*^	0	49	0	2	19
3	5% Pt/CB-LP^*d*^	0	45	2	6	24
4	5% Pt/SiO_2_(Bead)	100	0	0	0	0
5	5% Pt/Al_2_O_3_(Bead)	91	1	2	7	3
6	5% Pd/CB	79	0	1	4	3
7	5% Rh/CB	29	0	2	10	26
8	CB	76	0	1	6	1
9^*e*^	5% Pt/C	61	1	1	7	13

^a^Under constant single-frequency microwave (10 W) irradiation.

^b^Determined by ^1^H NMR spectroscopy using 1,4-dioxane (0.5 mmol) as an internal standard.

^c^SP: Smaller particles.

^d^LP: larger particles.

^e^5% Pt/C (40 mg, powdered) was used.

### Effect of temperatures in the catalyst cartridge

**Fig. 3:**
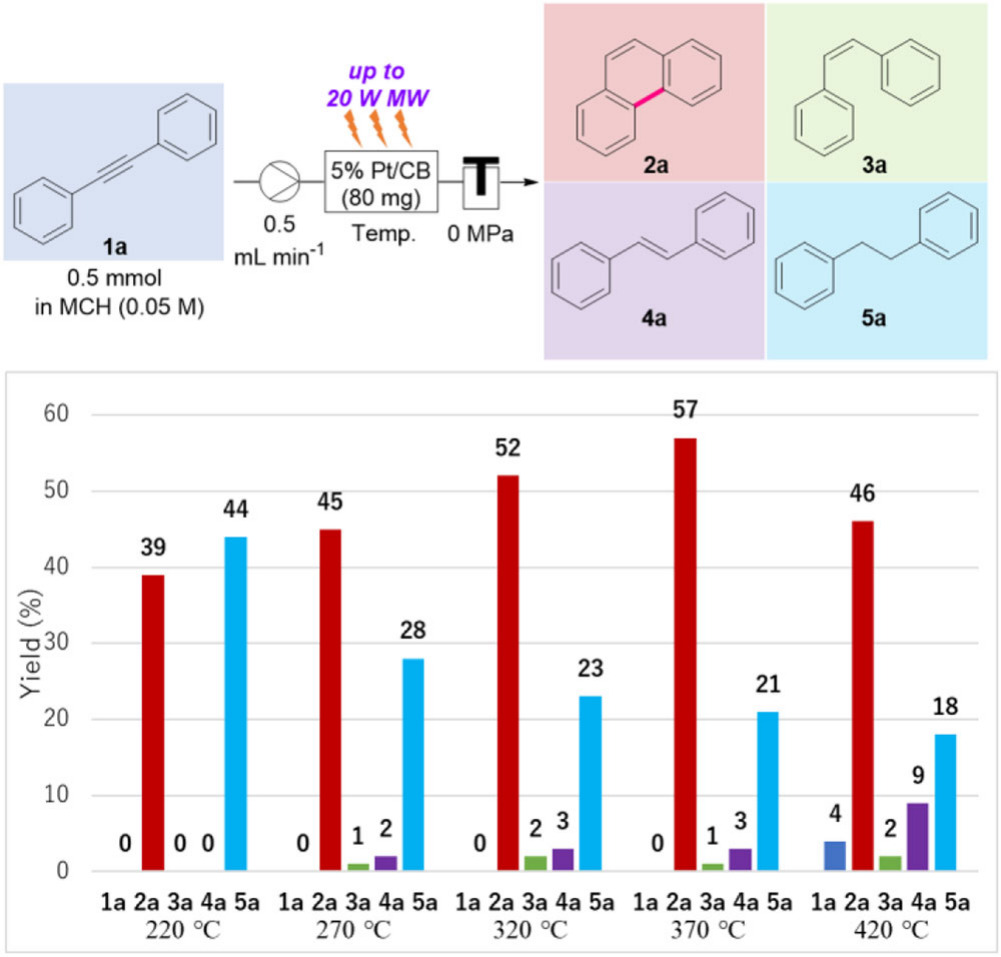
Effect of temperatures in the catalyst cartridge. Composition of the substrate (**1a**) and products (**2a**–**5a**) under MW-assisted flow, isothermal conditions. Yields were determined by ^1^H NMR using 1,1,2,2-tetrachloroethane (0.5 mmol) as an internal standard.

The product ratios at various temperatures (220–420 °C) in the catalyst cartridge were further studied under MW-intensity-controlled isothermal conditions (Fig. 3). A solution of **1a** (0.5 mmol) in MCH (0.05 M) was introduced into a 5% Pt/C (80 mg)-packed cartridge at a flow rate of 0.5 mL/min under MW-irradiation. **1a** (dark blue bar) was completely consumed to afford cyclized phenanthrene (**2a**, red bar) and hydrogenated diphenylethane (**5a**, pale blue bar) in a 39:44 ratio at 220 °C. As the temperature increased from 220 to 370 °C, **5a** gradually decreased, and conversely, the yield of **2a** increased to 57%, accompanied by a small amount of *cis*- and *trans*-alkenes (**3a**, green bar, and **4a**, violet bar, respectively). When the catalyst cartridge was heated at 420 °C, 4% of **1a** was detected in the product and the yield of **2a** was reduced to 46%. These trends indicate that hydrogenation of **1a** to form **5a** and cyclization to **2a** proceeded in a similar ratio at a relatively low temperature (220 °C). However, the MW-assisted Pt/CB-catalyzed cyclization reaction (generation of **2a**) was dominant over the hydrogenation of **1a,** **3a**, and **4a** as the temperature was gradually increased from 220 to 370 °C. In contrast, at an extremely high temperature (420 °C), the reactant diphenylacetylene (**1a**) and yields of semi-hydrogenated alkenes (**3a** and **4a**) increased. Therefore, the progress of the MW-assisted Pt/CB-catalyzed cyclization reaction is suppressed at 420 °C. Because MCH and 2-PrOH are completely vaporized at 420 °C, and the dehydrogenation reaction proceeds rapidly, the contact efficiency of the reaction mixture with the catalyst bed is probably poor owing to the significantly shortened residence time.

### Effects of solvent and co-solvent

Subsequently, the solvent and co-solvent effects on the reaction were investigated (Table 2). A solution (0.05 M) of **1a** (0.5 mmol) was pumped into the 5% Pt/CB (80 mg)-packed glass cartridge at 320 °C at a flow rate of 0.5 mL min^−1^ under MW (10 W)-irradiation. When MCH was used as the flow solvent, **2a** formed in 52% yield, whereas **2a** generated in 1% yield in toluene (entries 1 vs. 2). H_2_ gas may play an essential role in the progress of the MW-assisted C–C bond formation reaction because it is generated when using MCH as a flow solvent by MW-mediated dehydrogenative aromatization^46^.

**Table 2:** Solvent and co-solvent effects^a^.


Entry	Solvent/co-solvent	Yield (%)^b^				
		1a	2a	3a	4a	5a
1^c^	MCH/–	0	52	2	3	23
2	Toluene/–	97	1	1	5	0
3	2-PrOH/–	24	6	4	6	44
4	2-PrOH/–^d^	0	79	0	1	8
5	MCH/2-PrOH	0	82	0	0	6
6	MCH/Acetone^e^	0	87	0	0	9
7	MCH/2-BuOH^f^	0	85	0	0	8
8	MCH/1-BuOH^f^	0	19	0	4	72
9	MCH/t-BuOH^f^	0	68	1	3	16
10	MCH/Butanone	0	64	0	4	29

^a^A constant temperature was maintained by automatically controlling microwave irradiation.

^b^Determined by 1H NMR spectroscopy using 1,1,2,2-tetrachloroethane as an internal standard.

^c^Determined by ^1^H NMR spectroscopy using 1,4-dioxane (0.5 mmol) as an internal standard.

^d^The catalyst cartridge was preheated to 200 °C.

^e^A maximum MW of 40 W was irradiated.

^f^A maximum MW of 35 W was irradiated.

When 2-PrOH was used as the flow solvent, the reaction efficiency of the MW-assisted Pt/CB-catalyzed cyclization reaction was significantly reduced (the yield of **2a** was 6%), and the hydrogenated product (**5a**) was dominant (44% yield), and 24% of unreacted **1a** remained (entry 3). However, under MW irradiation, by preheating the catalyst cartridge to 200 °C before flowing the **1a** solution in 2-PrOH, **2a** generated in 79% yield (entry 4). Because of the high loss factor (tan*δ*) of 2-PrOH, the irradiated MW was absorbed by CB in the catalyst cartridge and by 2-PrOH. Therefore, CB did not reach a sufficient temperature for the cyclization reaction^40^. Therefore, in addition to the Pt metal, activated carbon, and MW, the temperature is an essential factor for efficient cyclization. The combined use of MCH with 2-PrOH, acetone, or 2-butanol (2-BuOH) as a co-solvent resulted in a high reaction efficiency, affording **2a** in 82%, 87%, and 85% yields, respectively (entries 5–7). In contrast, the addition of 1-BuOH as a co-solvent led to a lower cyclization yield (19%), whereas the hydrogenated product (**5a**) obtained 72% yield. Carbon monoxide (CO) generated by the decarbonylation of 1-BuOH via 1-PrCHO produced by Pt/CB-catalyzed dehydrogenation may block the active sites of the Pt metal by coordination. Generated CO was detected by GC-TCD (Supplementary Fig. 8). C–C bond formation proceeded smoothly when *t*-BuOH or 2-butanone was used as the co-solvent (entries 9 and 10). Other co-solvents, such as tetrahydrofuran, cyclopentyl methyl ether, ethanol, 2-pentanol, or 3-pentanol, did not significantly enhance the yield of **2a**. The effect of the MCH/2-PrOH ratio and the flow rate was also studied, and experimental results are provided in Supplementary Tables 1–3.

### Effect of hydrogen gas

**Fig. 4:**
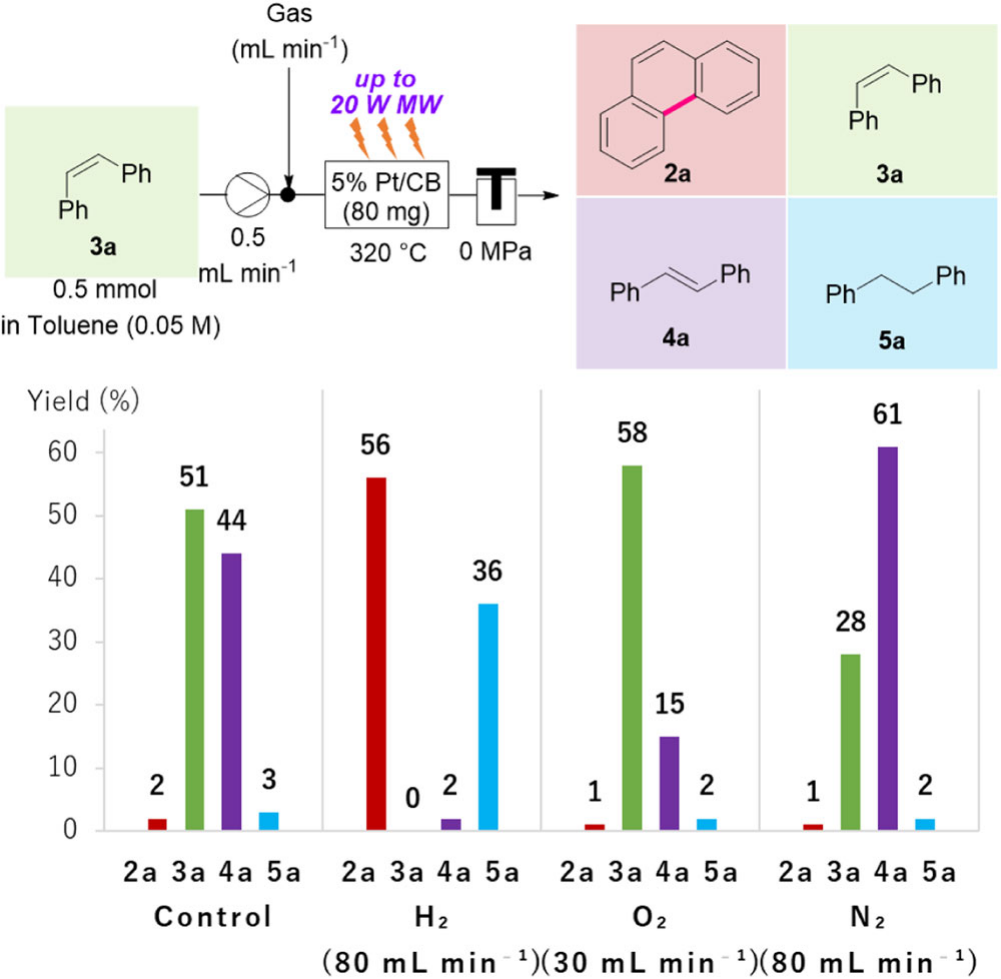
Effect of hydrogen gas. Composition of the substrate (**1a**) and products (**2a**–**5a**) under MW-assisted and hydrogen, oxygen, or nitrogen gas flow conditions and control experiment. Yields were determined by ^1^H NMR using 1,1,2,2-tetrachloroethane (0.5 mmol) as an internal standard.

Next, MW flow reactions were conducted using a solution of **3a** (0.5 mmol, green bar) in toluene (0.05 M) under various gas flow conditions to obtain further insight into the role of H_2_ gas (80 mL min^−1^) (Fig. 4). The target phenanthrene (**2a**, red bar) was obtained only under H_2_ gas conditions. Conversely, the corresponding isomerization product (**4a**, violet bar) was obtained under O_2_ or N_2_ gas conditions in 15 and 61% yields, respectively, similar to the control experiment in Fig. 4 (Control). Therefore, it is evident that H_2_ gas efficiently activates the Pt metal^57^ and promotes cyclization. It is also revealed that the reaction efficiency was improved by increasing hydrogen gas addition (Supplementary Table 4).

### Mechanistic investigations

**Fig. 5:**
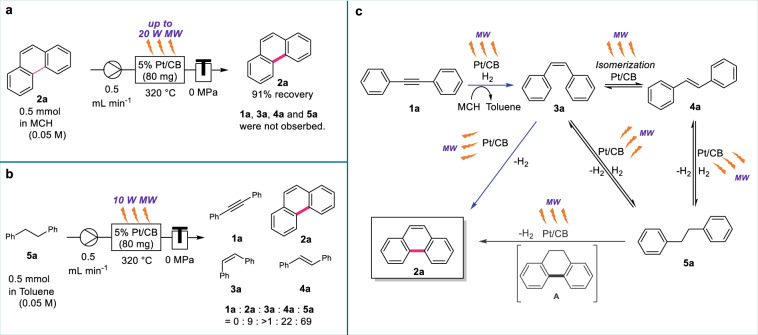
Control experiments and proposed reaction pathways. **a** The stability of **2a** under MW-assisted C–C bond formation reaction conditions. **b** MW-assisted C–C bond formation reaction using **5a** as a starting material. **c** Proposed reaction pathways.

To gain further insight into the MW-assisted C–C bond formation reaction mechanism, **2a**–**5a** were each used as a substrate in the reaction (Table 3 and Supplementary Tables 4 and 5). **2a**–**5a** in MCH/2-PrOH (2/1, 0.05 M) were pumped into the catalyst cartridge under the same flow conditions shown in Table 2, entry 5. *Cis* and *trans*-stilbenes (**3a** and **4a**) and diphenylethane (**5a**) were similarly annulated to afford **2a** in 82%, 71%, and 82% yields, respectively (entries 2–4). When **2a** was used as the substrate, **3a,** **4a**, and **5a** were not obtained, and 91% of the unchanged **2a** was recovered (Fig. 5a). Moreover, the dehydrogenation of **5a** was confirmed to proceed to production **2a** and **4a** under the same flow reaction conditions (Fig. 5b). Notably, diphenylacetylene (**1a**) was not observed in any case.

**Table 3:** Mechanistic investigations^a^.


Entry	Starting material	Yield (%)^*b*^				
		1a	2a	3a	4a	5a
1		0	82	0	0	6
		(Data from Table 2, entry 5)				
2		0	82	0	1	7
3		0	71	0	0	8
4		0	82	0	0	7

^a^A constant temperature was maintained by automatically controlling microwave irradiation.

^b^Determined by ^1^H NMR spectroscopy using 1,1,2,2-tetrachloroethane as an internal standard.

Based on these results, reaction mechanisms were proposed for the Pt-catalyzed redox-neutral C–C bond formation of diphenylacetylene under MW irradiation (Fig. 5c). Using the H_2_ gas generated by the Pt-catalyzed dehydrogenation of MCH under MW irradiation, **1a** was simultaneously semi-hydrogenated by the 5% Pt/CB catalyst to **3a**. Subsequently, the MW-mediated 5% Pt/CB-catalyzed dehydrogenative coupling of **3a** proceeded to afford the thermodynamically stable phenanthrene **2a**. A portion of **3a** was isomerized to **4a** at equilibrium, and alkenes (**3a** and **4a**) were further hydrogenated to the corresponding alkane (**5a**). Because the isomerization and hydrogenation of **3a** and **4a**, as well as the dehydrogenation of **5a**, are in equilibrium under MW irradiation conditions using the 5% Pt/CB-packed catalyst cartridge, **3a** was regenerated from **4a** and **5a** during the reaction process. It is possible that cycloaromatization *via* the formation of dihydrophenanthrene (**A**) and subsequent dehydrogenation (**5a** to **2a**) proceeded simultaneously.

### Scope of substrates

**Fig. 6:**
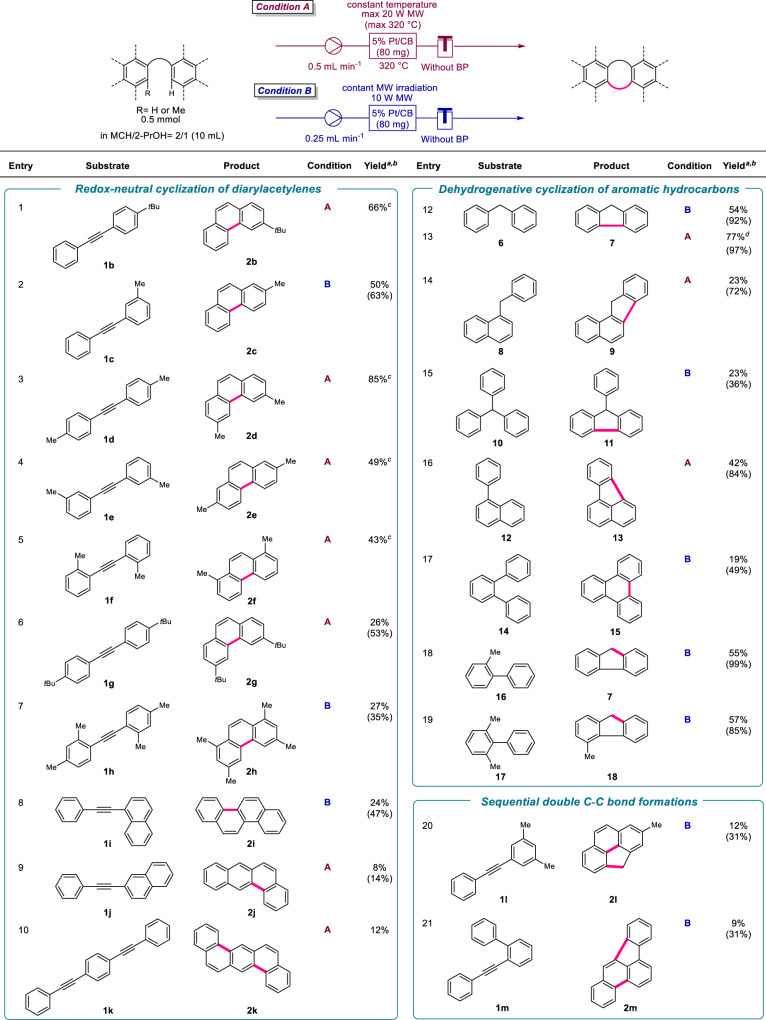
Scope of substrates. **a** The yield was determined by ^1^H NMR spectroscopy using 1,1,2,2-tetrachloroethane as an internal standard. **b** The yield based on the consumed starting material is in parentheses. **c** The starting material was completely consumed. **d** The flow rate was 0.25 mL min^−1^.

Next, the redox-neutral (oxidant-free) C–C bond formation reaction of various diarylacetylenes and aromatic hydrocarbons was conducted under isothermal or definite MW-irradiation conditions (Fig. 6). Asymmetrical 4-*tert*-butyl (*t*Bu)- or 3-methyl (Me)-substituted diphenyl acetylenes (**1b** and **1c**) were cyclized to the corresponding phenanthrene derivatives (**2b** and **2c**) in 66 and 50% yields, respectively (entries 1 and 2). C–C bond formation of 4,4’-, 3,3’-, or 2,2’-dimethyldiphenylacetylenes (**1d,** **1e**, and **1f**) also proceeded to afford the corresponding phenanthrenes (**2d,** **2e**, and **2f**) in 43–85% yields (entries 3–5). Although the cyclization of 4,4’-ditertiarybutyldiphenylacetylene (**1g**) and 2,2’,4,4’-tetramethyldiphenylacetylenes (**1h**) was relatively difficult owing to the low solubility of both the substrate and product. This caused physical adsorption on the CB catalyst support, and cyclized products (**2g** and **2h**) were obtained in 26 and 27% yields, respectively (entries 6 and 7). The solubility of products is essential, and thereby, 1-naphtylphenylacetylene (**1i**), 2-naphtylphenylacetylene (**1j**), and 1,4-bis(phenylethynyl)benzene (**1k**) were transformed to chrysene (**2i**), benz[*a*]anthracene (**2j**), and dibenz[*a*,*h*]anthracene (**2k**), which are low solubility materials, in MCH/2-PrOH in relatively low yields [24% (47% based on consumed starting material), 8% (14% based on consumed starting material), and 12% yields, respectively] (entries 8–10). The MW-assisted Pt/CB-catalyzed reaction was efficient for the dehydrogenative intramolecular coupling of various aromatic hydrocarbons. Decreasing the flow rate was effective in the case of the cyclization of diphenylmethane (**6**), and the yield of fluorene (**7**) was increased up to 77% (entry 12 vs. 13). Triarylmethanes (**8** and **10**) was also annulated to afford corresponding benzo[*a*]fluorenes (**9**) and 9-phenylfluorene (**11**) in each 23% yields (entries 14 and 15). Fluoranthene (**13**) and triphenylene (**15**) formed in 42 and 19% yields (84 and 49%, respectively, based on the consumed starting materials) from 1-phenylnaphthalene (**12**) and *o*-terphenyl (**14**), respectively (entries 16^58^ and 17^14,15,59^). Furthermore, the present MW-assisted C–C bond formation reaction could be applied to intramolecular dehydrogenative C(sp^3^)–H–C(sp^2^)–H coupling (cyclization) reactions without oxidants, radical-generating reagents or photosensitizers^60–64^. 2-Methylbiphenyl (**16**) and 2,6-dimethylbiphenyl (**17**) were transformed into the corresponding fluorene (**7**) and 4-methylfluorene (**18**) in 55 and 57% yields (99 and 83%, respectively, based on the consumed starting materials) (entries 18 and 19). 1,3-Dimethyl-5-(2-phenylethynyl)benzene (**1l**) and 1-(*o*-biphenylyl)-2-phenylethyne (**1m**) were transformed into 2-methyl-4H-cyclopenta[*d*,*e*,*f*]phenanthrene (**2l**) and benzo[*b*]fluoranthene (**2m**), respectively, *via* two sequential redox-neutral dehydrogenative cyclization reactions (entries 20 and 21). Although the starting materials were not completely converted by the catalyst in many cases, it is noteworthy that the yields of the MW-assisted Pt/CB-catalyzed cyclization products based on the consumed starting materials were relatively high (entries 2, 6–8 and 12–21).

### Reuse of 5% Pt/CB

**Fig. 7:**
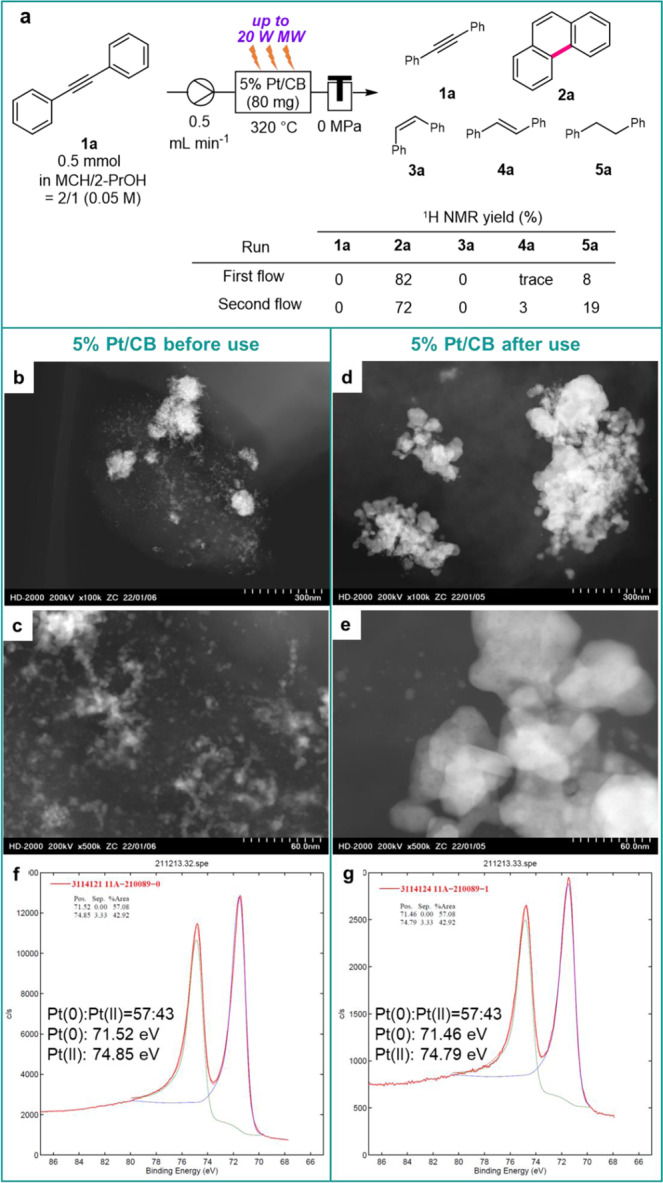
Reuse test of 5% Pt/CB. **a** The reuse test of 5% Pt/CB under MW-assisted C–C bond formation reaction using **1a** as a starting material. **b**–**e** STEM image of 5% Pt/CB before and after use. **f**, **g** XPS spectra of 5% Pt/CB before and after use.

The reuse of a 5% Pt/CB-packed cartridge was examined under the MW-assisted C–C bond formation reaction conditions. Equal volumes of MCH/2-PrOH solution of **1a** have flowed twice into a 5% Pt/CB-packed cartridge. In the second flow, the yield of **2a** was slightly decreased, and the formation of diphenylethane (**5a**), in which the alkyne of **1a** was hydrogenated, increased (Fig. 7a, First flow vs. Second flow). Before and after use, the physical properties of 5% Pt/CB in the catalyst cartridge were evaluated by scanning transmission electron microscopy (STEM) and X-ray photoelectron spectroscopy (XPS). A mixture of small Pt clusters of approximately 1–3 nm and relatively large Pt clusters of roughly 1 µm was loaded on 5% Pt/CB before use (Fig. 7b, c), whereas sintered Pt clusters of 2–4 µm were observed after the MW-assisted continuous-flow reaction (Fig. 7d, e). XPS analysis indicated that the ratio of Pt(0) to Pt(II) in the 5% Pt/CB catalyst did not change before and after use (Fig. 7f, g). Therefore, the particle size change of Pt loaded on CB may affect the reaction efficiency of the present cyclization reaction.

## Conclusion

In this study, MW-assisted and Pt/CB-catalyzed C–C bond formation of diarylacetylenes under continuous-flow conditions was developed. A highly activated local and high-temperature reaction field was generated on Pt/CB in the catalyst cartridge by selective (>90%) MW energy absorption in CB. Therefore, the novel transformation proceeded under redox-neutral conditions without any bases or oxidants. Various mechanistic experiments revealed that H_2_ gas plays an essential role in this transformation. Firstly, in the activation of the Pt metal. Secondly, in the promotion of the elimination of the reaction solution from the catalyst cartridge to control the residence time, preventing the generation of undesirable byproducts. In the reaction cartridge, Pt/CB-catalyzed hydrogenation of diphenylacetylenes (**1**) and *cis* or *trans*-diphenylethylenes (**3** and **4**), isomerization of diphenylethylenes, and dehydrogenation of diphenylethanes (**5**) should occur in equilibrium. Thermodynamically stable phenanthrene derivatives (**2**) were produced by a Pt/CB-catalyzed dehydrogenative C–C coupling reaction during a short MW irradiation period in the cartridge. Furthermore, this MW-assisted cyclization reaction was applicable to dehydrogenative C(sp^2^)–C(sp^2^) and C(sp^2^)–C(sp^3^) bond formation reactions for the synthesis of fused aromatics, such as chrysene, fluorene, ben-zo[*a*]fluorene, fluoranthene, and triphenylene. This Pt/CB-catalyzed continuous-flow reaction exhibited high energy efficiencies; that is, only 10–20 W of MW energy was required to obtain hundreds of degrees at a local high-temperature reaction field in the flow reaction cartridge. Notably, pre-functionalization of the starting material, such as oxidation, halogenation, or metalation, is not required. Therefore, the MW-assisted reaction is an environmentally ideal transformation methodology, and the input energy can be accumulated without waste. These findings may contribute to the development of a new generation of chemical technology and the crisis of organic semiconductor shortage caused by COVID-19. Investigation of the detailed reaction mechanism of Pt/CB-catalyzed C–H activation under MW-assisted conditions and improvement of MW flow devices aiming for their practical applications are ongoing.

## Methods

### General methods

For Instrumentation and Materials, see Supplementary Method 1. For Additional experiments concerning optimization of the reaction conditions, the effect of external addition of hydrogen gas, gas detection experiment, and additional mechanistic investigations related to Table 3, see Supplementary Methods 4–7.

### General procedures for MW-assisted and Pt/CB-catalyzed cyclization reactions

See below and Supplementary Method 8.

### The reaction using EYELA, MR-2G-100 (Condition A)

The entire flow path was fitted with the 5% Pt/CB (80.0 mg)-packed EYELA reaction tube and filled with a mixed solvent of MCH/2-PrOH (2/1). The mixed solvent was pumped through the reaction tube at a flow rate of 0.5 mL min^−1^ at 320 °C under a maximum of 20 W MW irradiation for 5 min. A solution of **1** in the mixed solvent (0.05 M) was pumped into the reaction tube, and then the solution vessel was sequentially rinsed with the mixed solvents four times (1 mL ×3, then 20 mL ×1) using a pump. The MW irradiation was then stopped, and ethyl acetate/toluene (1/1, 40 mL) or dichloromethane (40 mL) was pumped to further wash the entire flow path. The entire reaction mixture and washing solution were collected and concentrated in vacuo, dissolved in deuterated chloroform (CDCl_3_), and analyzed by ^1^H NMR spectroscopy using 1,1,2,2-tetrachloroethane (52.5 µL, 0.5 mmol) as an internal standard.

### The reaction using SAIDA, FMR-100 (Condition B)

The entire flow path was fitted with the 5% Pt/CB (80.0 mg)-packed SAIDA reaction tube and filled with a mixed solvent of MCH/2-PrOH (2/1). The mixed solvent was pumped through the reaction tube at a flow rate of 0.5 mL min^−^^1^ under a maximum of 10 W MW irradiation for 5 min. A solution of **1** in the mixed solvent (0.05 M) was pumped into the reaction tube, and then the solution vessel was sequentially rinsed with the mixed solvents four times (1 mL ×3, then 20 mL ×1) using a pump. The MW irradiation was then stopped, and ethyl acetate/toluene (1/1, 40 mL) or dichloromethane (40 mL) was pumped to further wash the entire flow path. The whole reaction mixture and washing solution were collected and concentrated in vacuo, dissolved in deuterated chloroform (CDCl_3_), and analyzed by ^1^H NMR spectroscopy using 1,1,2,2-tetrachloroethane (52.5 µL, 0.5 mmol) as an internal standard.

### The reaction using EYELA, MR-2G-100 under external addition of hydrogen gas conditions (Condition C)

The entire flow path was fitted with the 5% Pt/CB (80.0 mg)-packed EYELA reaction tube and filled with toluene. Toluene (0.5 mL min^−1^) and hydrogen gas (80 mL min^−1^) were pumped through the reaction tube at a flow rate of 0.5 mL/min at 320 °C under a maximum of 20 W MW irradiation for 5 min. A solution of **3a** in toluene (0.05 M) was pumped into the reaction tube, and then the solution vessel was sequentially rinsed with toluene four times (1 mL ×3, then 20 mL ×1) using a pump. The MW irradiation was then stopped, and ethyl acetate/toluene (1/1, 40 mL) was pumped to further wash the entire flow path. The whole reaction mixture and washing solution were collected and concentrated in vacuo, dissolved in deuterated chloroform (CDCl_3_), and analyzed by ^1^H NMR spectroscopy using 1,1,2,2-tetrachloroethane (52.5 µL, 0.5 mmol) as an internal standard.

### MW flow devices (SAIDA and EYELA), catalyst cartridges, and peripheral devices

See Supplementary Method 2, Supplementary Figs. 1–6.

### Preparation of substrates

See Supplementary Method 9, Supplementary Figs. 9–11.

### Spectroscopic data of products

See Supplementary Method 10, Supplementary Figs. 12–79.

## Supplementary information

Supplementary Information

## Data Availability

The authors declare that the data supporting the findings of this study are available within the paper or its Supplementary Information files and from the corresponding author upon request.
