# Supplementary material for: Microwave-assisted C–C bond formation of diarylacetylenes and aromatic hydrocarbons on carbon beads under continuous-flow conditions
Source: Commun Chem. 2023 Apr 24;6:78. doi: 10.1038/s42004-023-00880-y (PMC10123573; doi:10.1038/s42004-023-00880-y)
Supplement: Supplementary file 1 — Supplementary Information [file 42004_2023_880_MOESM1_ESM.pdf]

Supplementary Information

Microwave-assisted C–C bond formation of diarylacetylenes and aromatic hydrocarbons on carbon beads under continuous-flow conditions

Tsuyoshi Yamada[1], Wataru Teranishi[1], Naoya Sakurada[1], Seiya Ootori[1], Tomohiro Matsuo[1], Yuka Abe[1], Yasuharu Morii[2], Masatoshi Yoshimura[3], Takeo Yoshimura[4], Takashi Ikawa[1] and Hironao Sajiki[1]*

[1]Laboratory of Organic Chemistry, Gifu Pharmaceutical University 1-25-4 Daigaku-nishi, Gifu 501-1196, Gifu, Japan.

[2]Product division, Tokyo Rikakikai Co., Ltd. (Brand: EYELA), 1-15-17 Koishikawa, Bunkyo-ku 112-0002, Tokyo, Japan.

[3]R&D Center, N.E. Chemcat Corporation, 678 Ipponnmatsu, Numazu 410-0314, Shizuoka Japan.

[4]SAIDA FDS INC., 143-10 Isshiki, Yaizu 425-0054, Shizuoka, Japan.

*E-mail: Hironao Sajiki : sajiki@gifu-pu.ac.jp

Table of Contents

Supplementary Methods

1. Instrumentation and Materials
2. MW flow devices (SAIDA and EYELA), catalyst cartridges, and peripheral devices
3. Picture of 5% Pt/C (powdered) in catalyst cartridge under 10W MW irradiation
4. Optimization of the reaction conditions
5. Effect of external addition of hydrogen gas
6. Gas detection experiment
7. Additional mechanistic investigations related to Table 3
8. General procedures for MW-assisted and Pt/CB-catalyzed cyclization reactions
9. Preparation of substrates
10. Spectroscopic data of products

^{1}H and ^{13}C NMR spectra of products

Supplementary references

Supplementary Methods

1. Instrumentation and Materials

The substrates and solvents were purchased from commercial sources and used without further purification unless otherwise noted. Flash column chromatography was performed with Silica Gel 60 N (Kanto Chemical Co., Inc., 40–50 and 63–210 μm spherical, neutral). ^{1}H and ^{13}C NMR spectra were recorded on a JEOL ECZ 400 (^{1}H: 400 MHz, ^{13}C: 100 MHz) or ECA 500 spectrometer (^{1}H: 500 MHz, ^{13}C: 125 MHz) at room temperature in $CDCl_3$ as a solvent and an internal standard (^{1}H NMR: δ = 0.00 for TMS, ^{13}C NMR: δ = 77.0 for $CDCl_3$). IR spectra were recorded by Brucker FT-IR ALPHA. High-resolution mass spectra (HRMS) were measured by JEOL JMS-T100TD (DART-TOF-MS). Melting points were measured by a SANSYO SMP-300 melting point apparatus. The microwave flow reactor (SDIDA, FMR-100) was developed by Saida FDS Inc., Shizuoka, Japan; see the product URL: https://www.saidagroup.jp/fds_en/microwave/product. The continuous flow microwave reactor (EYELA, MR-2G-100) was developed by Tokyo Rikakikai Co., Ltd., Tokyo, Japan; see the product URL: https://eyela.actibookone.com/content/detail?param=eyJjb250ZW50TnVtIjoyMTY4MX0=&detailFlg=1&pNo=44. The straight glass and quartz tube as catalyst cartridges were also obtained from Saida FDS Inc., and Tokyo Rikakikai Co., Ltd. LC-20AD (Shimadzu Co., Kyoto, Japan) was also used for flowing a reaction solution. EYELA MFC-11GU (Tokyo Rikakikai Co., Ltd., Tokyo, Japan) for hydrogen gas and KOFLOC MODEL 8500 (KOFLOC Co., Kyoto, Japan) for oxygen and nitrogen gas were used for gas mass flow controller/meters. YMH-500LF (YMC Co., Ltd., Kyoto, Japan) was used for the hydrogen storage alloy canister. GC-3200 [gas chromatograph equipped with thermal conductivity detector (GC-TCD; GL Science, Japan)] was used for gas analysis with Molecular Sieve 5Å (60/80 mesh) packed column (3 × 2.2 mm i.d., 1/8 inch: GL Science, Japan). HD-2000 (Hitachi Ltd., Tokyo, Japan) and Quantera SXM (ULVAC-PHI inc., Kanagawa, Japan) were used for scanning transmission electron microscopy (STEM) analysis and X-ray photoelectron spectroscopy (XPS), respectively. 5% Pt/CB, 5% Pt/CB-SP, 5% Pt/CB-LP, 5% Pt/Si, 5% Pt/Al_2O_3, 5% Pd/CB, 5% Rh/CB, and CB were obtained from N.E. CHEMCAT Co. (Tokyo, Japan). The ^{1}H NMR spectra of known products were identical to those in the literature.

2. MW flow devices (SAIDA and EYELA), catalyst cartridges, and peripheral devices

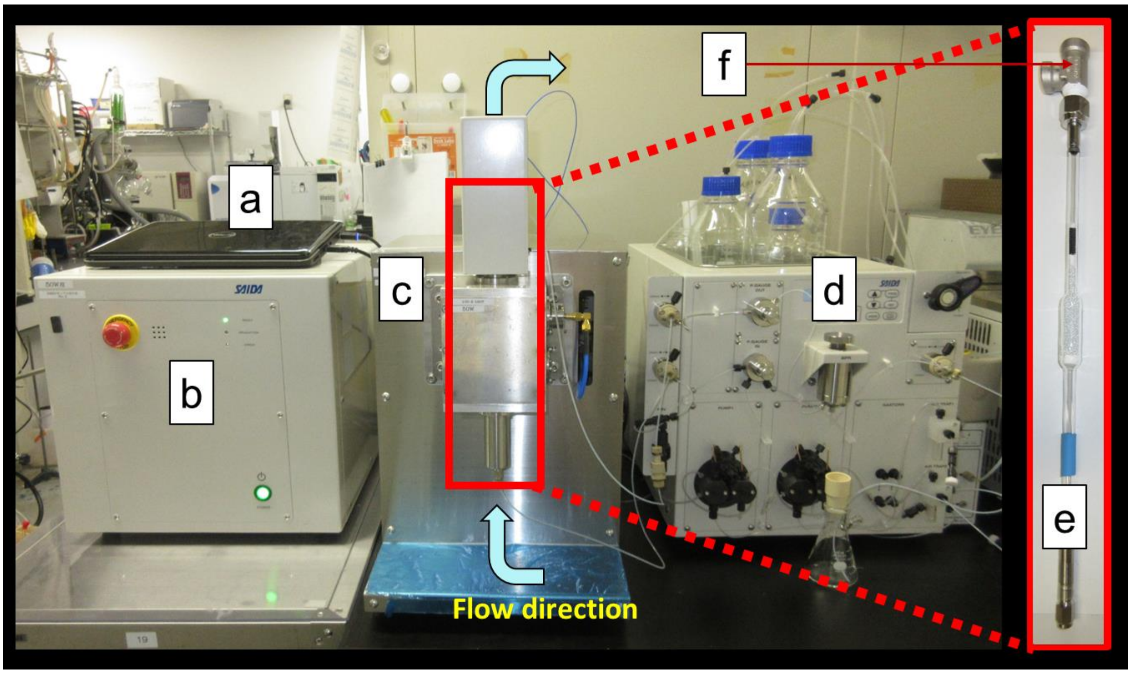

Supplementary Fig. 1 The microwave flow reactor (SDIDA, FMR-100) (a) Control PC, (b) Microwave controller unit, (c) Microwave generator unit, (d) Pump unit, (e) 5% Pt/CB-packed glass tube, (f) Thermocouple was attached outlet of the glass tube

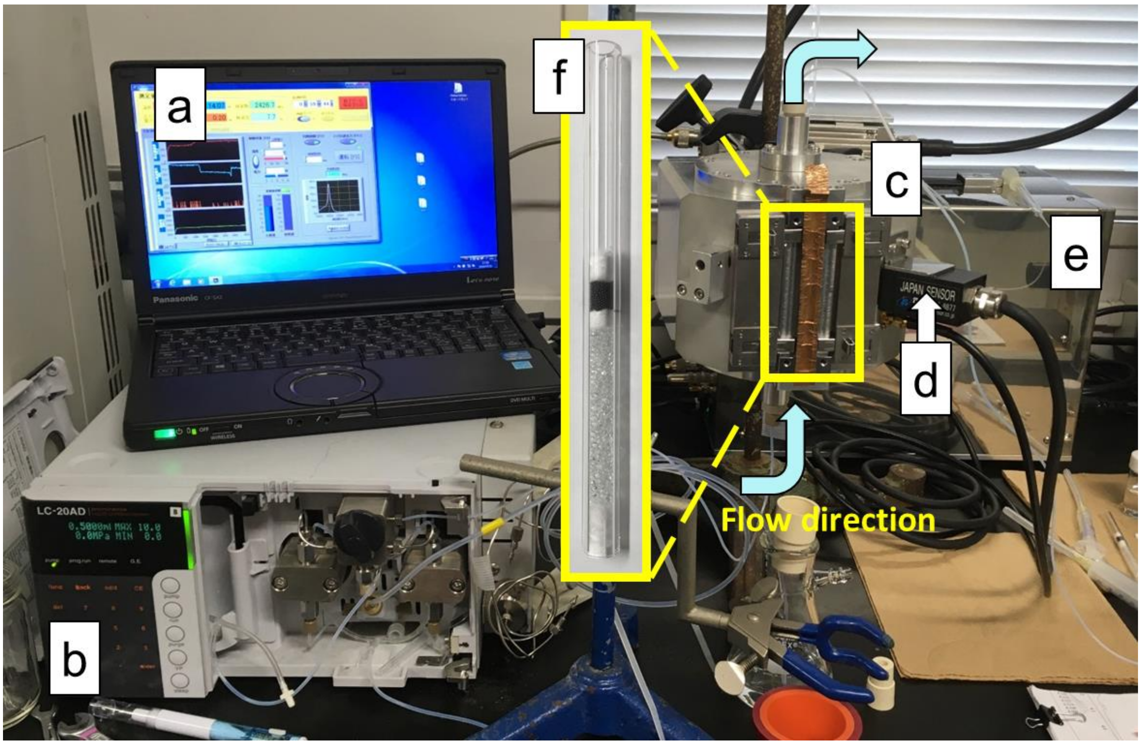

Supplementary Fig. 2 The microwave flow reactor (EYELA, MR-2G-100) (a) Control PC, (b) Pump, (c) Microwave generator unit, (d) Thermography unit, (e) Microwave controller unit, (f) 5% Pt/CB-packed Quartz tube

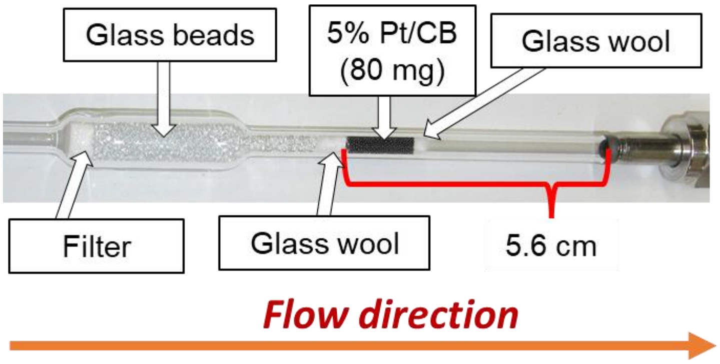

Supplementary Fig. 3 The glass tube for the microwave flow reactor (SDIDA, FMR-100)

Preparation of 5% Pt/CB-packed cartridge

Glass beads and glass wool were put on the glass filter inside a SAIDA glass reaction tube to a depth of 5.6 cm from the top to the bottom of the glass tube. 5% Pt/CB (80.0 mg) was tightly filled on the glass wool by tapping the tube, and another glass wool was then placed on the catalyst to prevent movement of the 5% Pt/CB from the cartridge.

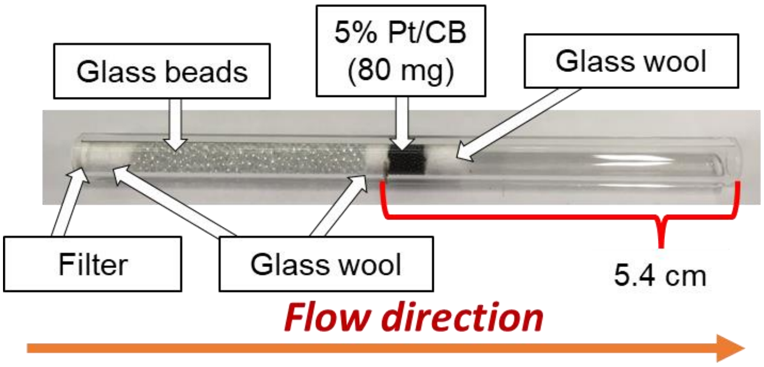

Supplementary Fig. 4 The quartz and glass tube for the continuous flow microwave reactor (EYELA, MR-2G-100)

Preparation of 5% Pt/CB-packed cartridge

Glass beads and glass wool were put on the glass filter inside an EYELA quartz reaction tube to a depth of 5.4 cm from the top to the bottom of the quartz tube. 5% Pt/CB (80.0 mg) was tightly filled on the glass wool by tapping the tube, and another glass wool was then placed on the catalyst to prevent movement of the 5% Pt/CB from the cartridge.

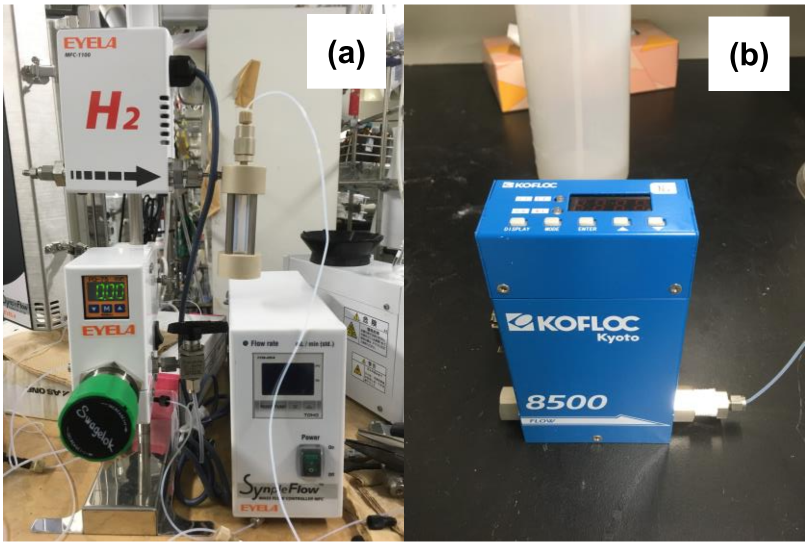

Supplementary Fig. 5 Gas mass flow controller/meter (a) EYELA MFC-11GU for hydrogen gas flow. (b) KOFLOC MODEL 8500 for oxygen and nitrogen gas flow

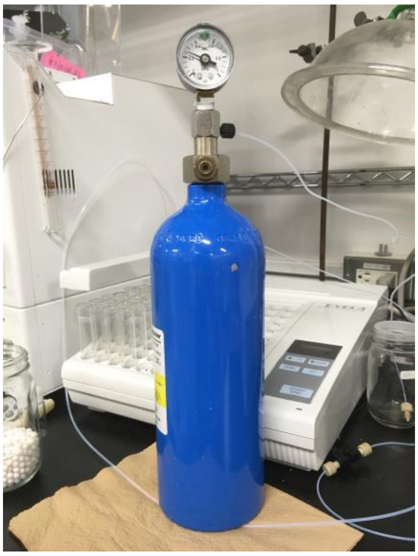

Supplementary Fig. 6 Hydrogen storage alloy canister YMH-500LF

3. Picture of 5% Pt/C (powdered) in catalyst cartridge under 10W MW irradiation

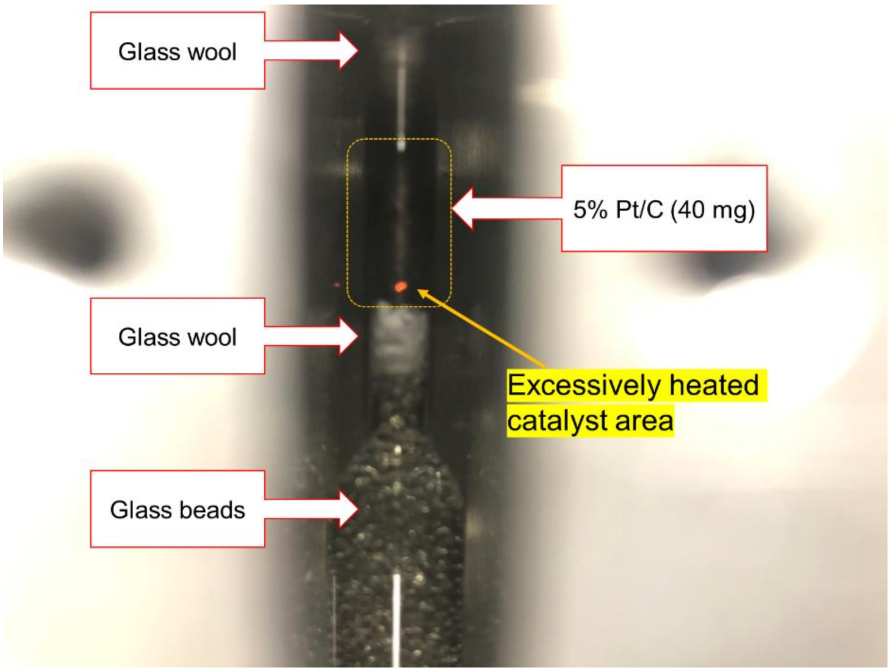

Supplementary Fig. 7 The red light was observed on the 5% Pt/C (powdered) in the catalyst cartridge by overheating

4. Optimization of the reaction conditions

Supplementary Table 1 Effect of co-solvent and its ratio

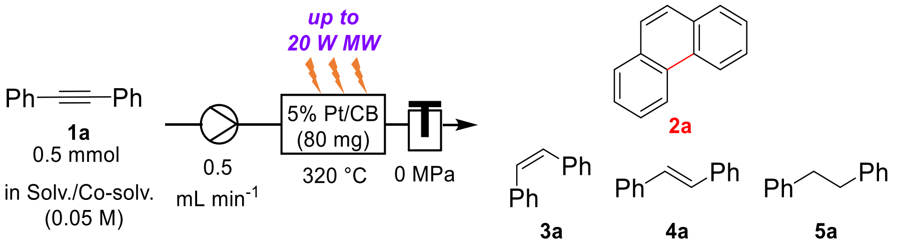

Entry	Solv./Co-solv. = 2/1	^{1}H NMR yield (%)[a)				
		**1a**	**2a**	**3a**	**4a**	**5a**
1[b)]	MCH	0	52	2	3	23
**2**	**MCH/2-PrOH**	**0**	**82**	**0**	**0**	**6**
3	Toluene/2-PrOH	19	53	3	7	17
4	*n*-Heptane/2-PrOH	8	28	5	17	38
5	MCH/THF	8	50	2	8	21
6	MCH/CPME	57	6	2	11	14
7	MCH/EtOH	0	42	1	3	40
8[c)]	MCH/1-Butanol	0	19	< 1	4	72
9[c)]	MCH/2-Butanol	0	85	0	0	8
10[c)]	MCH/*t*-Butanol	0	68	1	3	16
11[c)]	MCH/2-Pentanol	0	84	< 1	< 1	6
12[c)]	MCH/3-Pentanol	0	84	0	0	7
13[d)]	MCH/Acetone	0	**87**	< 1	< 1	9
14	MCH/2-Butanone	0	64	0	4	29
15	MCH/3-Pentanone	0	48	1	6	44

a) The yields were determined by ^{1}H NMR using 1,1,2,2-tetrachloroethane as an internal standard. b) The yield was determined by ^{1}H NMR using 1,4-dioxane as an internal standard. c) Maximum of 35 W of MW was irradiated. d) Maximum of 40 W of MW was irradiated.

Supplementary Table 2 Optimization of the ratio of MCH and 2-PrOH

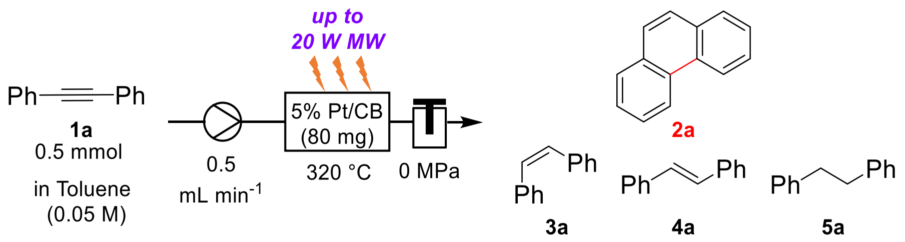

Entry	MCH/IPA	^{1}H NMR yield (%)[a]				
		**1a**	**2a**	**3a**	**4a**	**5a**
1[b]	MCH	0	52	2	3	23
2	30/1	0	66	< 1	1	13
3	10/1	0	72	< 1	1	11
4	3/1	0	77	< 1	< 1	5
**5**	**2/1**	**0**	**82**	**0**	**0**	**6**
6	1/1	0	67	0	0	19
7[c]	1/1	0	**87**	0	0	2
8[d]	1/2	0	**87**	0	0	5
9	2-PrOH	24	6	4	6	43
10[e]	2-PrOH	0	79	< 1	1	8

a) The yields were determined by ^{1}H NMR using 1,1,2,2-tetrachloroethane as an internal standard. b) The yield was determined by ^{1}H NMR using 1,4-dioxane as an internal standard. c) Maximum of 35 W of MW was irradiated. d) Maximum of 40 W of MW was irradiated. e) The catalyst cartridge was pre-heated to 200 °C.

Supplementary Table 3 Effect of flow rate

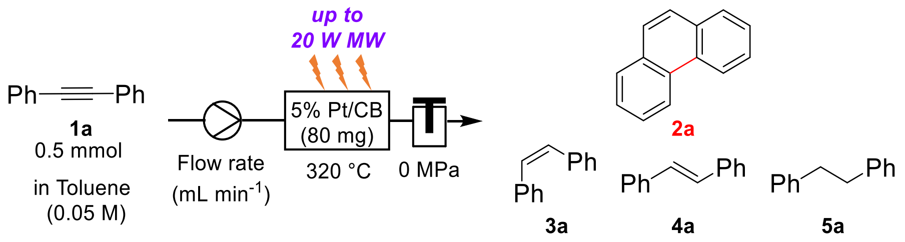

Entry	Flow rate (mL min^{-1})	^{1}H NMR yield (%)[a]				
		**1a**	**2a**	**3a**	**4a**	**5a**
1	0.25	0	78	0	0	4
**2**	**0.5**	**0**	**82**	**0**	**0**	**6**
3[b]	0.75	0	48	2	2	45

a) The yields were determined by ^{1}H NMR using 1,1,2,2-tetrachloroethane as an internal standard. b) The temperature of the catalyst cartridge reached to maximum of 250 °C.

5. Effect of external addition of hydrogen gas

Supplementary Table 4 Effect of external addition of hydrogen gas

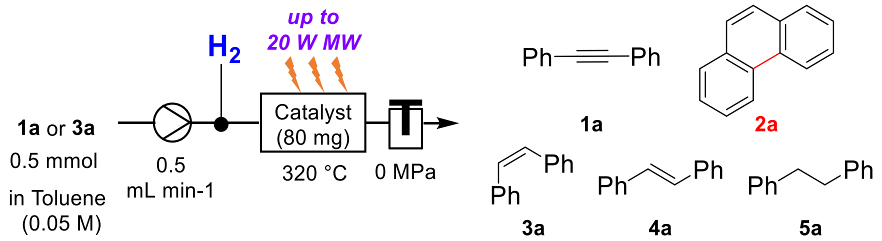

Entry	Substrate	H_2 (mL min^{-1})	^{1}H NMR yield (%)[a] 1a	2a	3a	4a	5a
1		0	97	1	1	5	< 1
2	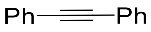	10	0	31	2	15	55
3	**1a**	40	5	47	< 1	3	45
4		80	0	63	< 1	3	28
5	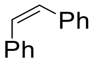	0	-	2	53	46	4
6	**3a**	80	-	56	< 1	2	36

a) The yields were determined by ^{1}H NMR using 1,1,2,2-tetrachloroethane as an internal standard.

6. Gas detection experiment

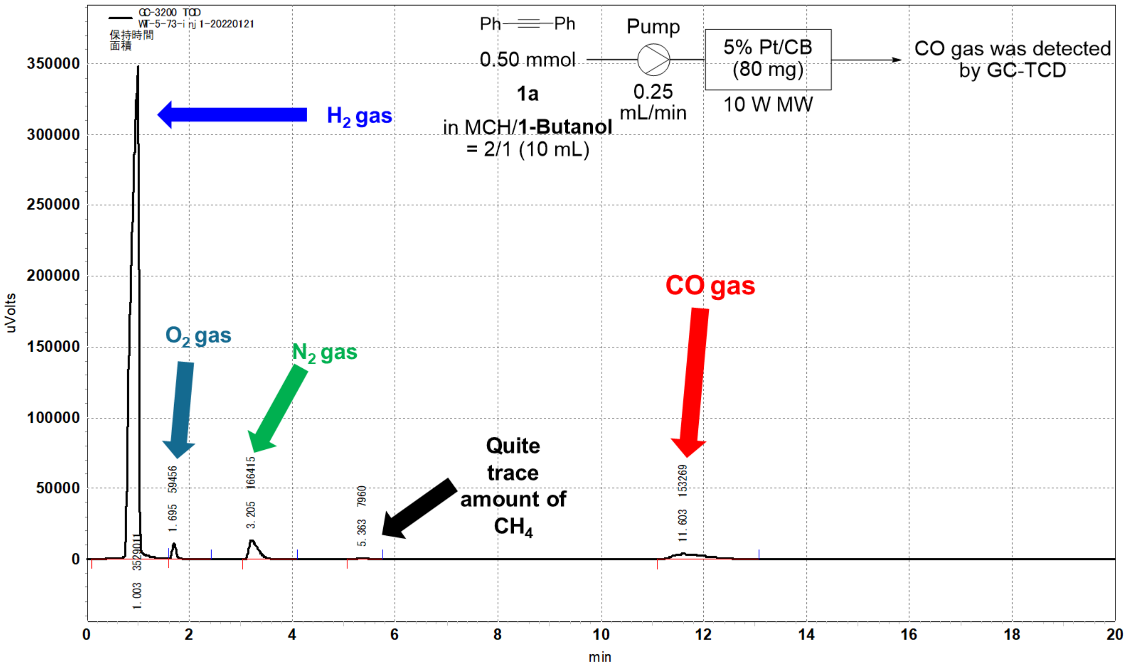

Supplementary Fig. 8 GC chart of the experiment using 1-butanol as a co-solvent (Table 2, Entry 8)

7. Additional mechanistic investigations related to Table 3

Supplementary Table 5 Additional mechanistic investigations

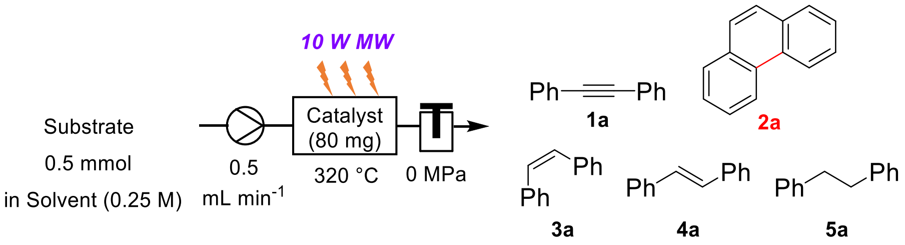

Entry	Substrate	Catalyst	Solvent	^{1}H NMR ratio (%)[a)]				
				**1a**	**2a**	**3a**	**4a**	**5a**
1	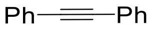 **1a**	5% Pt/CB	MCH	0	55	0	0	45
2		5% Pt/CB	Toluene	96	Trace	0	4	0
3		CB	MCH	97	0	1	2	0
4	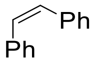 **3a**	5% Pt/CB	MCH	0	29	16	0	55
5		5% Pt/CB	Toluene	0	9	90	0	1
6		CB	MCH	0	0	62	38	0
7	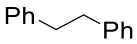 **5a**	5% Pt/CB	MCH	0	38	0	0	62
8		5% Pt/CB	Toluene	0	9	trace	22	69
9	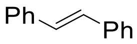 **4a**	5% Pt/CB	MCH	0	57	0	0	43
10		5% Pt/CB	Toluene	0	1	4	92	3

a) The ratio was determined by ^{1}H NMR.

8. General procedures for MW-assisted and Pt/CB-catalyzed cyclization reactions

Condition A (using EYELA, MR-2G-100): The entire flow path was fitted with the 5% Pt/CB (80.0 mg)-packed EYELA reaction tube and filled with a mixed solvent of MCH/2-PrOH (2/1). The mixed solvent was pumped through the reaction tube at a flow rate of 0.5 mL min^{-1} at 320 °C under a maximum of 20 W MW irradiation for 5 min. A solution of **1** in the mixed solvent (0.05 M) was pumped into the reaction tube, and then the solution vessel was sequentially rinsed with the mixed solvents four times (1 mL × 3, then 20 mL × 1) using a pump. The MW irradiation was stopped, and ethyl acetate/toluene (1/1, 40 mL) or dichloromethane (40 mL) was pumped to further wash the entire flow path. The whole reaction mixture and washing solution were collected and concentrated in vacuo, dissolved in deuterated chloroform ($CDCl_3$), and analyzed by ^1H NMR spectroscopy using 1,1,2,2-tetrachloroethane (52.5 µL, 0.5 mmol) as an internal standard.

Condition B (using SDIDA, FMR-100): The entire flow path was fitted with the 5% Pt/CB (80.0 mg)-packed SAIDA reaction tube and filled with a mixed solvent of MCH/2-PrOH (2/1). The mixed solvent was pumped through the reaction tube at a flow rate of 0.5 mL min^{-1} under a maximum of 10 W MW irradiation for 5 min. A solution of **1** in the mixed solvent (0.05 M) was pumped into the reaction tube, and then the solution vessel was sequentially rinsed with the mixed solvents four times (1 mL × 3, then 20 mL × 1) using a pump. The MW irradiation was stopped, and ethyl acetate/toluene (1/1, 40 mL) or dichloromethane (40 mL) was pumped to further wash the entire flow path. The whole reaction mixture and washing solution were collected and concentrated in vacuo, dissolved in deuterated chloroform ($CDCl_3$), and analyzed by ^1H NMR spectroscopy using 1,1,2,2-tetrachloroethane (52.5 µL, 0.5 mmol) as an internal standard.

Condition C (external addition of hydrogen gas using EYELA, MR-2G-100): The entire flow path was fitted with the 5% Pt/CB (80.0 mg)-packed EYELA reaction tube and filled with toluene. Toluene (0.5 mL min^{-1}) and hydrogen gas (80 mL min^{-1}) were pumped through the reaction tube at a flow rate of 0.5 mL min^{-1} at 320 °C under a maximum of 20 W MW irradiation for 5 min. A solution of **3a** in toluene (0.05 M) was pumped into the reaction tube, and then the solution vessel was sequentially rinsed with toluene four times (1 mL × 3, then 20 mL × 1) using a pump. The MW irradiation was stopped, and ethyl acetate/toluene (1/1, 40 mL) was pumped to further wash the entire flow path. The whole reaction mixture and washing solution were collected and concentrated in vacuo, dissolved in deuterated chloroform ($CDCl_3$), and analyzed by ^1H NMR spectroscopy using 1,1,2,2-tetrachloroethane (52.5 µL, 0.5 mmol) as an internal standard.

9. Preparation of substrates

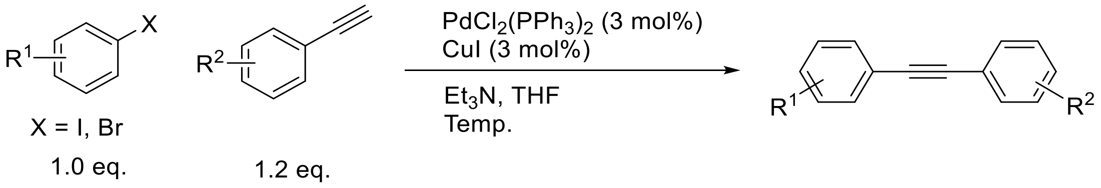

Supplementary Fig. 9 General procedure A

General procedure A (GP A, Supplementary Fig. 9) A solution of aryl halide (1.0 eq.), alkyne (1.2 eq.), $Pd(PPh_3)_2Cl_2$ (3 mol%), and CuI (3 mol%) in a mixture of Et_3N and THF was stirred under argon at a suitable temperature (X = I: room temperature, Br: 80 °C) until complete conversion was detected by TLC analysis. The crude reaction mixture was diluted with ethyl acetate, filtered through a celite pad, concentrated, and purified by silica-gel column chromatography. If necessary, the product was further purified by recrystallization.

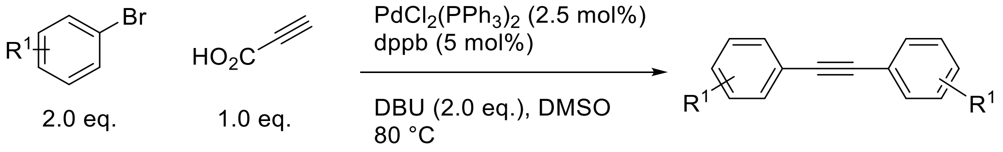

Supplementary Fig. 10 General procedure B

General procedure B (GP B, Supplementary Fig. 10) A solution of aryl bromide (2.0 eq.), Propiolic acid (1.0 eq.), $Pd(PPh_3)_2Cl_2$ (2.5 mol%), dppb (5 mol%), and DBU (2.0 eq.) in a mixture of DMSO was stirred under argon for 24 h at 80 °C. The reaction mixture was cooled at room temperature, quenched with saturated NH_4Cl aqueous solution, extracted with diethyl ether four times, and washed with brine. The combined organic layers were dried over Na_2SO_4, concentrated, and purified by silica-gel column chromatography. If necessary, the product was further purified by recrystallization.

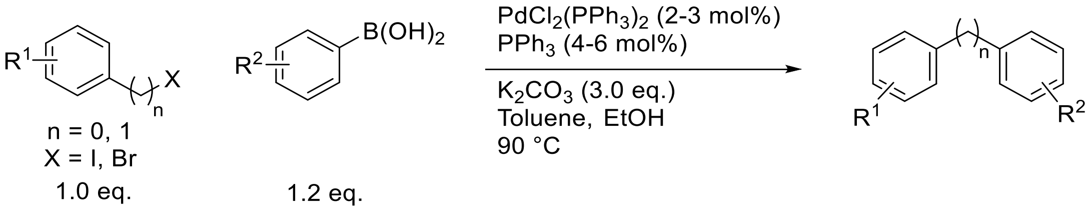

Supplementary Fig. 11 General procedure C

General procedure C (GP C, Supplementary Fig. 11) A solution of aryl halide (n = 0, 1, 1.0 eq.), arylboronic acid (1.2 eq.), $Pd(PPh_3)_2Cl_2$ (2–3 mol%) with PPh_3 (4–6 mol%) and K_2CO_3 (3 eq.) in a mixture of EtOH and toluene was stirred under argon for 24 h at 90 °C in the oil bath until complete conversion was detected by TLC analysis. The reaction mixture was cooled at room temperature, diluted with ethyl acetate, filtered through a celite pad, concentrated, and purified by silica-gel column chromatography.

1-(4-*tert*-Butylphenyl)-2-phenylacetylene (1b)

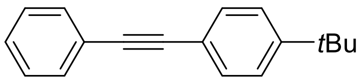

According to **GP A**, iodobenzene (1632.1 mg, 8.0 mmol), 4-(*tert*-Butyl)phenylacetylene (1519.1 mg, 9.6 mmol), $PdCl_2(PPh_3)_2$ (168.3 mg, 0.24 mmol), CuI (45.7 mg, 0.24 mmol), Et_3N (10 mL), and THF (10 mL) were stirred at room temperature. After confirming the disappearance of iodobenzene by TLC, the reaction mixture was diluted with ethyl acetate and filtered through celite. The filtrate was concentrated under reduced pressure, purified by silica-gel column chromatography with hexane, and recrystallized with ethanol to give **1b** (1002.2 mg, 4.3 mmol) in 53% yield as a colorless solid.

^{1}H NMR (500 MHz, $CDCl_3$): δ 7.53 (dd, *J* = 1.8, 7.8 Hz, 2H), 7.47 (d, *J* = 8.0 Hz, 2H), 7.38–7.30 (m, 5H), 1.33 (s, 9H); ^{13}C NMR (125 MHz, $CDCl_3$): δ 151.5, 131.6, 131.3, 128.3, 128.0, 125.3, 123.5, 120.2, 89.5, 88.7, 34.8, 31.2. Spectroscopic data of ^{1}H NMR was identical to that of reference 1.

1-(3-Methylphenyl)-2-phenylacetylene (1c)

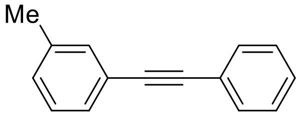

According to **GP A**, 3-iodotoluene (1744.3 mg, 8.0 mmol), ethynylbenzene (817.1 mg, 8.0 mmol), $PdCl_2(PPh_3)_2$ (168.3 mg, 0.24 mmol), CuI (45.7 mg, 0.24 mmol), Et_3N (10 mL), and THF (10 mL) were stirred at room temperature. After confirming the disappearance of 3-iodotoluene by TLC, the reaction mixture was diluted with ethyl acetate and filtered through celite. The filtrate was concentrated under reduced pressure and purified by silica-gel column chromatography with hexane to give **1c** (1264.5 mg, 6.6 mmol) in 82% yield as pale yellow oil.

^{1}H NMR (400 MHz, $CDCl_3$): δ 7.54–7.52 (m, 2H), 7.37–7.32 (m, 5H), 7.24 (dd, *J* = 7.2, 8.0 Hz, 1H), 7.14 (d, *J* = 7.2 Hz, 1H), 2.35 (s, 3H); ^{13}C NMR (100 MHz, $CDCl_3$): δ 138.0, 132.2, 131.6, 129.1, 128.7, 128.3, 128.2, 128.2, 123.3, 123.0, 89.5, 89.0, 21.2. Spectroscopic data of ^{1}H NMR was identical to that of reference 2.

di-*p*-Tolylacetylene (1d)

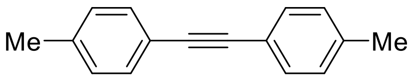

According to **GP A**, 4-iodotoluene (1744.3 mg, 7.0 mmol), 4-ethynyltoluene (975.7 mg, 8.4 mmol), $PdCl_2(PPh_3)_2$ (147.2 mg, 0.21 mmol), CuI (40.0 mg, 0.21 mmol), Et_3N (7.5 mL), and THF (7.5 mL) were stirred for 6 h at room temperature. The reaction mixture was diluted with ethyl acetate and filtered through celite. The filtrate was concentrated under reduced pressure, purified by silica-gel column chromatography (hexane/chloroform = 30/1), and recrystallized with chloroform and methanol to give **1d** (878.5 mg, 4.3 mmol) in 61% yield as a colorless solid.

^{1}H NMR (400 MHz, $CDCl_3$): δ 7.41 (d, *J* = 7.8 Hz, 4H), 7.14 (d, *J* = 7.8 Hz, 4H), 2.36 (s, 6H); ^{13}C NMR (100 MHz, $CDCl_3$): δ 138.1, 131.4, 129.1, 120.3, 88.8, 21.5. Spectroscopic data of ^{1}H NMR was identical to that of reference 3.

di-*m*-Tolylacetylene (1e)

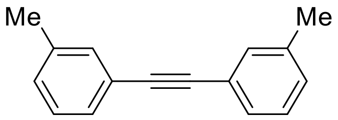

According to **GP A**, 3-iodotoluene (2180.4 mg, 10.0 mmol), 3-ethynyltoluene (1393.9 mg, 12.0 mmol), $PdCl_2(PPh_3)_2$ (210.3 mg, 0.30 mmol), CuI (57.1 mg, 0.30 mmol), Et_3N (10 mL), and THF (10 mL) were stirred at room temperature. After confirming the disappearance of 3-iodotoluene by TLC, the reaction mixture was diluted with ethyl acetate and filtered through celite. The filtrate was concentrated under reduced pressure, purified by silica-gel column chromatography with hexane, and recrystallized with chloroform and methanol to give **1e** (607.1 mg, 2.9 mmol) in 29% yield as a colorless solid.
^{1}H NMR (400 MHz, $CDCl_3$): δ 7.36–7.32 (m, 4H), 7.25 (dd, *J* = 7.2, 8.0 Hz 2H), 7.14 (d, *J* = 7.6 Hz, 2H), 2.36 (s, 6H); ^{13}C NMR (100 MHz, $CDCl_3$): δ 138.0, 132.1, 129.1, 128.6, 128.2, 123.1, 89.2, 21.2. Spectroscopic data of ^{1}H NMR was identical to that of reference 4.

di-*o*-Tolylacetylene (1f)

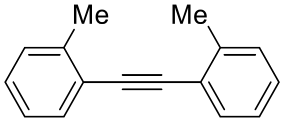

According to **GP B**, 2-bromotoluene (2052.5 mg, 12.0 mmol) propiolic acid (420.3 mg, 6.0 mmol), $PdCl_2(PPh_3)_2$ (210.3 mg, 0.30 mmol), dppb (255.9 mg, 0.60 mmol), DBU (1826.9 mg, 12.0 mmol), DMSO (30 mL) were stirred for 48 h at 80 °C. After cooling the reaction mixture to room temperature, saturated ammonium chloride solution was added, and extracted with diethyl ether four times. The combined organic layers were washed with brine, dried over Na_2SO_4, concentrated in vacuo, and purified by silica-gel column chromatography with hexane to give **1f** (586.1 mg, 2.8 mmol) in 47% yield as pale yellow oil.
^{1}H NMR (400 MHz, $CDCl_3$): δ 7.51 (d, *J* = 7.6 Hz, 2H), 7.24–7.23 (m, 4H), 7.22–7.16 (m, 2H), 2.53 (s, 6H); ^{13}C NMR (100 MHz, $CDCl_3$): δ 139.9, 131.8, 129.5, 128.2, 125.6, 123.3, 92.3, 21.0. Spectroscopic data of ^{1}H NMR was identical to that of reference 4.

Bis(4-*tert*-butylphenyl)acetylene (1g)

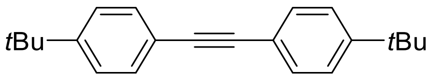

According to **GP B**, 4-*tert*-butylbromobenzene (3409.9 mg, 16.0 mmol) propiolic acid (560.4 mg, 8.0 mmol), $PdCl_2(PPh_3)_2$ (280.4 mg, 0.40 mmol), dppb (341.2 mg, 0.80 mmol), DBU (2435.8 mg, 16.0 mmol), and DMSO (30 mL) were stirred for 48 h at 80 °C. After cooling the reaction mixture to room temperature, saturated ammonium chloride solution was added, and extracted with diethyl ether four times. The combined organic layers were washed with brine, dried over Na_2SO_4, concentrated in vacuo, purified by silica-gel column chromatography with hexane, and recrystallized with chloroform and ethanol to give **1g** (742.3 mg, 2.6 mmol) in 32% yield as a colorless solid.
^{1}H NMR (400 MHz, $CDCl_3$): δ 7.48–7.45 (m, 4H), 7.38–7.34 (m, 4H), 1.32 (s, 18H); ^{13}C NMR (100 MHz, $CDCl_3$): δ 151.3, 131.3, 125.3, 120.4, 88.8, 34.8, 31.2. Spectroscopic data of ^{1}H NMR was identical to that of reference 5.

Bis(2,4-dimethylphenyl)acetylene (1h)

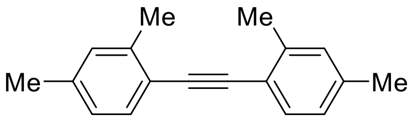

According to **GP B**, 4-Bromo-*m*-xylene (2220.7 mg, 12.0 mmol) propiolic acid (420.3 mg, 6.0 mmol), $PdCl_2(PPh_3)_2$ (210.3 mg, 0.30 mmol), dppb (255.9 mg, 0.60 mmol), DBU (1826.9 mg, 12.0 mmol), and DMSO (30 mL) were stirred for 48 h at 80 °C. After cooling the reaction mixture to room temperature, saturated ammonium chloride solution was added, and extracted with diethyl ether four times. The combined organic layers were washed with brine, dried over Na_2SO_4, concentrated in vacuo, purified by silica-gel column chromatography with hexane, and recrystallized with ethyl acetate and methanol to give **1h** (410.5 mg, 1.8 mmol) in 29% yield as a colorless solid.

Mp 77.0–78.6 °C; IR(ATR) cm^{-1}: 3016, 2918, 2730, 1878, 1732, 1610, 1495, 1445, 1376, 1306, 1281, 1230, 1154, 1126, 1039, 933, 908; ^{1}H NMR (400 MHz, $CDCl_3$): δ 7.39 (d, *J* = 7.6 Hz, 2H), 7.05 (s, 2H), 6.98 (d, *J* = 7.6 Hz, 2H), 2.48 (s, 6H), 2.33 (s, 6H); ^{13}C NMR (125 MHz, $CDCl_3$): δ 139.7, 138.0, 131.6, 130.3, 126.3, 120.4, 91.7, 21.4, 20.8.; HRMS (DART) m/z: $[M+H]^+$; Calcd for $C_{18}H_{19}$: 235.14868, Found 235.14957.

1-(1-Naphthyl)-2-phenylacetylene (1i)

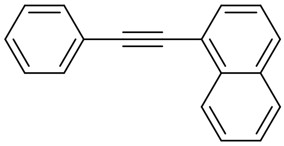

According to **GP A**, 1-iodonaphthalene (1905.5 mg, 7.5 mmol), ethynylbenzene (919.3 mg, 9.0 mmol), $PdCl_2(PPh_3)_2$ (210.3 mg, 0.30 mmol), CuI (28.6 mg, 0.15 mmol), *i*-Pr_2NH (1517.9 mg, 15 mmol), and THF (12 mL) were stirred for 30 min at room temperature. The reaction mixture was diluted with ethyl acetate and filtered through celite. The filtrate was concentrated under reduced pressure and purified by silica-gel column chromatography with hexane to give **1i** (1227.5 mg, 5.4 mmol) in 72% yield as pale yellow oil.

^{1}H NMR (400 MHz, $CDCl_3$): δ 8.44 (d, *J* = 8.4 Hz, 1H), 7.85 (dd, *J* = 8.8, 9.2 Hz, 2H), 7.77 (d, *J* = 7.2 Hz, 1H), 7.65 (dd, *J* = 1.4, 7.8 Hz, 2H), 7.62–7.52 (m, 2H), 7.46 (dd, *J* = 7.6, 8.0 Hz, 1H), 7.42–7.35 (m, 3H); ^{13}C NMR (100 MHz, $CDCl_3$): δ 133.2, 133.2, 131.6, 130.3, 128.7, 128.4, 128.4, 128.3, 126.8, 126.4, 126.2, 125.3, 123.4, 120.9, 94.3, 87.5. Spectroscopic data of ^{1}H NMR was identical to that of reference 6.

1-(2-Naphthyl)-2-phenylacetylene (1j)

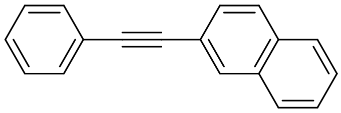

According to **GP A**, 2-bromonaphthalene (1035.4 mg, 5.0 mmol), ethynylbenzene (612.8 mg, 6.0 mmol), $PdCl_2(PPh_3)_2$ (105.2 mg, 0.15 mmol), CuI (28.6 mg, 0.15 mmol), Et_3N (5 mL), and THF (5 mL) were stirred for 12 h at room temperature. The reaction mixture was diluted with ethyl acetate and filtered through celite. The filtrate was concentrated under reduced pressure, purified by silica-gel column chromatography with hexane, and recrystallized with toluene and methanol to give **1j** (246.1 mg, 1.1 mmol) in 22% yield as a colorless solid.

^{1}H NMR (400 MHz, $CDCl_3$): δ 8.07 (s, 1H), 7.83–7.82 (m, 3H), 7.60–7.58 (m, 3H), 7.52–7.49 (m, 2H), 7.39–7.37 (m, 3H);

^{13}C NMR (100 MHz, $CDCl_3$): δ 133.0, 132.7, 131.6, 131.4, 128.4 (2C), 128.3, 128.0, 127.8, 126.6, 126.5, 123.2, 120.5, 89.8, 89.7. Spectroscopic data of ^{1}H NMR was identical to that of reference 7.

1-(3,5-Dimethylphenyl)-2-phenylacetylene (1l)

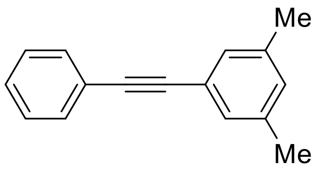

According to **GP A**, 5-iodo-*m*-xylene (1740.5 mg, 7.5 mmol), ethynylbenzene (919.3 mg, 9.0 mmol), $PdCl_2(PPh_3)_2$ (210.3 mg, 0.30 mmol), CuI (28.6 mg, 0.15 mmol), *i*-Pr_2NH (1517.9 mg, 15 mmol), and THF (12 mL) were stirred for 30 min at room temperature. The reaction mixture was diluted with ethyl acetate and filtered through celite. The filtrate was concentrated under reduced pressure, purified by silica-gel column chromatography (hexane/dichloromethane = 50/1), and recrystallized with chloroform and methanol to give **1l** (572.1 mg, 2.8 mmol) in 55% yield as a colorless solid.
^{1}H NMR (400 MHz, $CDCl_3$): δ 7.53–7.50 (m, 2H), 7.37–7.29 (m, 3H), 7.18 (s, 2H), 6.97 (s, 1H), 2.31 (s, 6H); ^{13}C NMR (100 MHz, $CDCl_3$): δ 137.9, 131.6, 130.2, 129.3, 128.3, 128.1, 123.4, 122.8, 89.7, 88.7, 21.1. Spectroscopic data of ^{1}H NMR was identical to that of reference 7.

1-(*o*-Biphenylyl)-2-phenylacetylene (1m)

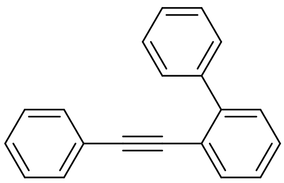

According to **GP A**, 2-iodobiphenyl (1680.7 mg, 6.0 mmol), ethynylbenzene (919.3 mg, 9.0 mmol), $PdCl_2(PPh_3)_2$ (126.2 mg, 0.18 mmol), CuI (34.3 mg, 0.18 mmol), Et_3N (10 mL), and THF (10 mL) were stirred for 1 h at room temperature. The reaction mixture was diluted with ethyl acetate and filtered through celite. The filtrate was concentrated under reduced pressure and purified by silica-gel column chromatography with hexane to give **1m** (1487.3 mg, 5.8 mmol) in 97% yield as a colorless oil.
^{1}H NMR (500 MHz, $CDCl_3$): δ 7.68–7.64 (m, 3H), 7.47–7.27 (m, 11H); ^{13}C NMR (125 MHz, $CDCl_3$): δ 143.9, 140.5, 132.8, 131.3, 129.5, 129.4, 128.5, 128.2, 128.1, 127.9, 127.4, 127.0, 123.4, 121.5, 92.2, 89.3. Spectroscopic data of ^{1}H NMR was identical to that of reference 8.

1,4-Bis(phenylethynyl)benzene (1k)

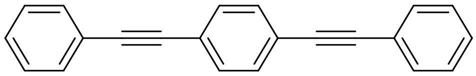

According to **GP A**, 1,4-diiodobenzene (2309.4 mg, 7.0 mmol), ethynylbenzene (1716.0 mg, 16.8 mmol), $PdCl_2(PPh_3)_2$ (73.6 mg, 0.105 mmol), CuI (40.0 mg, 0.21 mmol), Et_3N (7.5 mL), and THF (7.5 mL) were stirred for 18 h at room temperature. The reaction mixture was diluted with ethyl acetate and filtered through celite with toluene and ethyl acetate. The filtrate was concentrated under reduced pressure, purified by silica-gel column chromatography (hexane/chloroform = 50/1), and

recrystallized with chloroform and methanol to give **1k** (1366.9 mg, 4.9 mmol) in 70% yield as a pale yellow solid.
^{1}H NMR (400 MHz, $CDCl_3$): δ 7.56–7.53 (m, 4H), 7.51 (s, 4H), 7.39–7.32 (m, 6H); ^{13}C NMR (100 MHz, $CDCl_3$): δ 131.6, 131.5, 128.5, 128.4, 123.0, 123.0, 91.2, 89.1. Spectroscopic data of ^{1}H NMR was identical to that of reference 9.

1-Benzylnaphthalene (8)

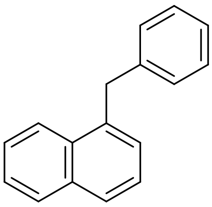

According to **GP C**, benzylbromide (855.2 mg, 5.0 mmol), 1-naphthaleneboronic acid (1031.9 mg, 6.0 mmol), $PdCl_2(PPh_3)_2$ (105.2 mg, 0.15 mmol), PPh_3 (78.7 mg, 0.30 mmol), K_2CO_3 (2073.0 mg, 15.0 mmol), toluene (10 mL), and ethanol (10 mL) were stirred for 12 h at 90 °C. After cooling to room temperature, the reaction mixture was diluted with ethyl acetate and filtered through celite. The filtrate was concentrated under reduced pressure and purified by silica-gel column chromatography (hexane/chloroform = 30/1) to give **8** (240.7 mg, 1.1 mmol) in 22% yield as a colorless solid.
^{1}H NMR (400 MHz, $CDCl_3$): δ 7.99–7.98 (m, 1H), 7.86–7.85 (m, 1H), 7.76 (d, J = 8.4 Hz, 1H), 7.46–7.39 (m, 3H), 7.30–7.24 (m, 3H), 7.20–7.19 (m, 3H), 4.45 (s, 2H); ^{13}C NMR (100 MHz, $CDCl_3$): δ 140.6, 136.6, 133.9, 132.1, 128.7, 128.6, 128.4, 127.3, 127.1, 126.0, 125.9, 125.5, 124.3, 39.0. Spectroscopic data of ^{1}H NMR was identical to that of reference 11.

2-Methylbiphenyl (16)

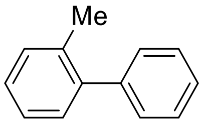

According to **GP C**, 2-iodotoluene (1744.3 mg, 8.0 mmol), phenylboronic acid (1073.0 mg, 8.8 mmol), $PdCl_2(PPh_3)_2$ (112.2 mg, 0.16 mmol), PPh_3 (83.9 mg, 0.32 mmol), K_2CO_3 (3316.8 mg, 24.0 mmol), toluene (10 mL), and ethanol (10 mL) were stirred for 5.5 h at 90 °C. After cooling to room temperature, the reaction mixture was diluted with ethyl acetate and filtered through celite. The filtrate was concentrated under reduced pressure and purified by silica-gel column chromatography (hexane/chloroform = 30/1) to give **16** (932.9 mg, 5.5 mmol) in 69% yield as a colorless oil.
^{1}H NMR (400 MHz, $CDCl_3$): δ 7.44–7.39 (m, 2H), 7.35–7.32 (m, 3H), 7.28–7.23 (m, 4H), 2.28 (s, 3H); ^{13}C NMR (125 MHz, $CDCl_3$): δ 141.9, 135.3, 130.3, 129.8, 129.2, 128.0, 127.2, 126.7, 125.7, 20.5. Spectroscopic data of ^{1}H NMR was identical to that of reference 12.

2,6-Dimethylbiphenyl (17)

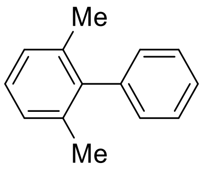

According to **GP C**, 2-iodo-*m*-xylene (1856.5 mg, 8.0 mmol), phenylboronic acid (1170.5 mg, 9.6 mmol), $Pd(PPh_3)_4$ (277.3 mg, 0.24 mmol), K_2CO_3 (3316.8 mg, 24.0 mmol), toluene (10 mL), and ethanol (10 mL) were stirred for 48 h at 90 °C. After

cooling to room temperature, the reaction mixture was diluted with ethyl acetate and filtered through celite. The filtrate was concentrated under reduced pressure and purified by silica-gel column chromatography (hexane/chloroform = 30/1) to give a mixture of **17** and 3.5% biphenyl (1357.5 mg, 7.4 mmol) in 93% yield as a colorless oil.

^{1}H NMR (400 MHz, $CDCl_3$): δ 7.45–7.40 (m, 2H), 7.35–7.31 (m, 1H), 7.18–7.10 (m, 5H), 2.04 (s, 6H); ^{13}C NMR (125 MHz, $CDCl_3$): δ 141.8, 141.0, 136.0, 129.0, 128.4, 127.2, 127.0, 126.6, 20.8. Spectroscopic data of ^{1}H NMR was identical to that of reference 13.

10. Spectroscopic data of products

Phenanthrene (2a)

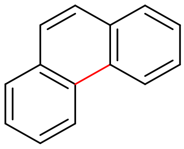

According to **Condition A**, **1a** (89.1 mg, 0.50 mmol) was used. **2a** was obtained in 86% ^{1}H NMR yield. **2a** was isolated as a colorless solid by silica-gel column chromatography with hexane and recrystallized with methanol.

^{1}H NMR (400 MHz, $CDCl_3$): δ 8.70 (d, *J* = 8.0 Hz, 2H), 7.90 (dd, *J* = 0.8, 8.0 Hz, 2H), 7.75 (s, 2H) , 7.68–7.58 (m, 4H); ^{13}C NMR (100 MHz, $CDCl_3$): δ 132.0, 130.3, 128.5, 126.9, 126.5, 122.6. Spectroscopic data of ^{1}H NMR was identical to that of reference 14.

3-*tert*-Butylphenanthrene (2b)

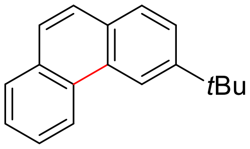

According to **Condition A**, **1b** (58.6 mg, 0.25 mmol) was used. **2b** was obtained in 66% ^{1}H NMR yield. **2b** was isolated as a colorless solid by silica-gel column chromatography with hexane and recrystallized with ethanol and methanol.

^{1}H NMR (400 MHz, $CDCl_3$): δ 8.73 (d, *J* =8.4 Hz, 1H), 8.68 (s, 1H), 7.89–7.87 (m, 1H), 7.84 (d, *J* = 8.4 Hz, 1H), 7.73–7.63 (m, 4H), 7.60–7.56 (m, 1H), 1.50 (s, 9H); ^{13}C NMR (100 MHz, $CDCl_3$): δ 149.3, 132.2, 130.4, 129.9, 129.8, 128.6, 128.2, 126.5, 126.3, 126.3, 126.2, 124.9, 122.5, 118.2, 35.2, 31.5. Spectroscopic data of ^{1}H NMR was identical to that of reference 15.

2-Methylphenanthrene (2c)

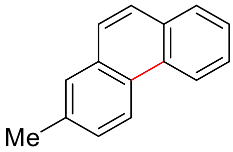

According to **Condition B**, **1c** (96.1 mg, 0.50 mmol) was used. **2c** was obtained in 50% ^{1}H NMR yield. **2c** was isolated as a colorless solid by silica-gel column chromatography with hexane and recrystallized with ethanol and methanol.

^{1}H NMR (500 MHz, $CDCl_3$): δ 8.65 (d, *J* =8.5 Hz, 1H), 8.58 (d, *J* =8.5 Hz, 1H), 7.87 (d, *J* =8.0 Hz, 1H), 7.73–7.66 (m, 3H), 7.67–7.62 (m, 1H), 7.59–7.55 (m, 1H), 7.49 (dd, *J* = 2.0, 8.5 Hz, 1H), 2.57 (s, 3 H); ^{13}C NMR (100 MHz, $CDCl_3$): δ 136.3, 132.1, 131.7, 130.3, 128.5, 128.3, 128.1 (2C), 126.9, 126.6, 126.4, 126.1, 122.5, 122.4, 21.4. Spectroscopic data of ^{1}H NMR was identical to that of reference 16.

3,6-Dimethylphenanthrene (2d)

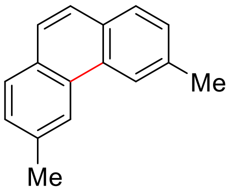

According to **Condition A**, **1d** (103.1 mg, 0.50 mmol) was used. **2d** was obtained in 85% ^{1}H NMR yield. **2d** was isolated as a colorless solid by silica-gel column chromatography (hexane/chloroform = 30/1) and recrystallized with chloroform and methanol.

^{1}H NMR (400 MHz, $CDCl_3$): δ 8.44 (s, 2H), 7.74 (d, *J* = 8.2 Hz, 2H), 7.61 (s, 2H) , 7.39 (d, *J* = 8.2 Hz, 2H), 2.60 (s, 6H); ^{13}C NMR (100 MHz, $CDCl_3$): δ 136.0, 130.1, 130.0, 128.3, 128.1, 125.7, 122.3, 22.1. Spectroscopic data of ^{1}H NMR was identical to that of reference 17.

2,7-Dimethylphenanthrene (2e)

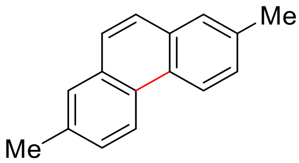

According to **Condition A**, **1e** (103.1 mg, 0.50 mmol) was used. **2e** was obtained in 49% ^{1}H NMR yield. **2e** was isolated as a colorless solid by silica-gel column chromatography (hexane/chloroform = 30/1) and recrystallized with ethanol.

^{1}H NMR (400 MHz, $CDCl_3$): δ 8.53 (d, *J* =8.2 Hz, 2H), 7.65 (s, 2H), 7.64 (s, 2H), 7.46 (dd, *J* = 1.6, 8.2 Hz, 2H), 2.55 (s, 6H); ^{13}C NMR (100 MHz, $CDCl_3$): δ 135.8, 131.8, 128.2, 128.2, 128.0, 126.6, 122.3, 21.4. Spectroscopic data of ^{1}H NMR was identical to that of reference 18.

1,8-Dimethylphenanthrene (2f)

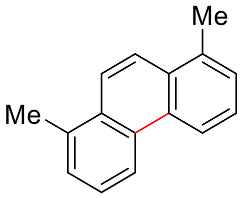

According to **Condition A**, **1f** (103.1 mg, 0.50 mmol) was used. **2f** was obtained in 43% ^{1}H NMR yield. **2f** was isolated as a colorless solid by silica-gel column chromatography (hexane/chloroform = 30/1) and recrystallized with chloroform and methanol.

Mp 191.1–194.7 °C; IR(ATR) cm^{-1}: 3103, 3053, 3019, 2966, 2926, 2865, 2735, 1927, 1858, 1791, 1772, 1722, 1665, 1600, 1522, 1457, 1403, 1379, 1300, 1243, 1229, 1201, 1171, 1135, 1081, 1035, 1016, 970; ^{1}H NMR (400 MHz, $CDCl_3$): δ 8.59 (d, *J* = 8.4 Hz, 2H), 7.99 (s, 2H), 7.54 (dd, *J* = 6.8, 8.4 Hz, 2H), 7.44 (d, *J* = 6.8 Hz, 2H), 2.76 (s, 6H); ^{13}C NMR (100 MHz, $CDCl_3$): δ 134.7, 130.7, 130.4, 127.5, 126.1, 122.6, 121.1, 19.9; HRMS (DART) m/z: $[M+H]^+$; Calcd for $C_{16}H_{15}$: 207.11738, Found 207.11776.

3,6-Di-*tert*-butylphenanthrene (2g)

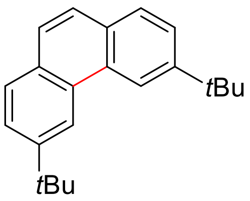

According to **Condition A**, **1g** (145.2 mg, 0.50 mmol) was used. **2g** was obtained in 26% ^{1}H NMR yield. **2g** was isolated as a colorless solid by silica-gel column chromatography with hexane and recrystallized with ethanol.

^{1}H NMR (400 MHz, $CDCl_3$): δ 8.68 (d, J = 2.0 Hz, 2H), 7.82 (d, J = 8.8 Hz, 2H), 7.68–7.66 (m, 4H), 1.51 (s, 18H); ^{13}C NMR (100 MHz, $CDCl_3$): δ 149.0, 130.1, 130.0, 128.2, 125.8, 124.7, 117.9, 35.2, 31.5. Spectroscopic data of ^{1}H NMR was identical to that of reference 19.

1,3,6,8-Tetramethylphenanthrene (2h)

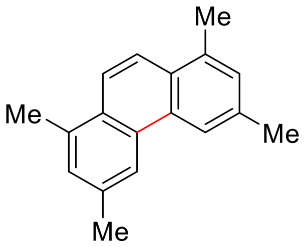

According to **Condition B**, **1h** (117.2 mg, 0.50 mmol) was used. **2h** was obtained in 27% ^{1}H NMR yield. **2h** was isolated as a colorless solid by silica-gel column chromatography (hexane/chloroform = 30/1) and recrystallized with chloroform and methanol.

Mp 149.2–150.6 °C; IR(ATR) cm^{-1}: 2917, 2851, 2728, 1909, 1757, 1723, 1607, 1508, 1458, 1439, 1374, 1297, 1237, 1188, 1033, 963; ^{1}H NMR (500 MHz, $CDCl_3$): δ 8.36 (s, 2H), 7.86 (s, 2H), 7.26 (s, 2H), 2.71 (s, 6H), 2.57 (s, 6H); ^{13}C NMR (125 MHz, $CDCl_3$): δ 135.4, 134.4, 130.5, 129.3, 128.5, 121.5, 120.8, 22.1, 19.8; HRMS (DART) m/z: $[M+H]^+$; Calcd for $C_{19}H_{19}$: 235.14868, Found 235.15156.

Chrysene (2i)

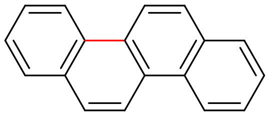

According to **Condition B**, **1i** (114.1 mg, 0.50 mmol) was used. **2i** was obtained in 24% ^{1}H NMR yield. **2i** was isolated as a colorless solid by silica-gel column chromatography (hexane/chloroform = 30/1) and recrystallized with chloroform and methanol.

^{1}H NMR (400 MHz, $CDCl_3$): δ 8.79 (d, J = 8.4 Hz, 2H), 8.73 (d, J = 8.4 Hz, 2H), 8.03–7.99 (m 4H), 7.72 (dd, J = 7.2, 7.4 Hz, 2H), 7.64 (dd, J = 7.2, 7.4 Hz, 2H); ^{13}C NMR (125 MHz, $CDCl_3$): δ 132.2, 130.5, 128.5, 128.2, 127.3, 126.7, 126.4, 123.1, 121.2. Spectroscopic data of ^{1}H NMR was identical to that of reference 15.

Benzo[*a*]anthracene (2j)

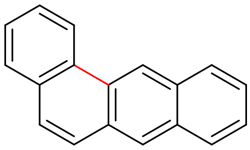

According to **Condition A**, **1j** (114.1 mg, 0.50 mmol) was used. **2j** was obtained in 8% ^{1}H NMR yield. **2j** was isolated as a colorless solid by gel permeation chromatography.

^{1}H NMR (400 MHz, $CDCl_3$): δ 9.17 (s, 1H), 8.84 (d, *J* = 8.4 Hz, 1H), 8.37 (s, 1H), 8.15–8.12 (m 1H), 8.06–8.03 (m 1H), 7.85 (dd, *J* = 1.0, 7.4 Hz, 1H), 7.80 (d, *J* = 8.8 Hz, 1H), 7.71–7.67 (m 1H), 7.65–7.60 (m 2H), 7.58–7.53 (m 2H); ^{13}C NMR (100 MHz, $CDCl_3$): δ 131.9, 131.9, 130.6, 130.5, 128.8, 128.6, 128.4, 127.7, 127.3, 127.1, 126.8, 126.8, 125.8, 125.7, 122.9, 121.5. Spectroscopic data of ^{1}H NMR was identical to that of reference 20.

Dibenzo[*a*,*h*]anthracene (2k)

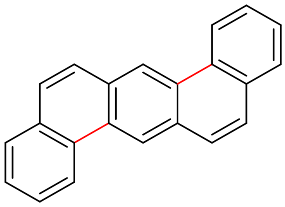

According to **Condition A**, **1k** (69.6 mg, 0.25 mmol) was used. **2k** was obtained in 12% ^{1}H NMR yield. **2k** was isolated as a colorless solid by gel permeation chromatography.

^{1}H NMR (400 MHz, $CDCl_3$): δ 9.17 (s, 2H), 8.89 (d, *J* = 8.4 Hz, 2H), 7.97 (d, *J* = 9.2 Hz, 2H), 7.92 (d, *J* = 7.2 Hz, 2H), 7.78–7.71 (m 4H), 7.67–7.64 (m 2H); ^{13}C NMR (100 MHz, $CDCl_3$): δ 132.0, 130.8, 130.2, 129.1, 128.7, 127.5, 127.2, 127.0, 126.8, 122.9, 122.2. Spectroscopic data of ^{1}H NMR was identical to that of reference 23.

9*H*-Fluorene (7)

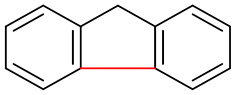

According to **Condition B**, **6** (84.1 mg, 0.50 mmol) was used. **7** was obtained in 54% ^{1}H NMR yield. **7** was isolated as a pale yellow solid by silica-gel column chromatography with hexane and recrystallized with methanol.

According to **Condition A**, **6** (84.1 mg, 0.50 mmol) was used. **7** was obtained in 77% ^{1}H NMR yield. **7** was isolated as a pale yellow solid by silica-gel column chromatography with hexane and recrystallized with methanol.

According to **Condition B**, **16** (84.1 mg, 0.50 mmol) was used. **7** was obtained in 55% ^{1}H NMR yield. **7** was isolated as a pale yellow solid by silica-gel column chromatography with hexane and recrystallized with methanol.

^{1}H NMR (400 MHz, $CDCl_3$): δ 7.79 (d, *J* = 7.2 Hz, 2H), 7.54 (d, *J* = 7.2 Hz, 2H), 7.37 (dd, *J* = 7.2, 7.8 Hz, 2H), 7.30 (dd, *J* = 7.2, 7.8 Hz, 2H), 3.90 (s, 2H); ^{13}C NMR (100 MHz, $CDCl_3$): δ 143.2, 141.7, 126.7, 126.7, 125.0, 119.8, 36.9. Spectroscopic data of ^{1}H NMR was identical to that of reference 24.

11*H*-Benzo[*a*]fluorene (9)

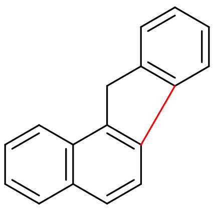

According to **Condition A**, **8** (109.2 mg, 0.50 mmol) was used. **9** was obtained in 23% ^{1}H NMR yield. **9** was isolated as a yellow-red solid by silica-gel column chromatography (hexane/chloroform = 30/1) and recrystallized with chloroform and methanol.

^{1}H NMR (400 MHz, $CDCl_3$): δ 8.02 (d, *J* = 8.4 Hz, 1H), 7.93–7.83 (m, 4H), 7.63 (d, *J* = 8.0 Hz, 1H), 7.57–7.52 (m, 1H), 7.49–7.39 (m, 2H), 7.35–7.31 (m, 1H), 4.19 (s, 2H); ^{13}C NMR (100 MHz, $CDCl_3$): δ 143.3, 142.6, 139.8, 138.9, 132.8, 130.7, 128.9, 127.8, 126.8, 126.4, 126.3, 125.3, 124.9, 124.1, 119.6, 118.7, 35.6. Spectroscopic data of ^{1}H NMR was identical to that of reference 25.

9-Phenyl-9*H*-fluorene (11)

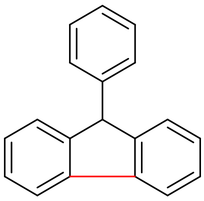

According to **Condition B**, **10** (122.2 mg, 0.50 mmol) was used. **11** was obtained in 23% ^{1}H NMR yield. **11** was isolated as a colorless solid (or pale yellow solid) by silica-gel column chromatography (hexane/dichloromethane = 50/1).

^{1}H NMR (400 MHz, $CDCl_3$): δ 7.80 (d, *J* = 7.2 Hz, 2H), 7.38 (dd, *J* = 7.2, 7.2 Hz, 2H), 7.31 (d, *J* = 7.2 Hz, 2H), 7.28–7.20 (m, 5H), 7.10–7.08 (m, 2H), 5.04 (s, 1H); ^{13}C NMR (100 MHz, $CDCl_3$): δ 147.8, 141.5, 140.9, 128.7, 128.3, 127.3, 126.8, 125.3, 119.8, 54.4. Spectroscopic data of ^{1}H NMR was identical to that of reference 26.

Fluoranthene (13)

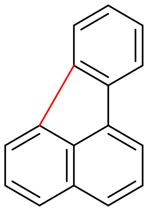

According to **Condition A**, **12** (102.1 mg, 0.50 mmol) was used. **13** was obtained in 42% ^{1}H NMR yield. **13** was isolated as a pale yellow solid by silica-gel column chromatography (hexane/chloroform = 30/1) and recrystallized with chloroform and methanol.

^{1}H NMR (400 MHz, $CDCl_3$): δ 7.96–7.90 (m, 4H), 7.85 (d, *J* = 8.4 Hz, 2H), 7.64 (dd, *J* = 6.8, 8.4 Hz, 2H) 7.41–7.37 (m, 2H); ^{13}C NMR (100 MHz, $CDCl_3$): δ 139.4, 136.9, 132.3, 129.9, 127.9, 127.5, 126.6, 121.5, 120.0. Spectroscopic data of ^{1}H NMR was identical to that of reference 27.

Triphenylene (15)

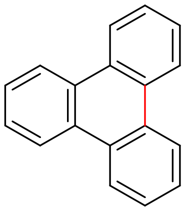

According to **Condition B**, **14** (115.2 mg, 0.50 mmol) was used. **15** was obtained in 19% ^{1}H NMR yield. **15** was isolated as a colorless solid by silica-gel column chromatography (hexane/chloroform = 30/1) and recrystallized with chloroform.

^{1}H NMR (400 MHz, $CDCl_3$): δ 8.64 (dd, *J* = 3.4, 6.0 Hz, 6H), 7.64 (dd, *J* = 3.4, 6.0 Hz, 6H); ^{13}C NMR (100 MHz, $CDCl_3$): δ 129.7, 127.2, 123.3. Spectroscopic data of ^{1}H NMR was identical to that of reference 28.

4-Methyl-9*H*-fluorene (18)

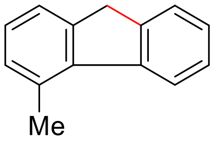

According to **Condition B**, **17** (115.2 mg, 0.50 mmol) was used. **18** was obtained in 57% ^{1}H NMR yield. **18** was isolated as a pale yellow solid by silica-gel column chromatography (hexane/chloroform = 30/1) and recrystallized with ethanol.

^{1}H NMR (400 MHz, $CDCl_3$): δ 7.93 (d, *J* = 7.2 Hz, 1H), 7.57 (d, *J* = 7.6 Hz, 1H), 7.41–7.37 (m, 2H), 7.33–7.29 (m, 1H), 7.21 (dd, *J* = 7.2, 8.0 Hz, 1H), 7.15 (d, *J* = 7.2 Hz, 1H), 3.91 (s, 2H), 2.74 (s, 3H); ^{13}C NMR (100 MHz, $CDCl_3$): δ 143.6, 143.6, 142.6, 139.7, 133.0, 128.9, 126.6, 126.4, 126.0, 124.9, 123.1, 122.4, 37.1, 21.1. Spectroscopic data of ^{1}H NMR was identical to that of reference 29.

2-Methyl-4*H*-cyclopenta[*d*,*e*,*f*]phenanthrene (2l)

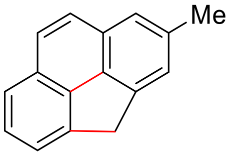

According to **Condition B**, **1l** (103.1 mg, 0.50 mmol) was used. **2l** was obtained in 12% ^{1}H NMR yield. **2l** was isolated as a pale yellow solid by gel permeation chromatography.

^{1}H NMR (500 MHz, $CDCl_3$): δ 7.81–7.73 (m 3H), 7.67 (d, *J* = 7.0 Hz, 1H), 7.62 (s, 1H), 7.60 (dd, *J* = 7.5, 7.5 Hz, 1H), 7.54 (s, 1H), 4.31 (s, 2H), 2.66 (s, 3H); ^{13}C NMR (100 MHz, $CDCl_3$): δ141.8, 141.7, 138.3, 137.3, 136.5, 127.7, 127.5, 126.7, 125.2, 125.0, 123.0, 122.5, 122.2, 121.1, 37.3, 29.7, 22.8. Spectroscopic data of ^{1}H NMR was identical to that of reference 21.

Benzo[*b*]fluoranthene (2m)

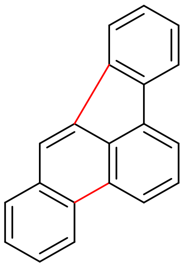

According to **Condition B**, **1m** (127.2 mg, 0.50 mmol) was used. **2m** was obtained in 9% ^{1}H NMR yield. **2m** was isolated as a colorless solid by gel permeation chromatography.

^{1}H NMR (400 MHz, $CDCl_3$): δ 8.68 (d, *J* = 8.4 Hz, 1H), 8.48 (d, *J* = 8.0 Hz, 1H), 8.25 (s, 1H), 8.07 (dd, *J* = 1.4, 7.8 Hz, 1H), 8.04–7.93 (m 3H), 7.78 (dd, *J* = 6.8, 7.2 Hz, 1H), 7.72–7.63 (m 2H), 7.46–7.41 (m 2H); ^{13}C NMR (100 MHz, $CDCl_3$): δ 140.6, 138.5, 137.0, 135.0, 134.0, 132.1, 130.7, 130.2, 128.2, 128.1, 127.5, 127.4, 127.0, 126.8, 123.1, 121.9, 121.6, 121.5, 121.4, 119.6. Spectroscopic data of ^{1}H NMR was identical to that of reference 22.

^{1}H and ^{13}C NMR spectra of products

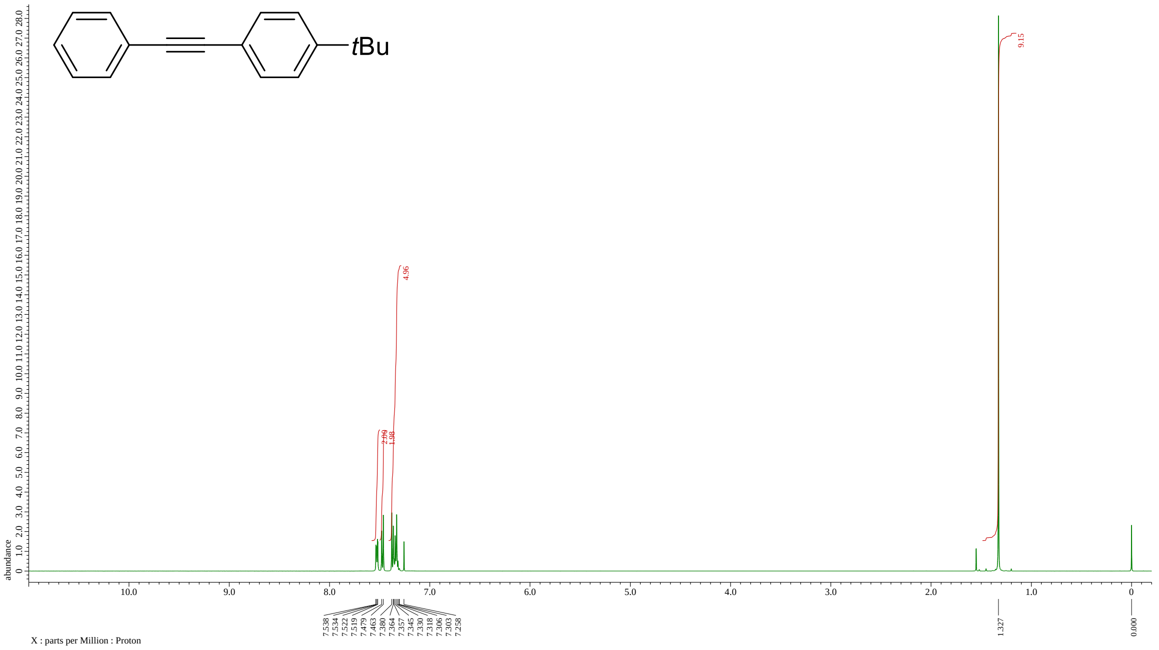

Supplementary Fig. 12 ^{1}H NMR (500 MHz, $CDCl_3$) of 1-(4-*tert*-butylphenyl)-2-phenylacetylene (1b)

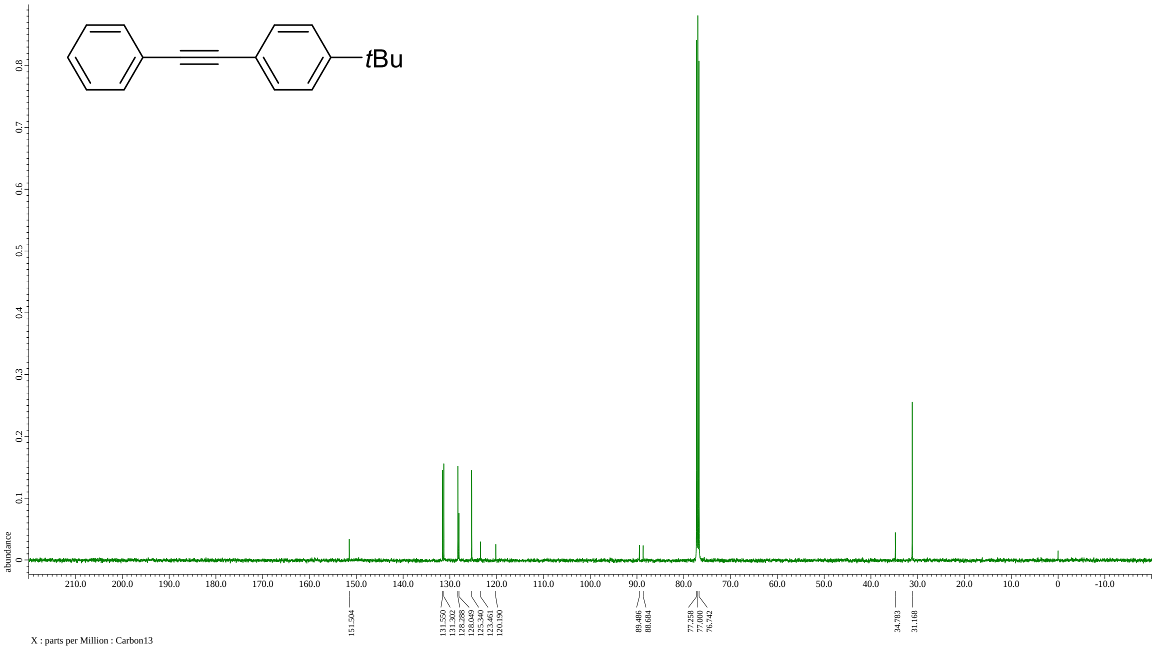

Supplementary Fig. 13 ^{13}C NMR (125 MHz, $CDCl_3$) of 1-(4-*tert*-butylphenyl)-2-phenylacetylene (1b)

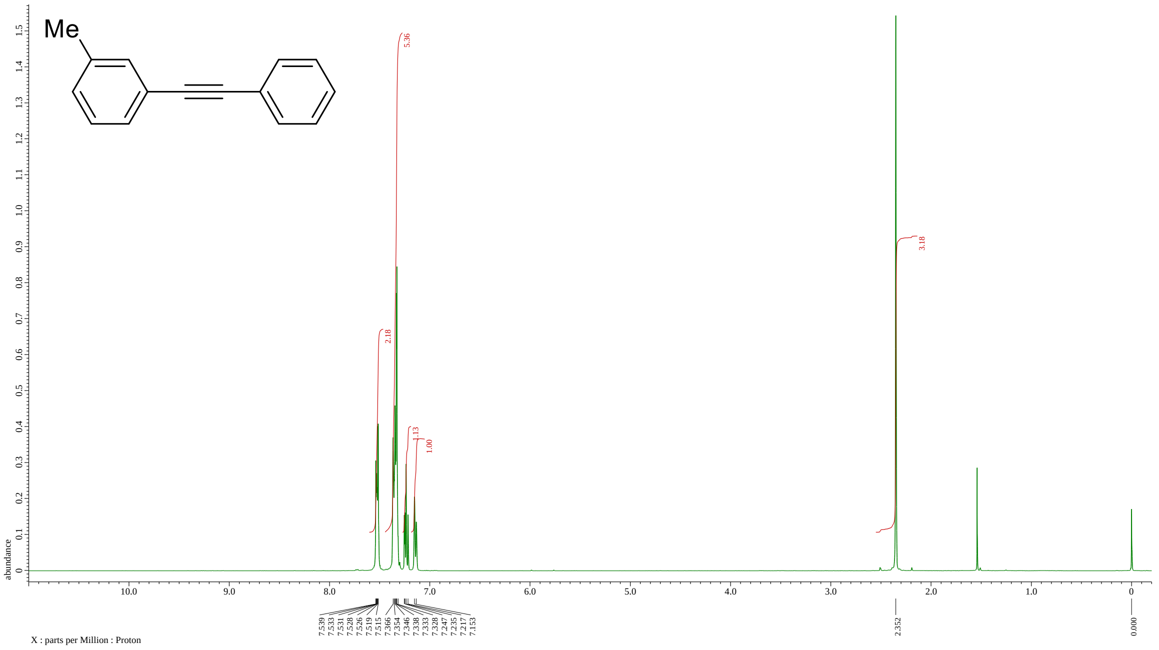

Supplementary Fig. 14 ^{1}H NMR (400 MHz, $CDCl_3$) of 1-(3-methylphenyl)-2-phenylacetylene (1c)

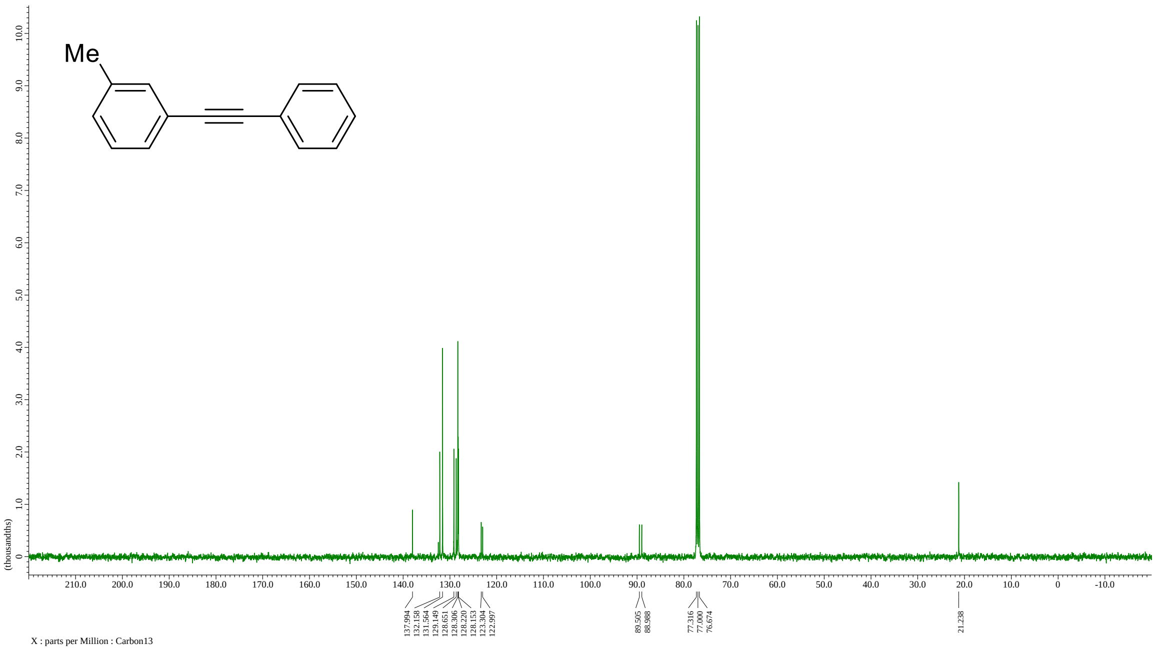

Supplementary Fig. 15 ^{13}C NMR (100 MHz, $CDCl_3$) of 1-(3-methylphenyl)-2-phenylacetylene (1c)

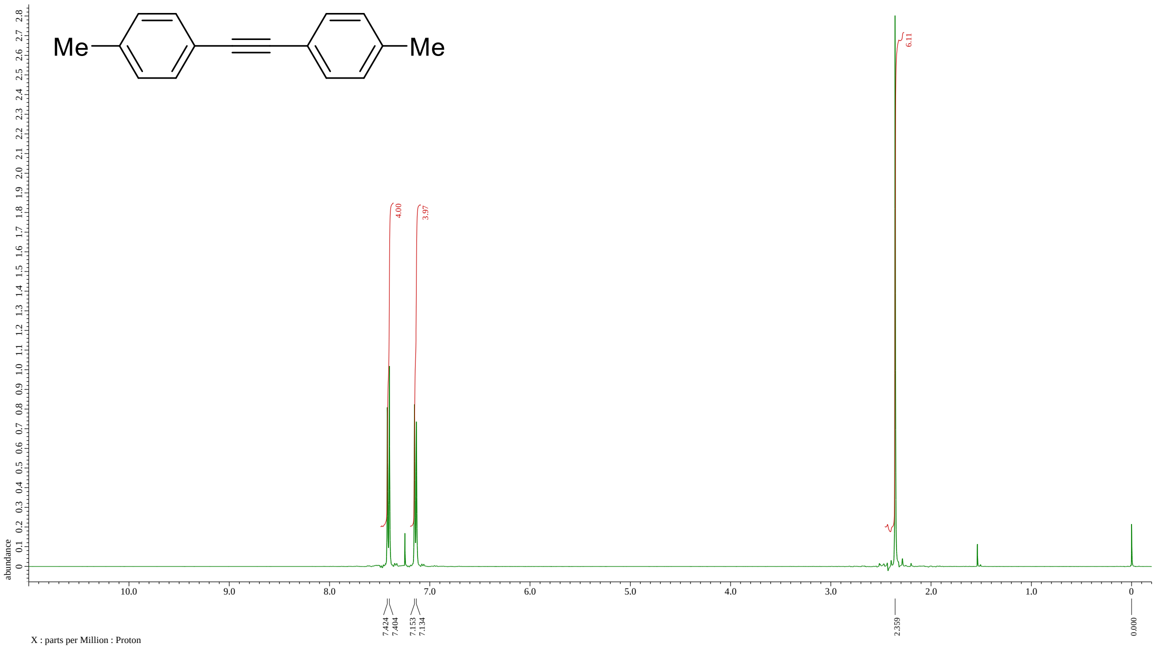

Supplementary Fig. 16 ^{1}H NMR (400 MHz, $CDCl_3$) of di-*p*-tolylacetylene (1d)

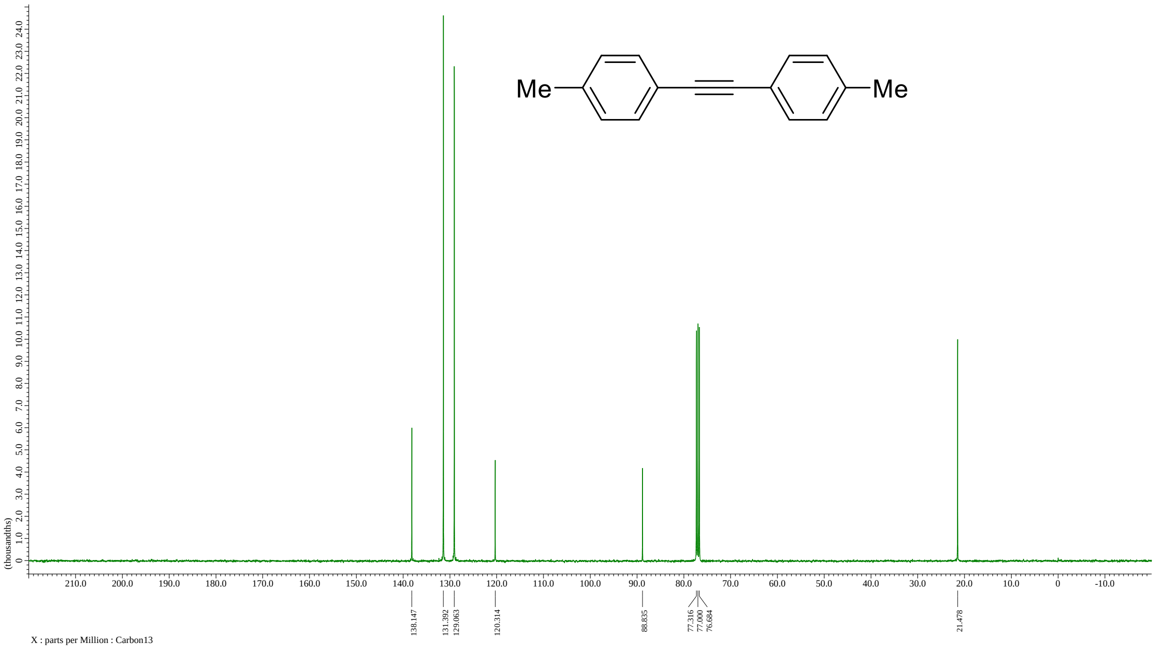

Supplementary Fig. 17 ^{13}C NMR (100 MHz, $CDCl_3$) of di-*p*-tolylacetylene (1d)

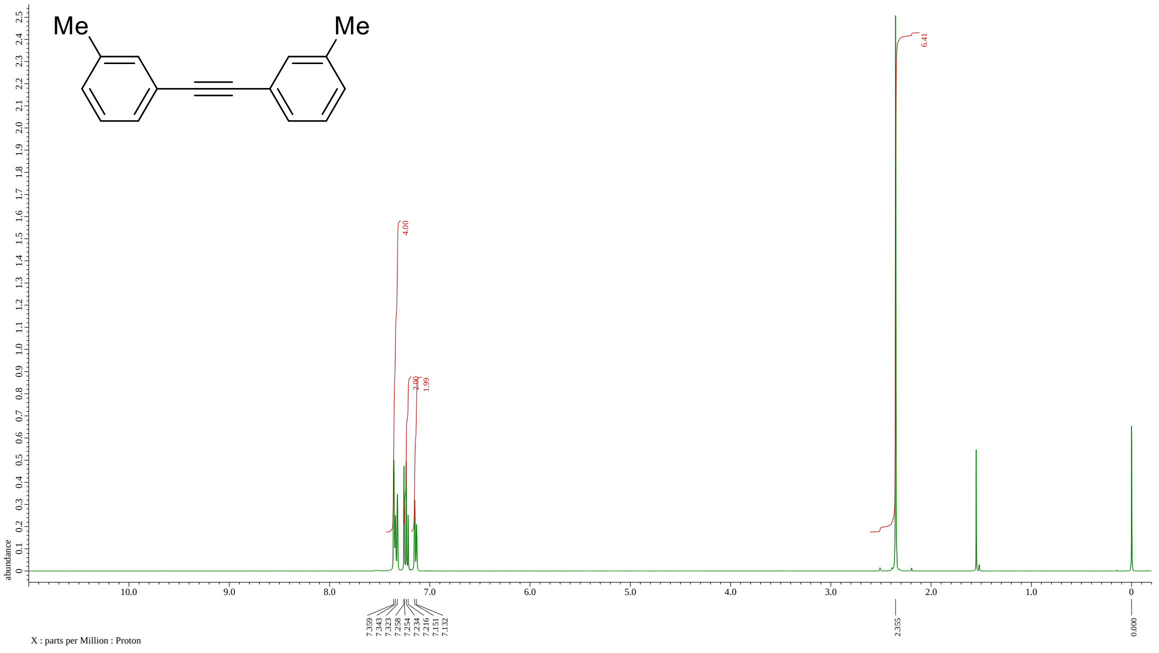

Supplementary Fig. 18 ^{1}H NMR (400 MHz, $CDCl_3$) of di-*m*-tolylacetylene (1e)

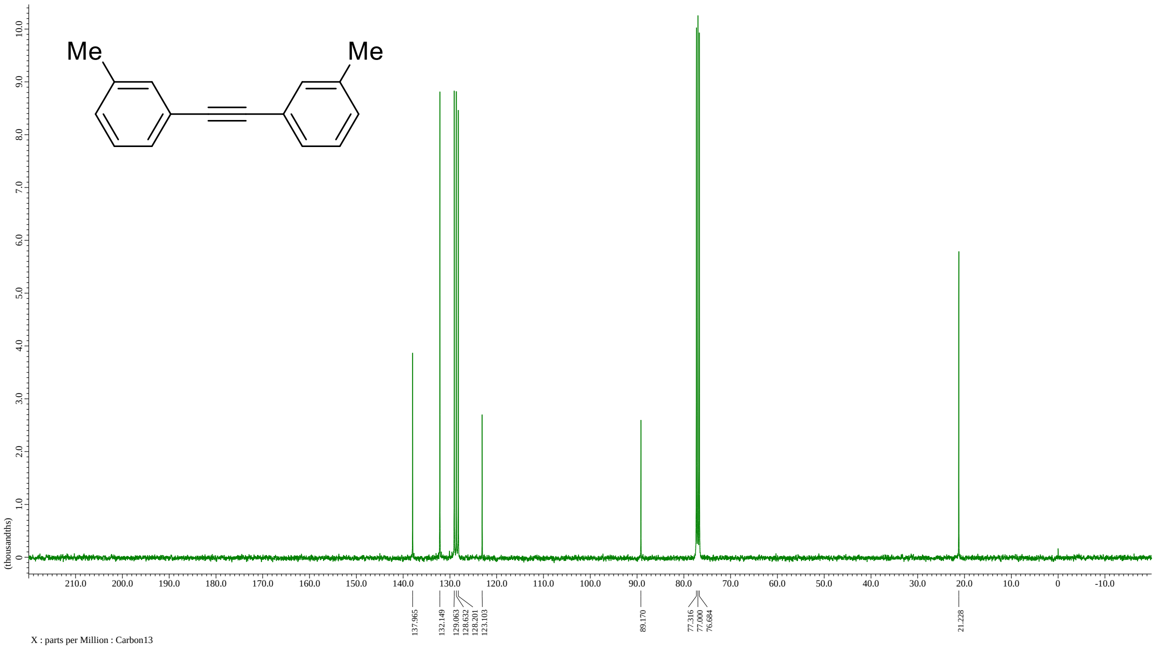

Supplementary Fig. 19 ^{13}C NMR (100 MHz, $CDCl_3$) of di-*m*-tolylacetylene (1e)

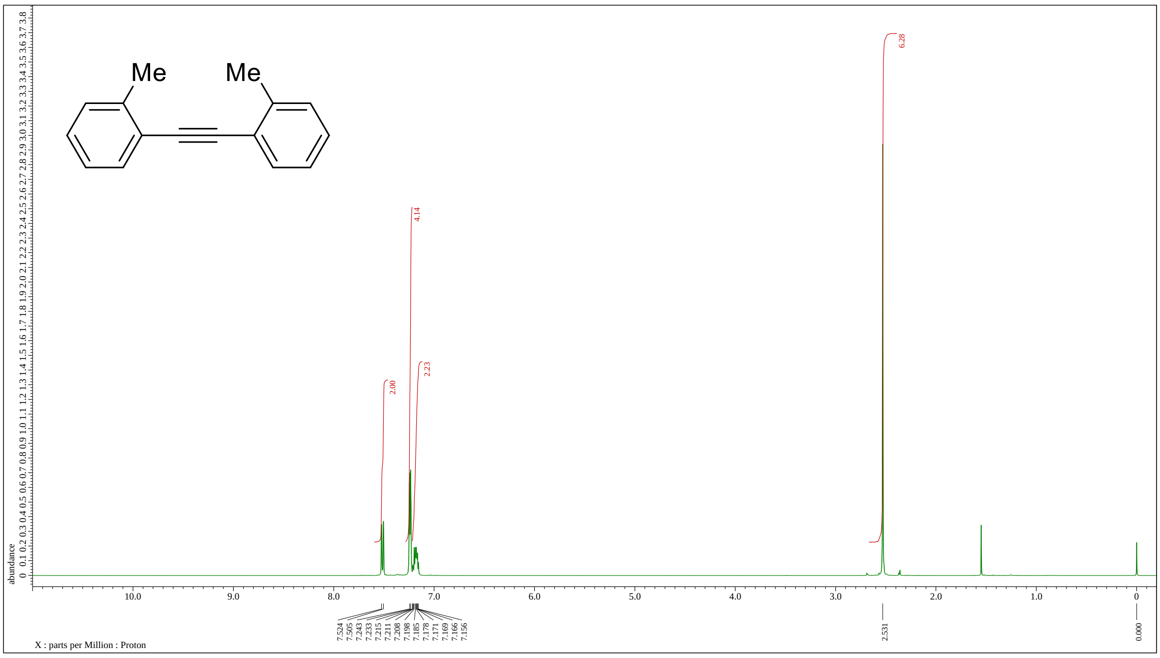

Supplementary Fig. 20 ^{1}H NMR (400 MHz, $CDCl_3$) of di-*o*-tolylacetylene (1f)

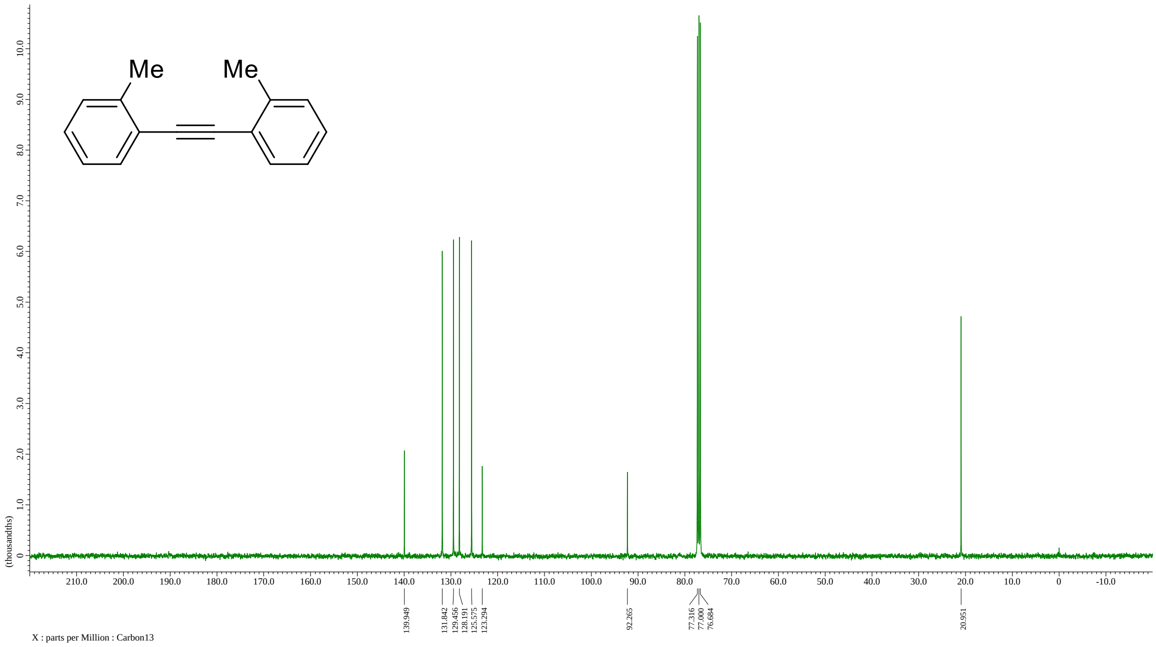

Supplementary Fig. 21 ^{13}C NMR (100 MHz, $CDCl_3$) of di-*o*-tolylacetylene (1f)

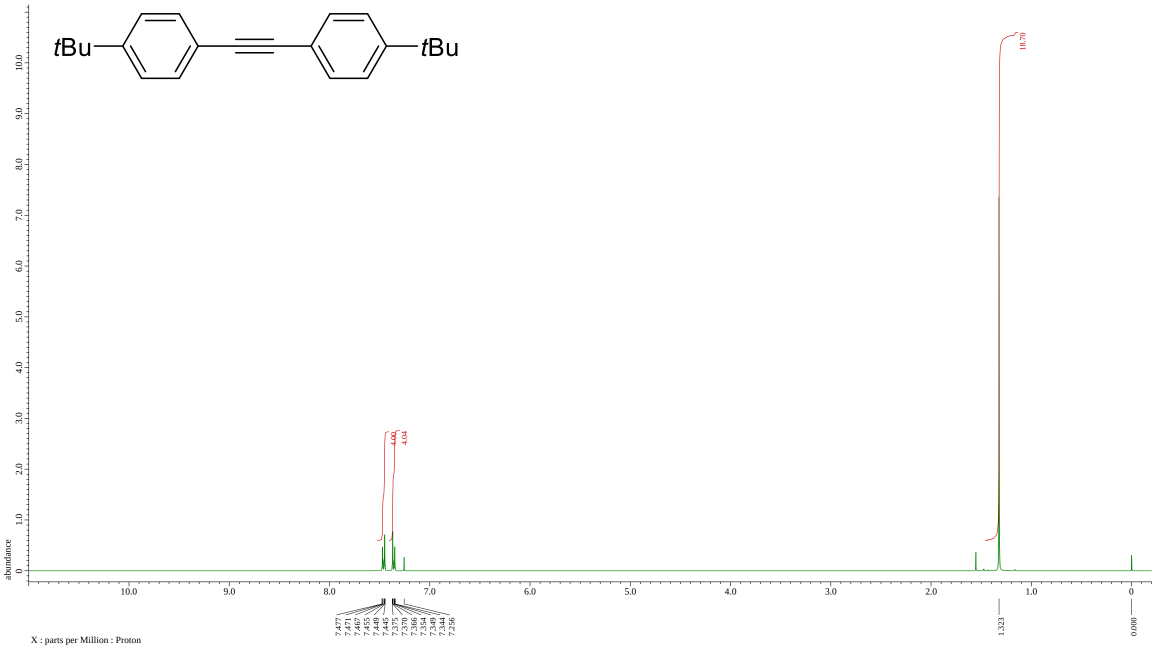

Supplementary Fig. 22 ^{1}H NMR (400 MHz, $CDCl_3$) of bis(4-*tert*-butylphenyl)acetylene (1g)

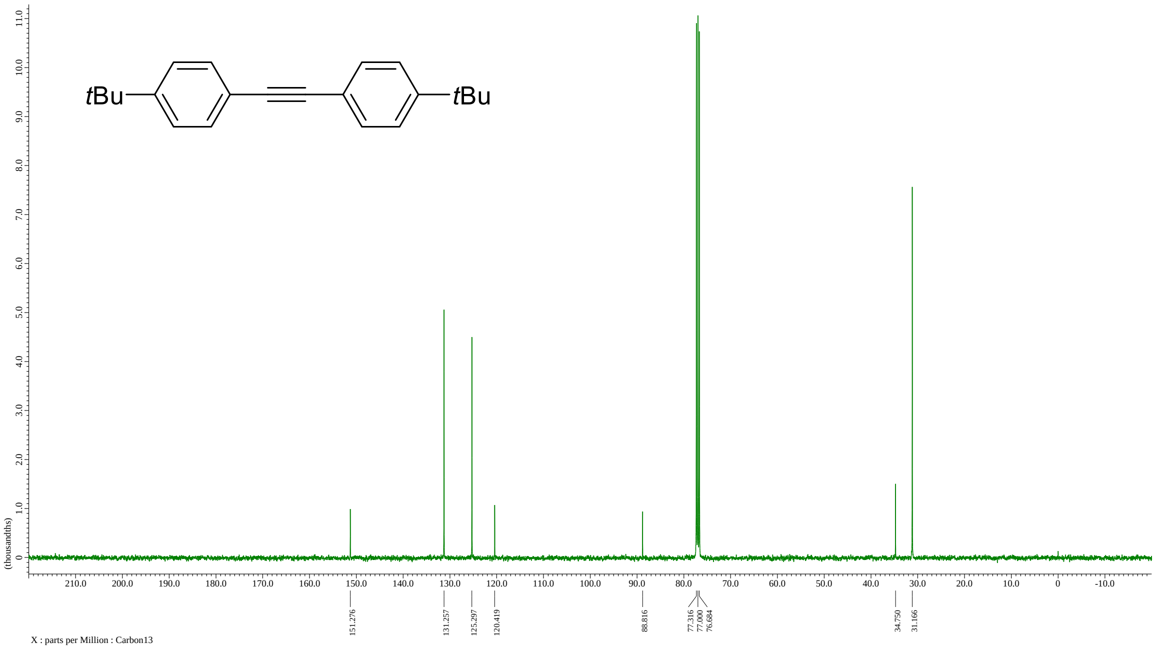

Supplementary Fig. 23 ^{13}C NMR (100 MHz, $CDCl_3$) of bis(4-*tert*-butylphenyl)acetylene (1g)

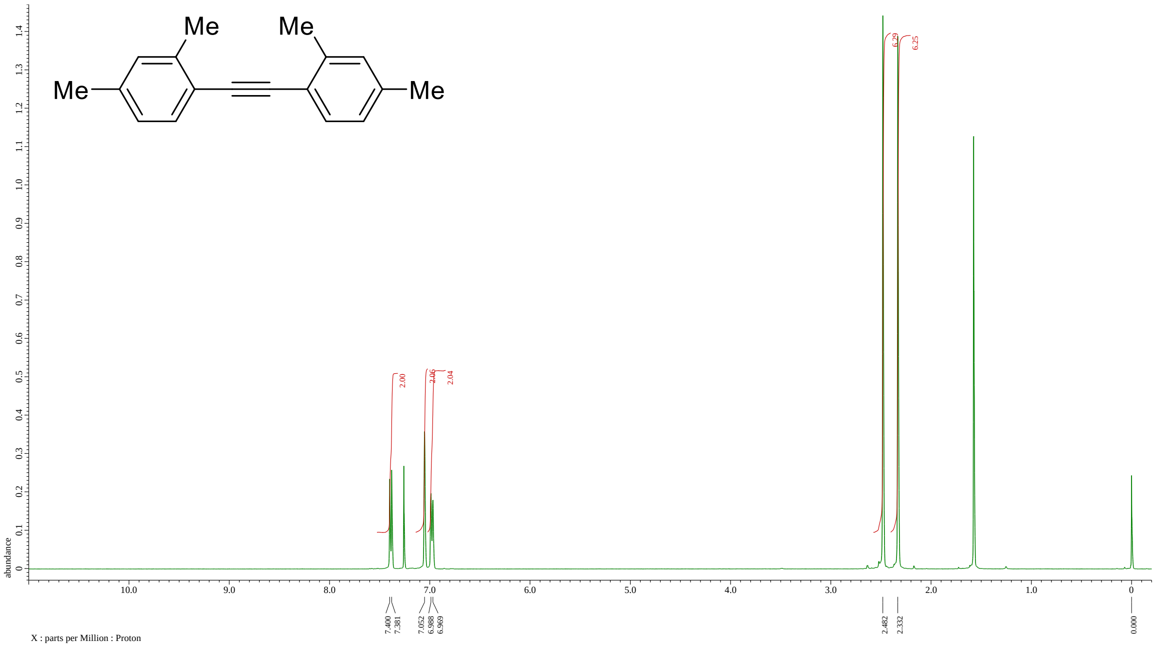

Supplementary Fig. 24 ^{1}H NMR (400 MHz, $CDCl_3$) of bis(2,4-dimethylphenyl)acetylene (1h)

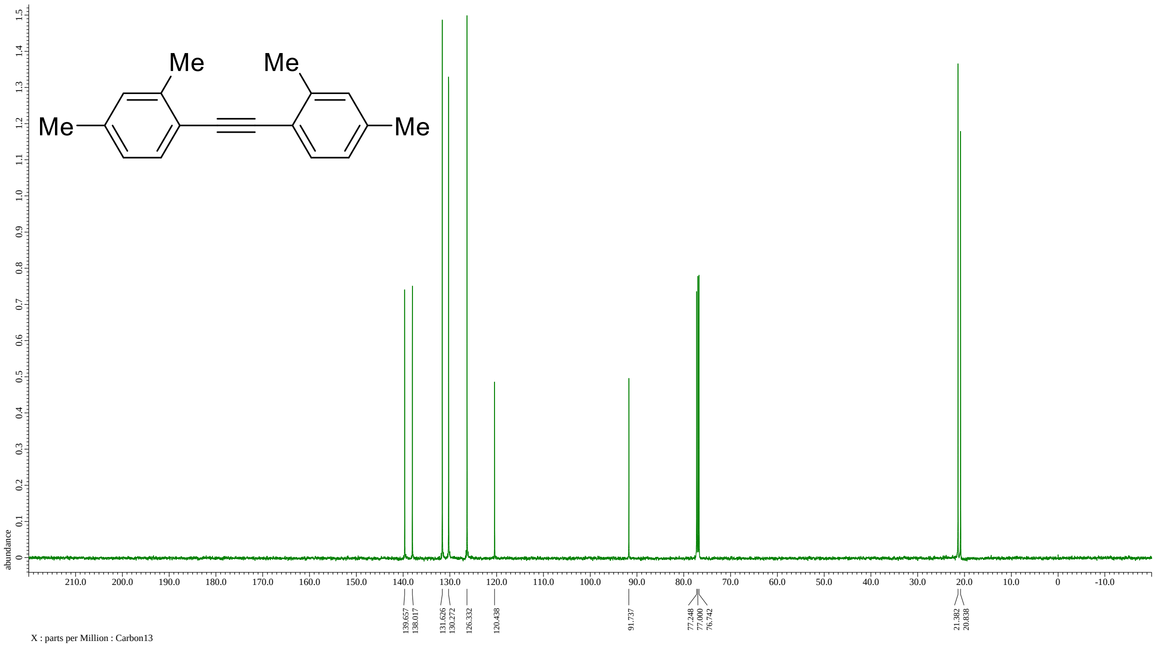

Supplementary Fig. 25 ^{13}C NMR (125 MHz, $CDCl_3$) of bis(2,4-dimethylphenyl)acetylene (1h)

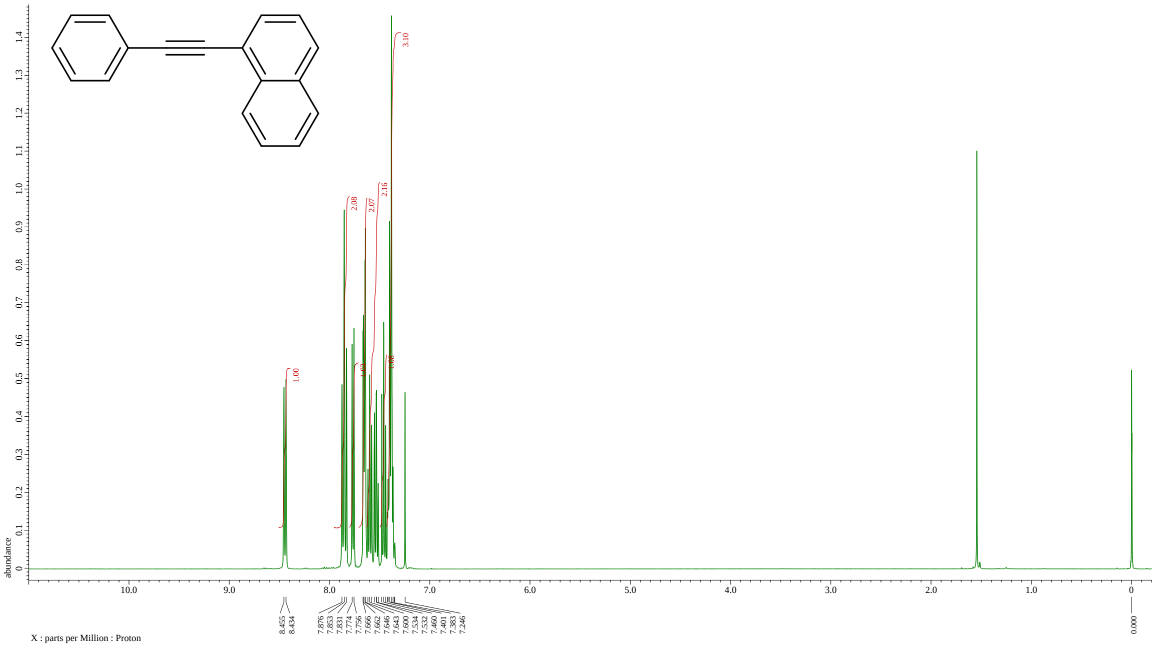

Supplementary Fig. 26 ^{1}H NMR (400 MHz, $CDCl_3$) of 1-(1-naphthyl)-2-phenylacetylene (1i)

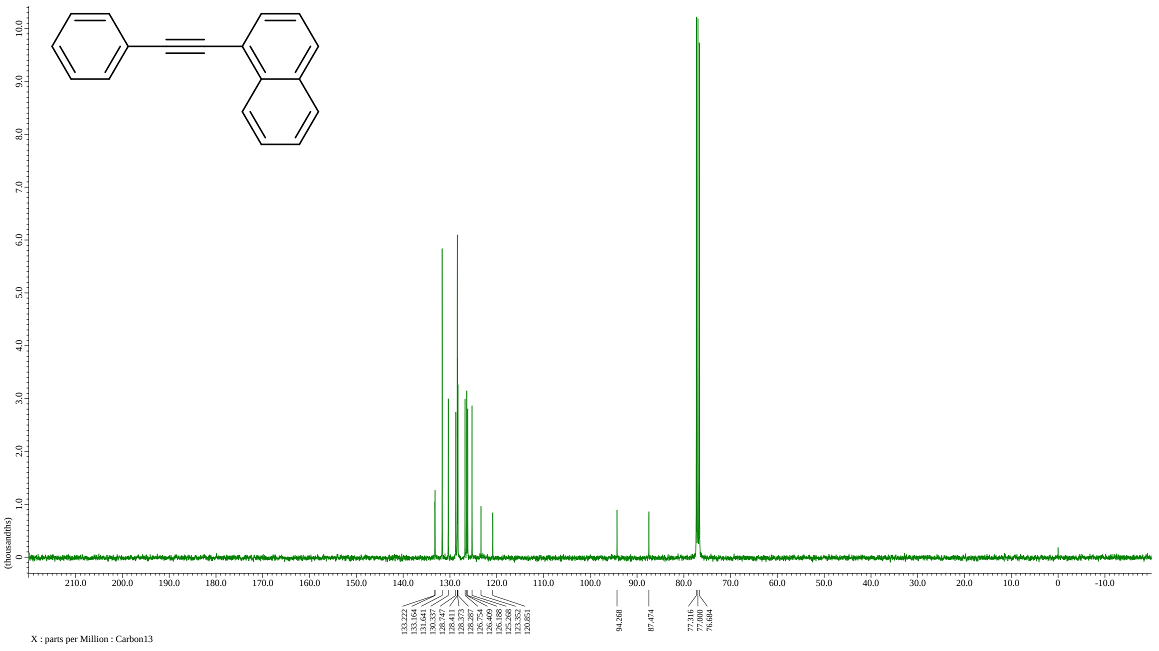

Supplementary Fig. 27 ^{13}C NMR (100 MHz, $CDCl_3$) of 1-(1-naphthyl)-2-phenylacetylene (1i)

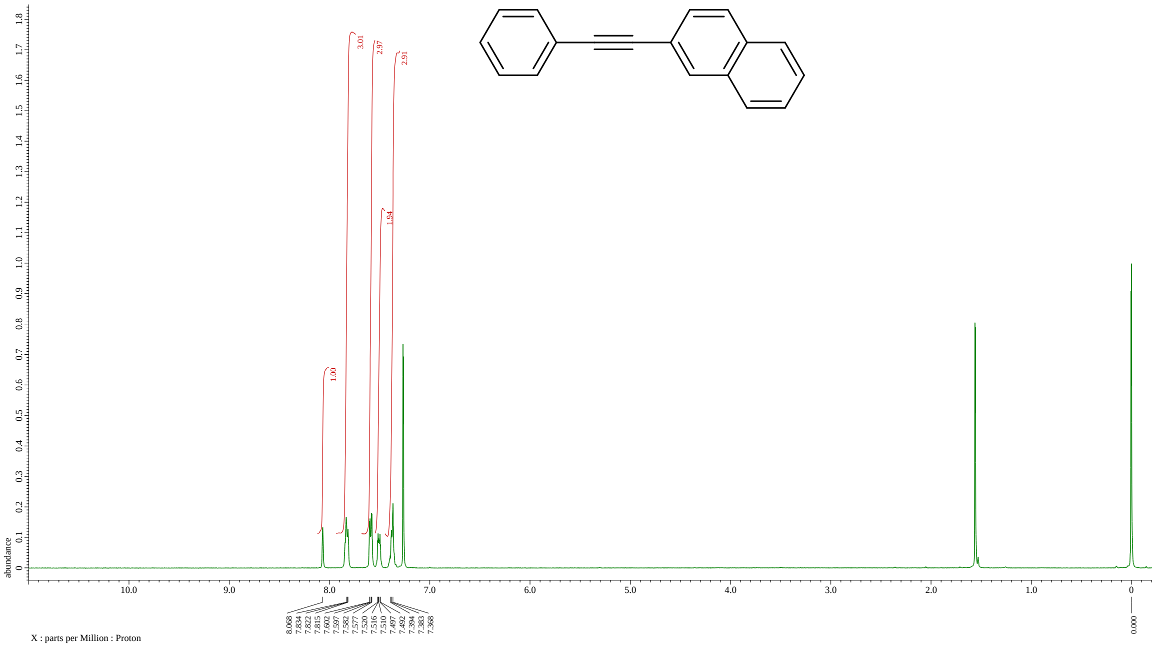

Supplementary Fig. 28 ^{1}H NMR (400 MHz, $CDCl_3$) of 1-(2-naphthyl)-2-phenylacetylene (1j)

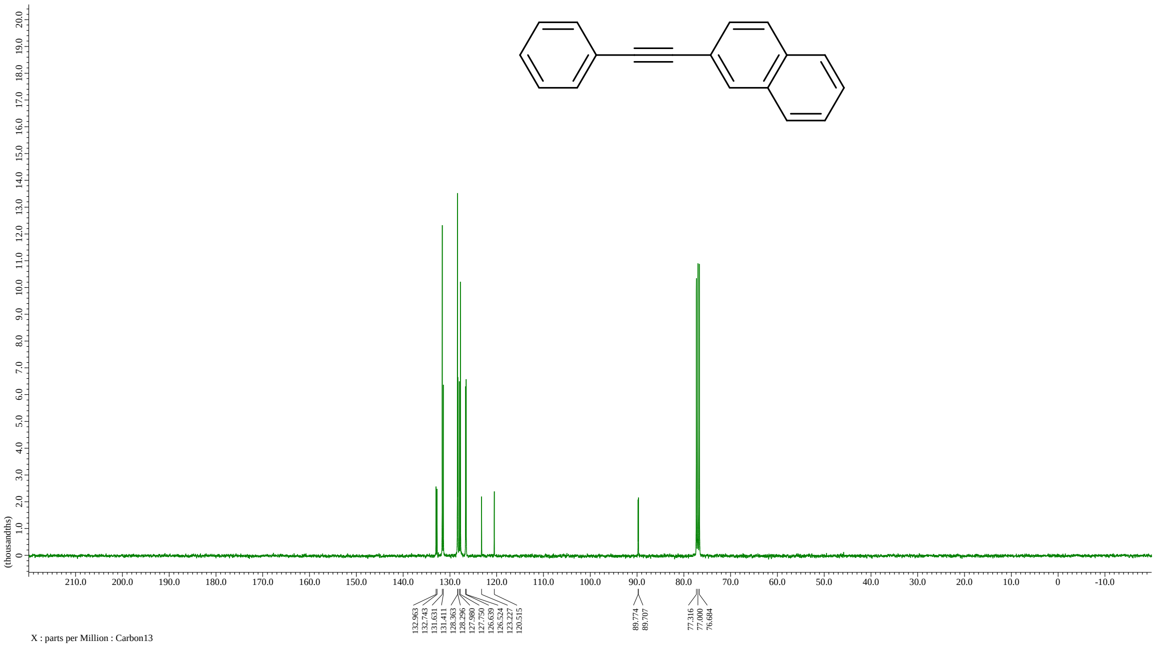

Supplementary Fig. 29 ^{13}C NMR (100 MHz, $CDCl_3$) of 1-(2-naphthyl)-2-phenylacetylene (1j)

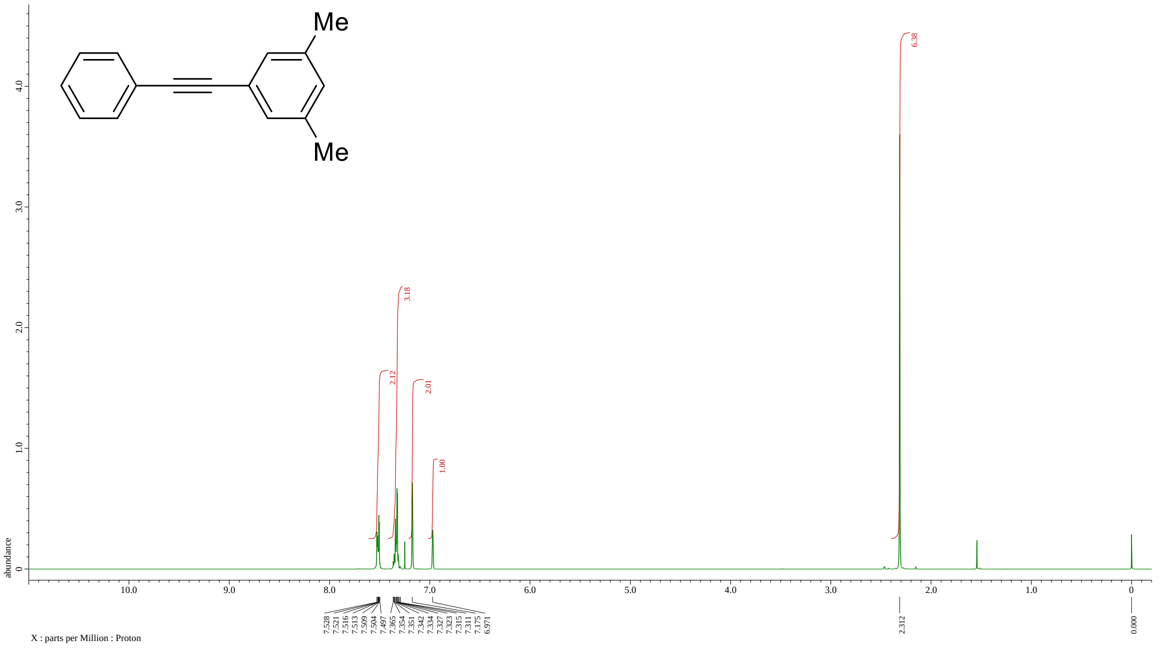

Supplementary Fig. 30 ^{1}H NMR (400 MHz, $CDCl_3$) of 1-(3,5-dimethylphenyl)-2-phenylacetylene (1l)

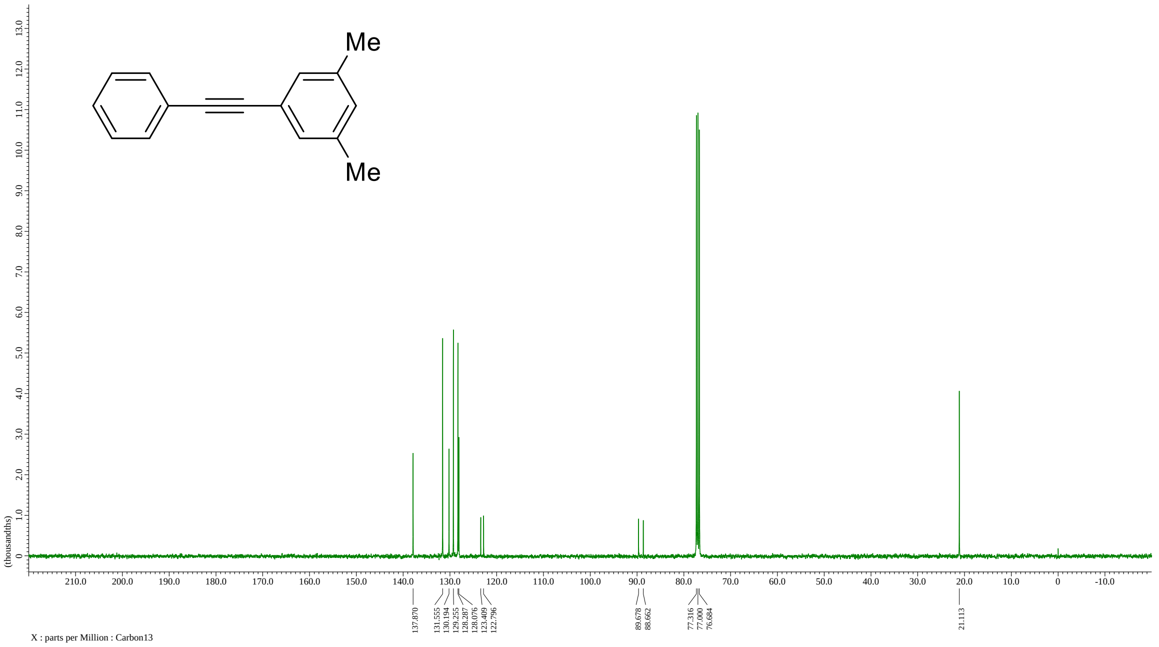

Supplementary Fig. 31 ^{13}C NMR (100 MHz, $CDCl_3$) of 1-(3,5-dimethylphenyl)-2-phenylacetylene (1l)

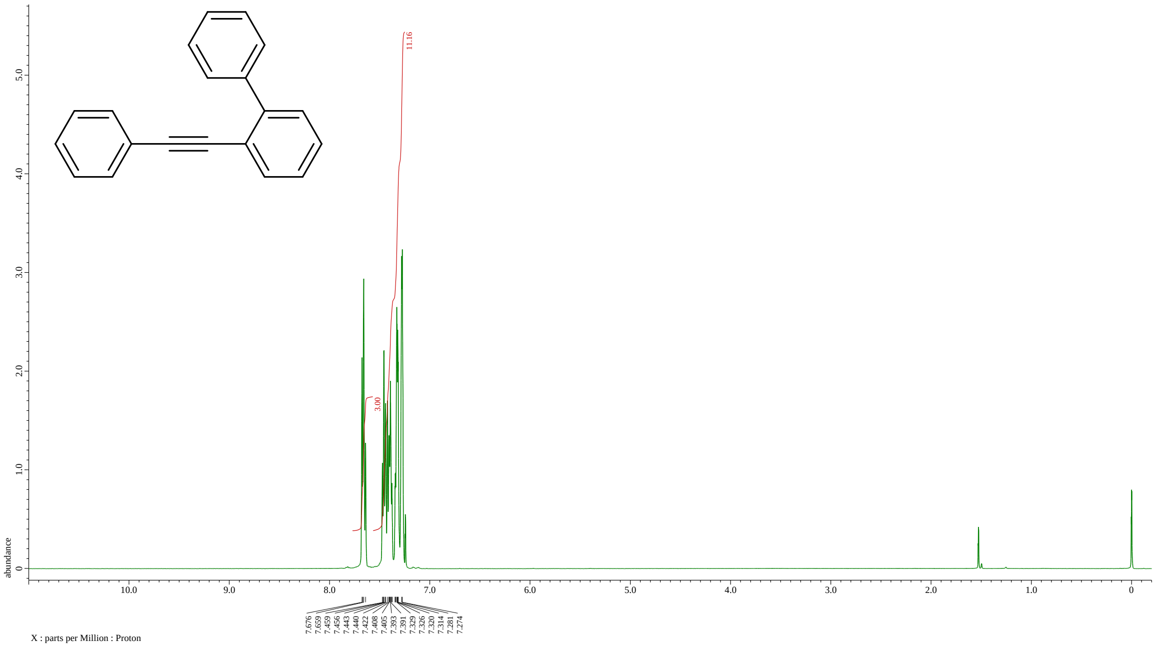

Supplementary Fig. 32 ^{1}H NMR (500 MHz, $CDCl_3$) of 1-(*o*-biphenylyl)-2-phenylacetylene (1m)

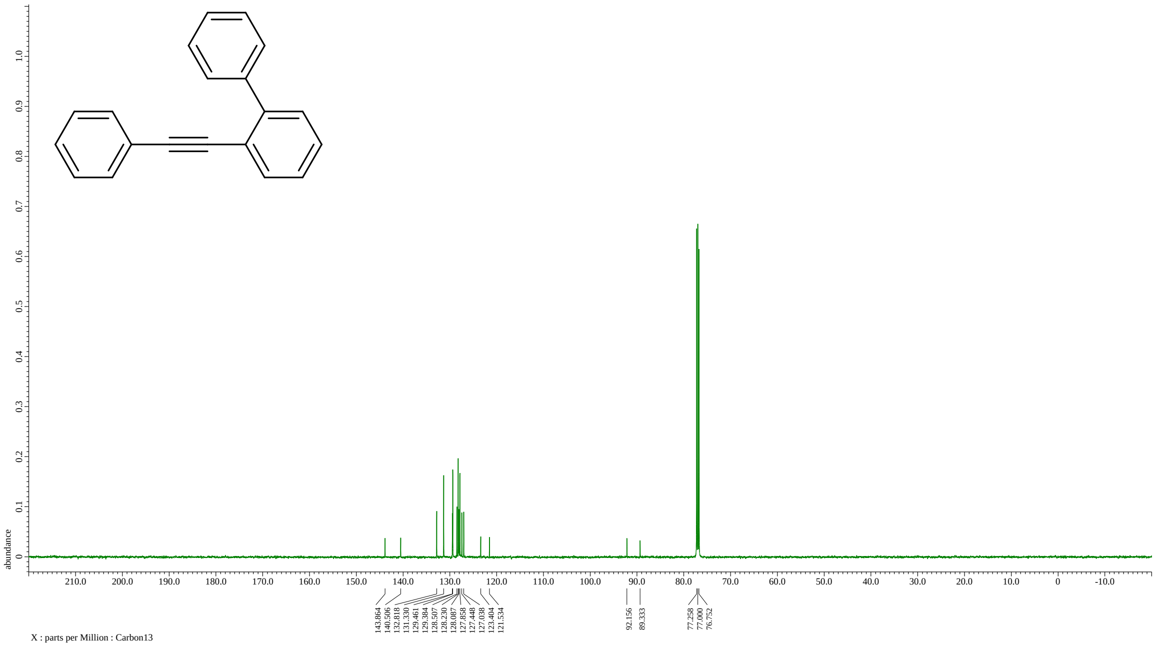

Supplementary Fig. 33 ^{13}C NMR (125 MHz, $CDCl_3$) of 1-(*o*-biphenylyl)-2-phenylacetylene (1m)

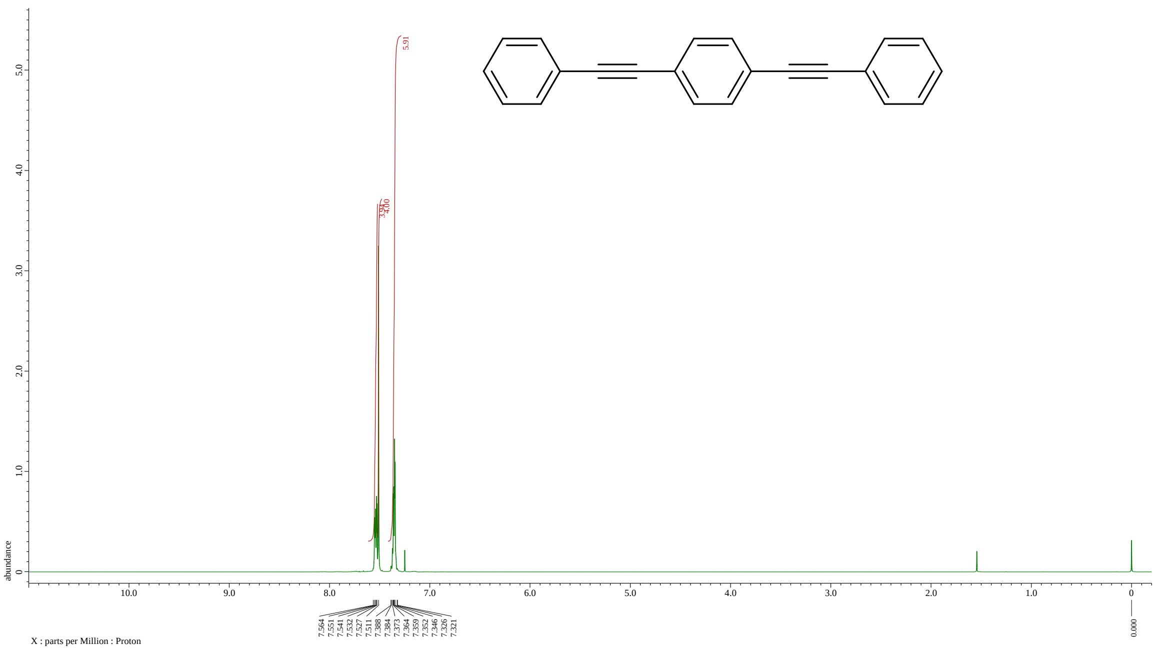

Supplementary Fig. 34 ^{1}H NMR (400 MHz, $CDCl_3$) of 1,4-bis(phenylethynyl)benzene (1k)

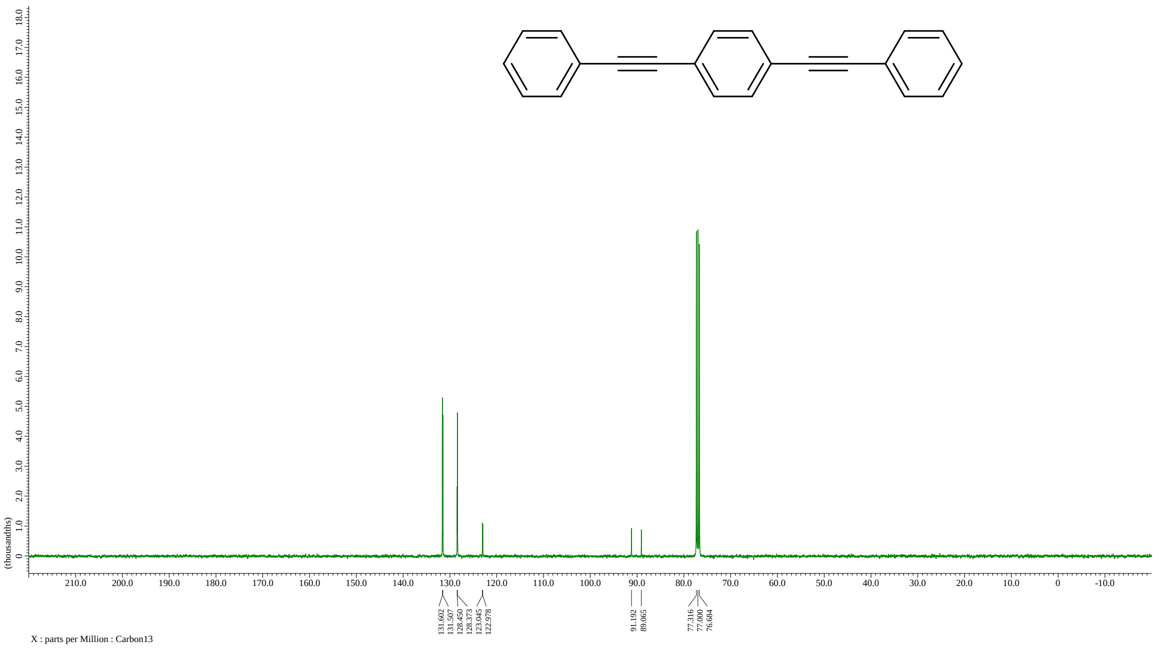

Supplementary Fig. 35 ^{13}C NMR (100 MHz, $CDCl_3$) of 1,4-bis(phenylethynyl)benzene (1k)

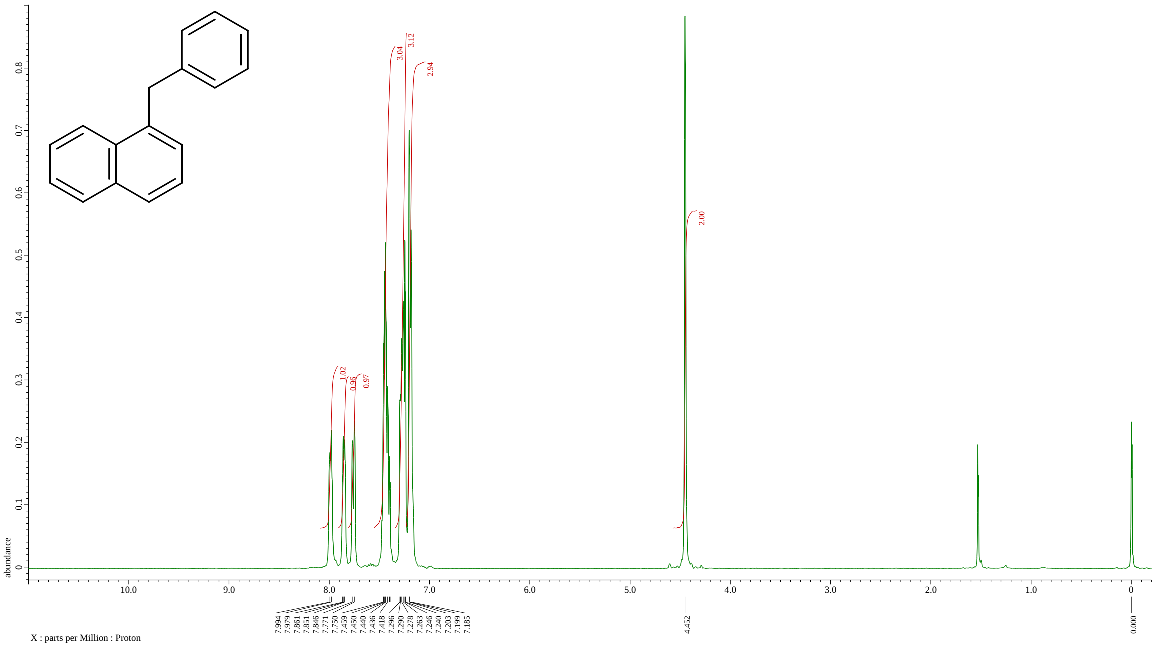

Supplementary Fig. 36 ^{1}H NMR (400 MHz, $CDCl_3$) of 1-benzylnaphthalene (8)

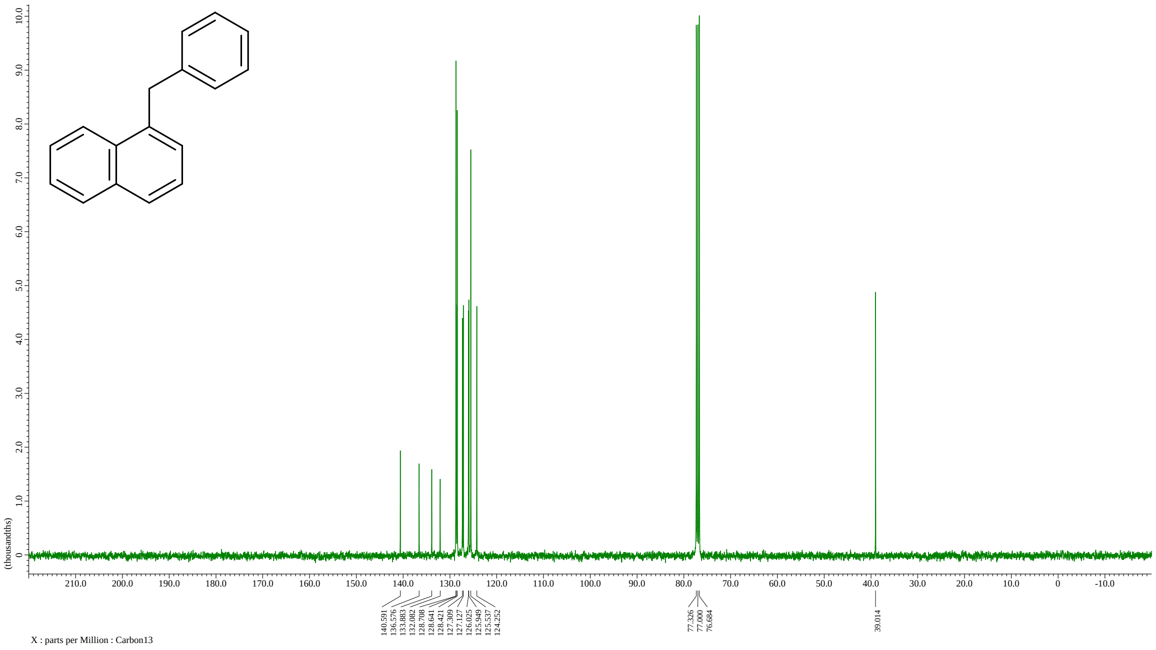

Supplementary Fig. 37 ^{13}C NMR (100 MHz, $CDCl_3$) of 1-benzylnaphthalene (8)

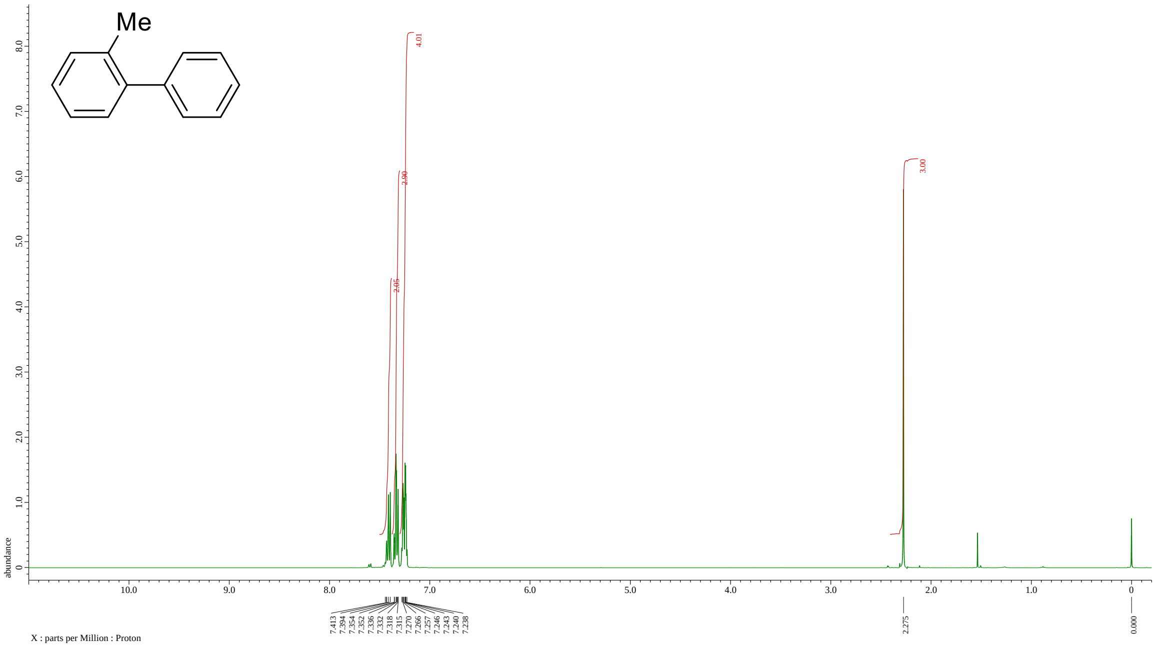

Supplementary Fig. 38 ^{1}H NMR (400 MHz, $CDCl_3$) of 2-methylbiphenyl (16)

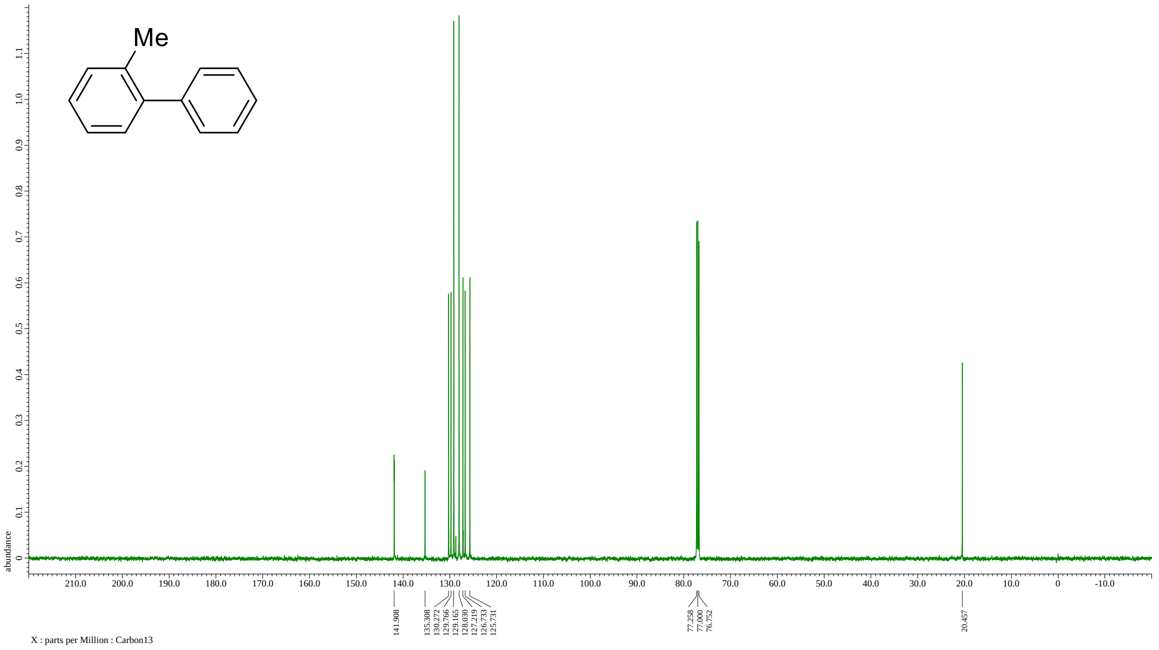

Supplementary Fig. 39 ^{13}C NMR (125 MHz, $CDCl_3$) of 2-methylbiphenyl (16)

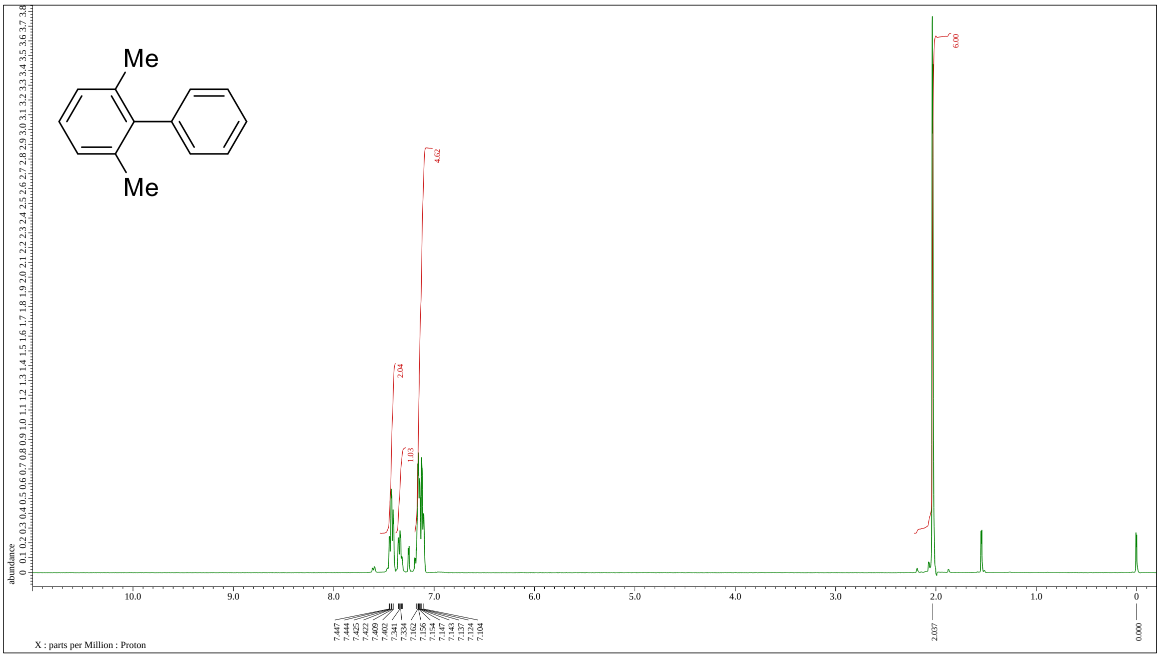

Supplementary Fig. 40 ^{1}H NMR (400 MHz, $CDCl_3$) of 2,6-dimethylbiphenyl (17)

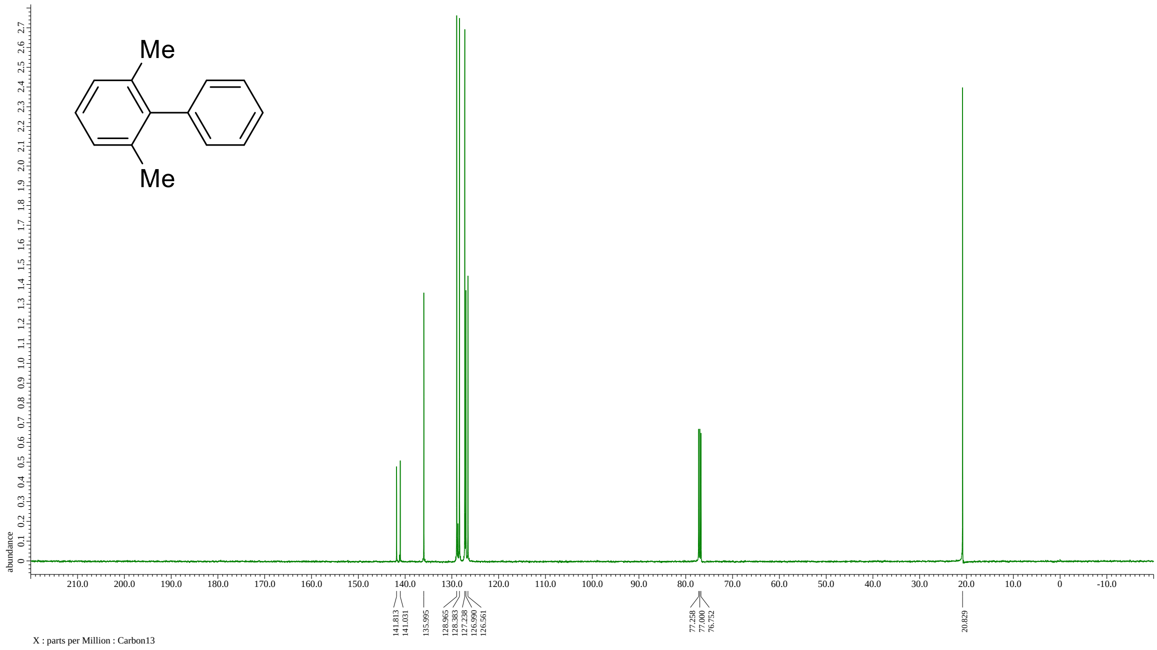

Supplementary Fig. 41 ^{13}C NMR (100 MHz, $CDCl_3$) of 2,6-dimethylbiphenyl (17)

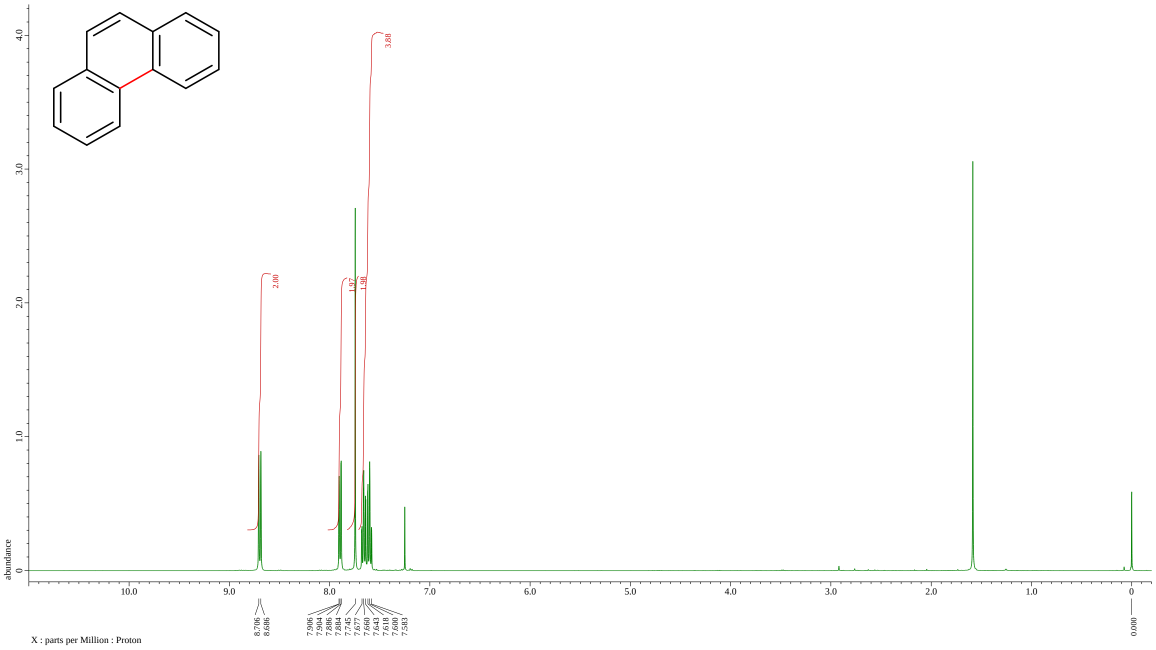

Supplementary Fig. 42 ^{1}H NMR (400 MHz, $CDCl_3$) of phenanthrene (2a)

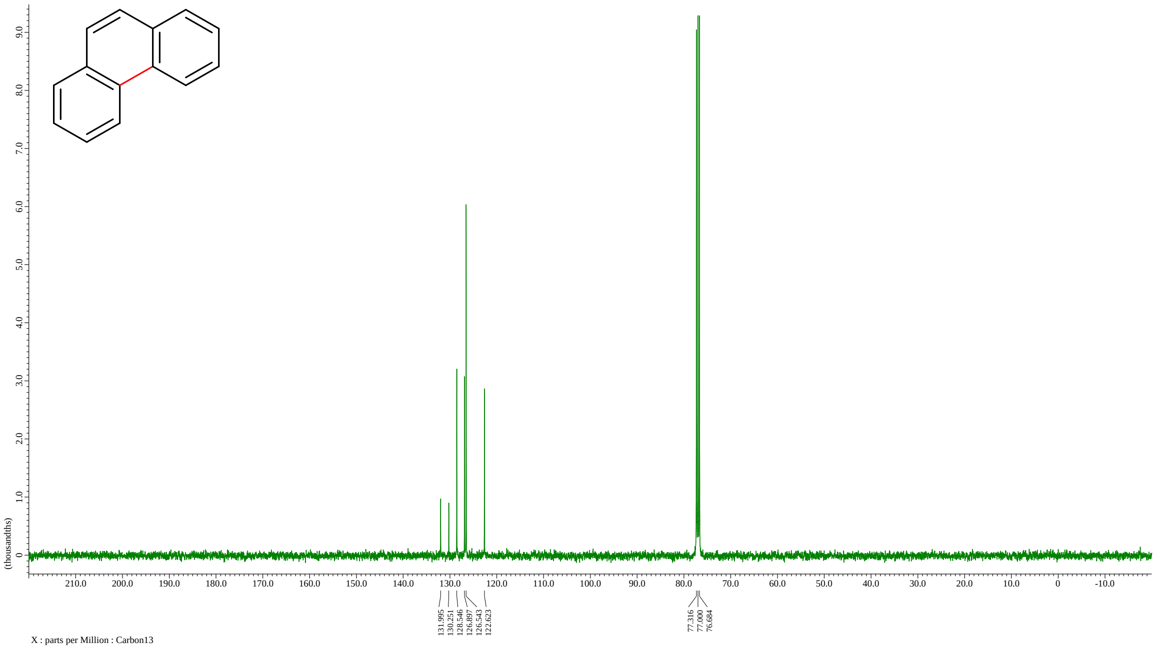

Supplementary Fig. 43 ^{13}C NMR (100 MHz, $CDCl_3$) of phenanthrene (2a)

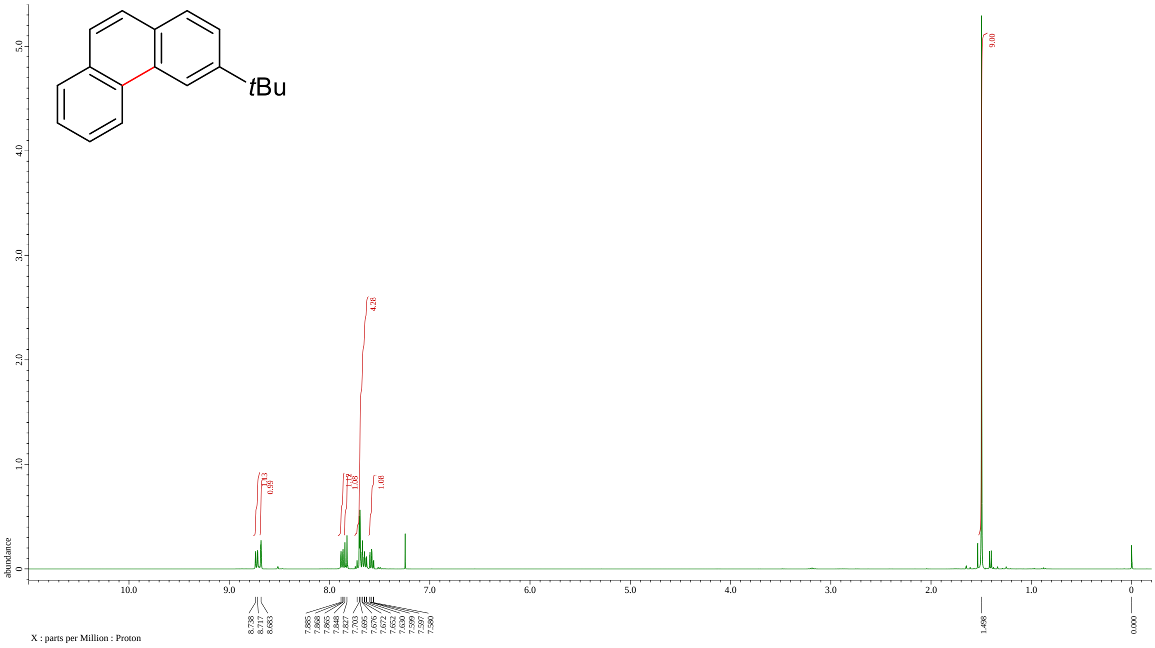

Supplementary Fig. 44 ^{1}H NMR (400 MHz, $CDCl_3$) of 3-*tert*-butylphenanthrene (2b)

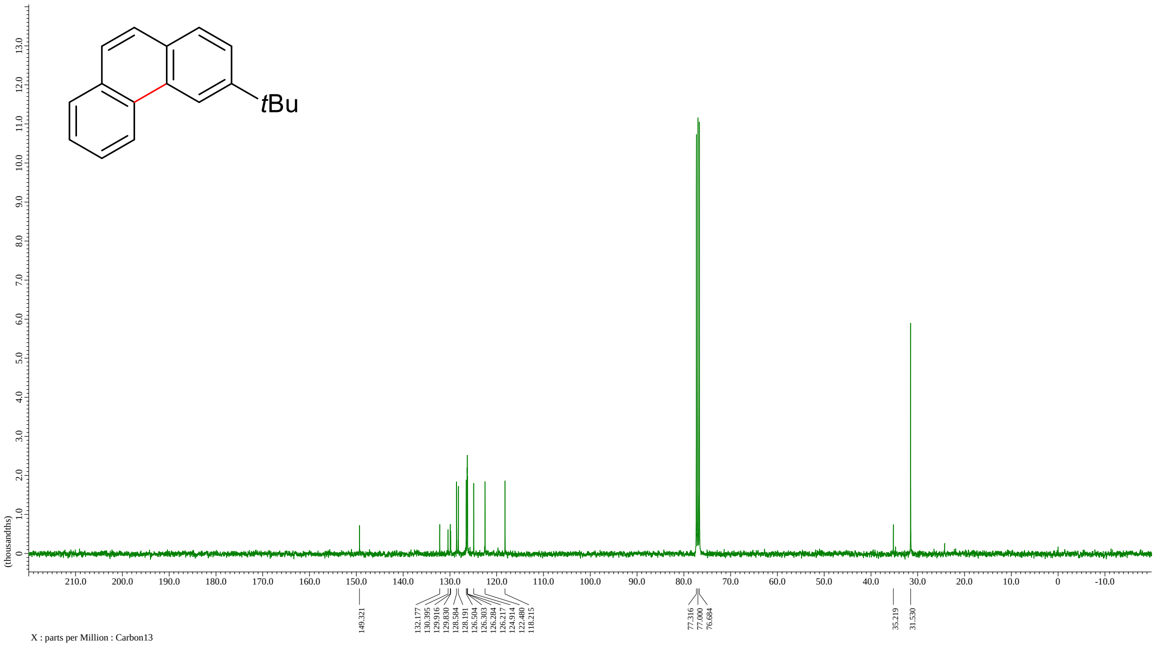

Supplementary Fig. 45 ^{13}C NMR (100 MHz, $CDCl_3$) of 3-*tert*-butylphenanthrene (2b)

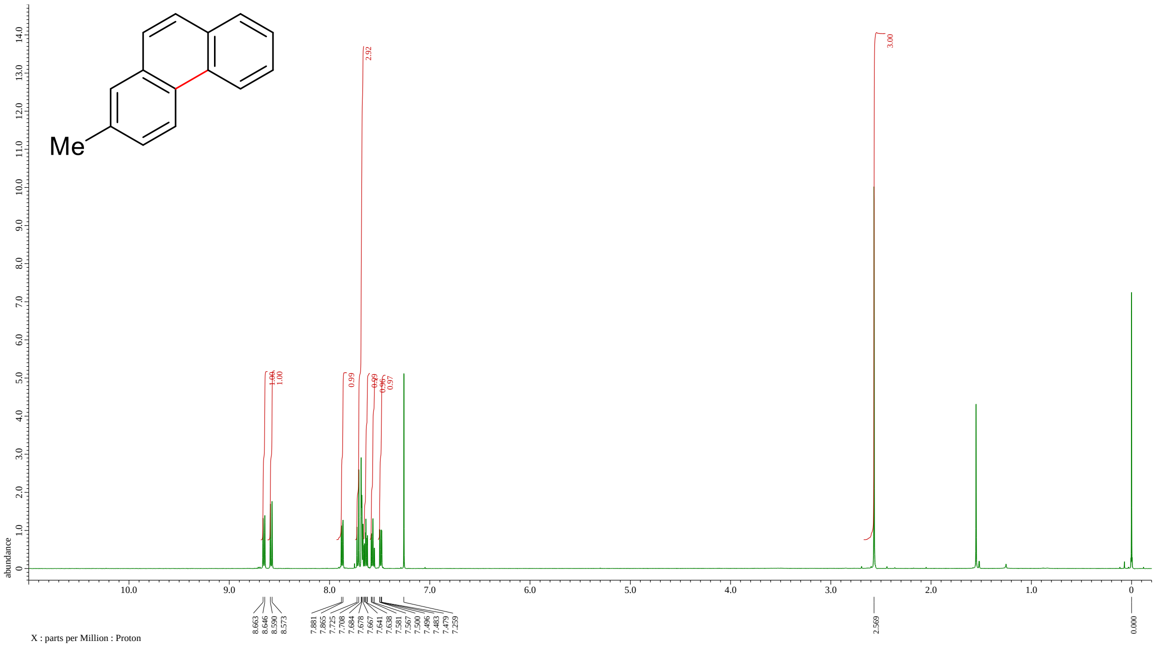

Supplementary Fig. 46 ^{1}H NMR (500 MHz, $CDCl_3$) of 2-methylphenanthrene (2c)

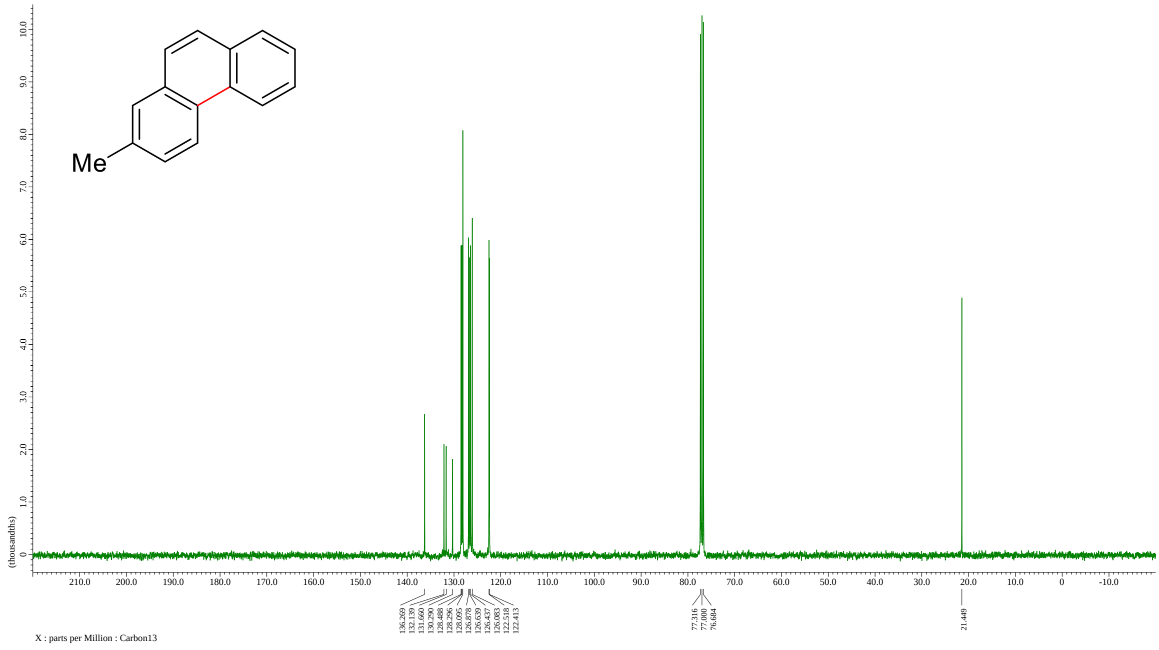

Supplementary Fig. 47 ^{13}C NMR (100 MHz, $CDCl_3$) of 2-methylphenanthrene (2c)

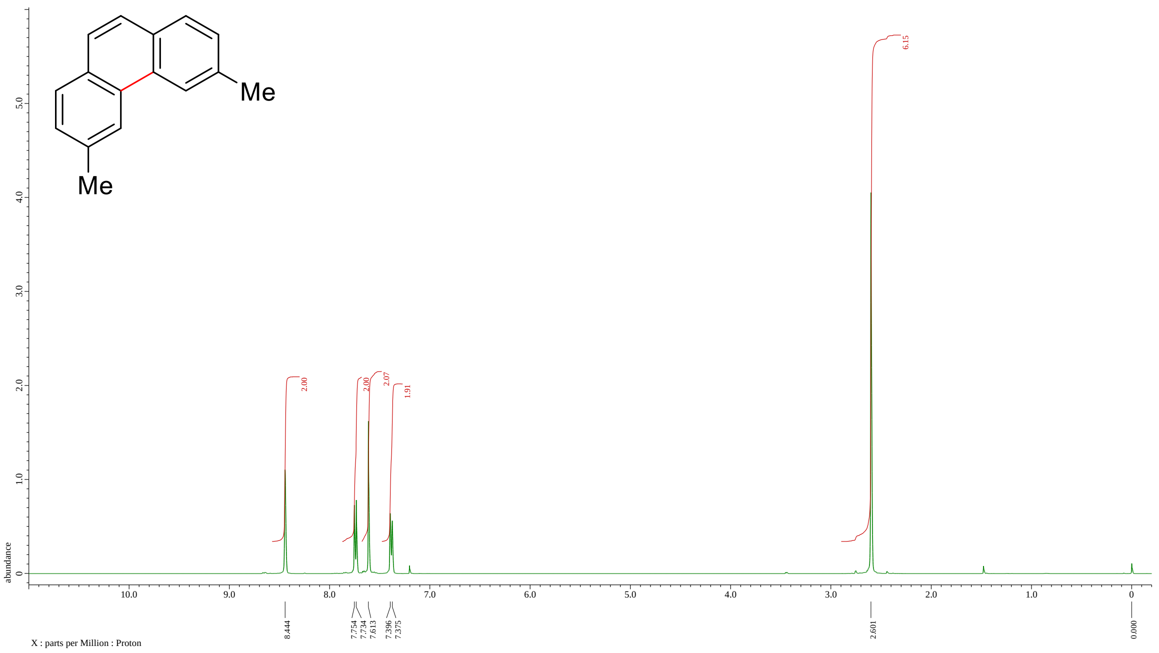

Supplementary Fig. 48 ^{1}H NMR (400 MHz, $CDCl_3$) of 3,6-dimethylphenanthrene (2d)

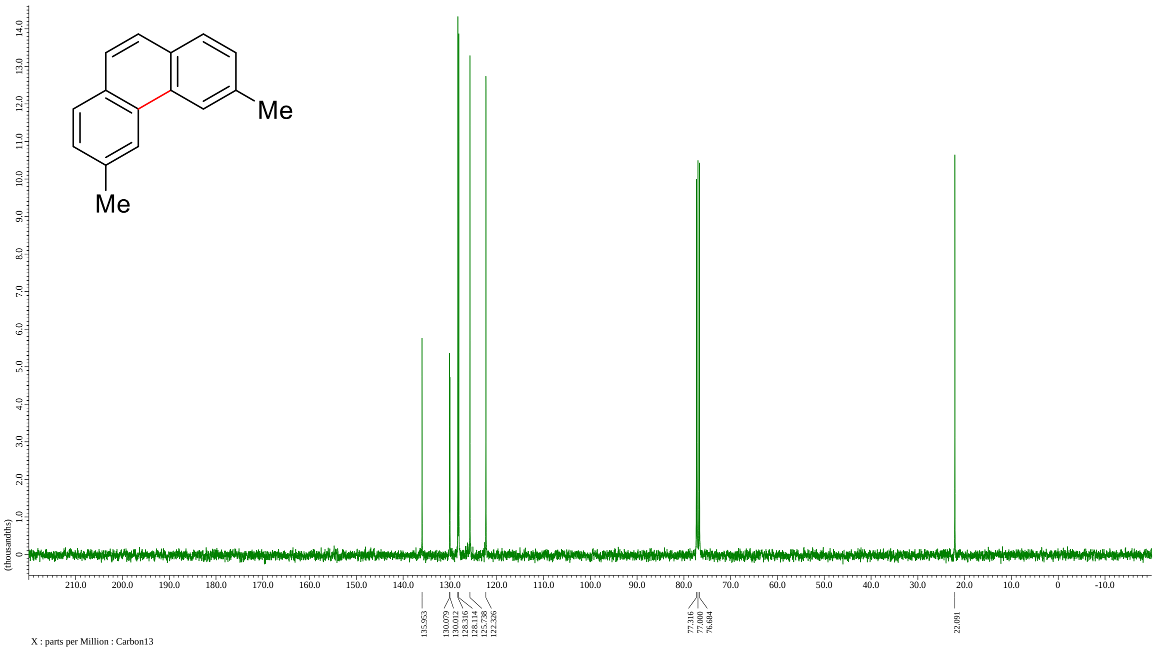

Supplementary Fig. 49 ^{13}C NMR (100 MHz, $CDCl_3$) of 3,6-dimethylphenanthrene (2d)

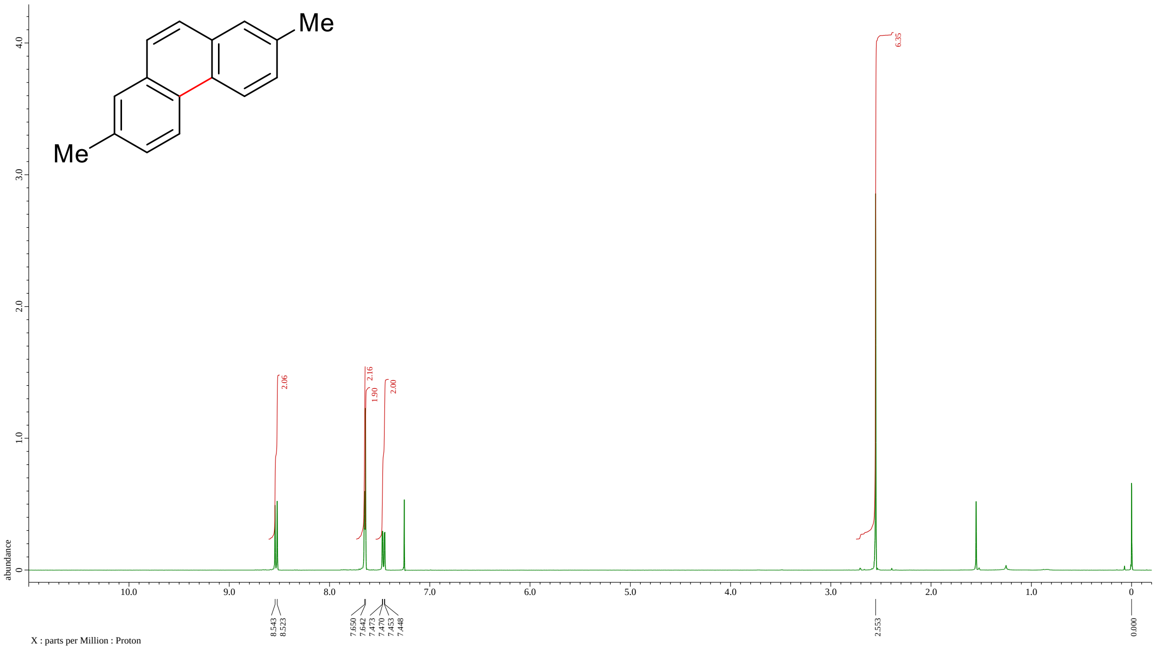

Supplementary Fig. 50 ^{1}H NMR (400 MHz, $CDCl_3$) of 2,7-dimethylphenanthrene (2e)

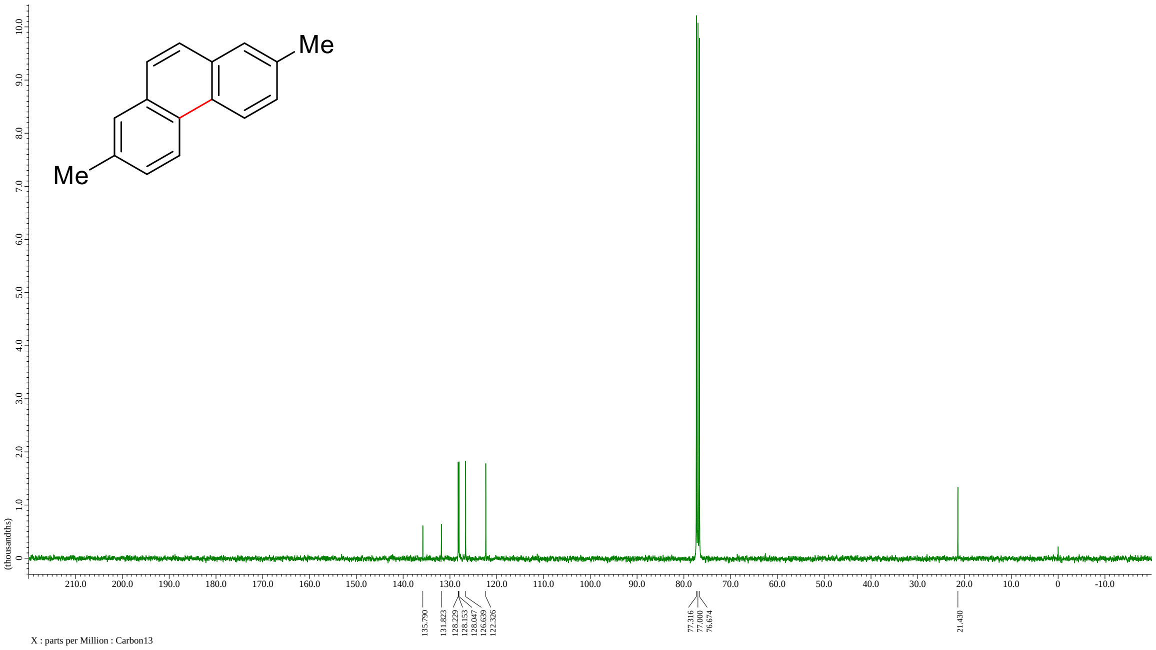

Supplementary Fig. 51 ^{13}C NMR (100 MHz, $CDCl_3$) of 2,7-dimethylphenanthrene (2e)

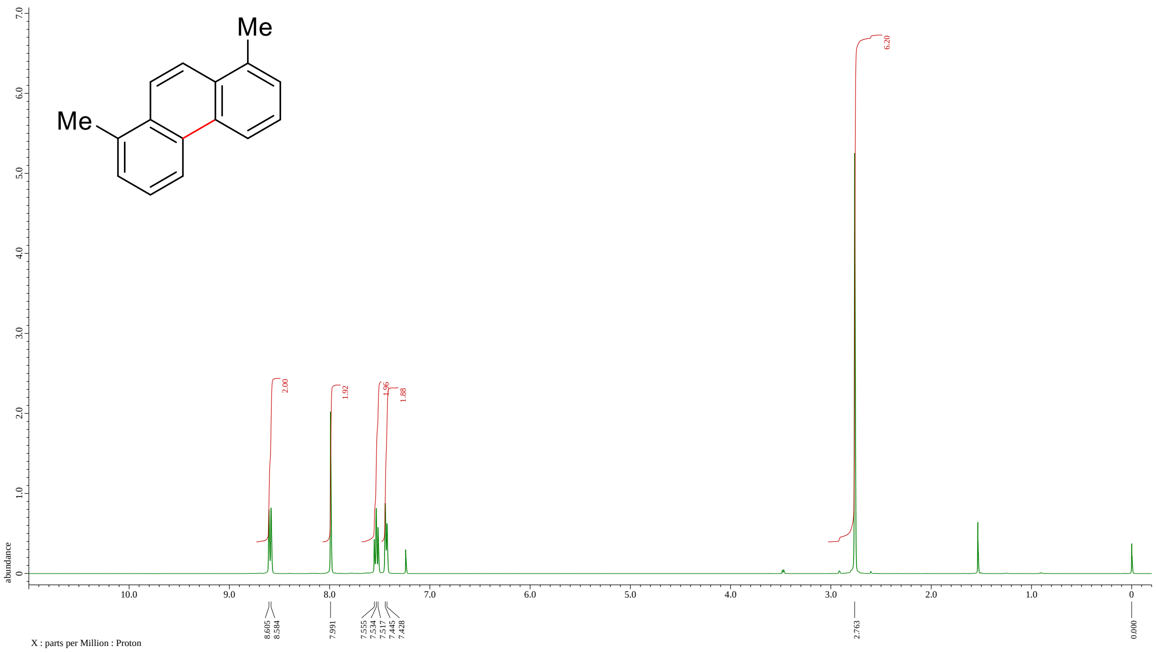

Supplementary Fig. 52 ^{1}H NMR (400 MHz, $CDCl_3$) of 1,8-dimethylphenanthrene (2f)

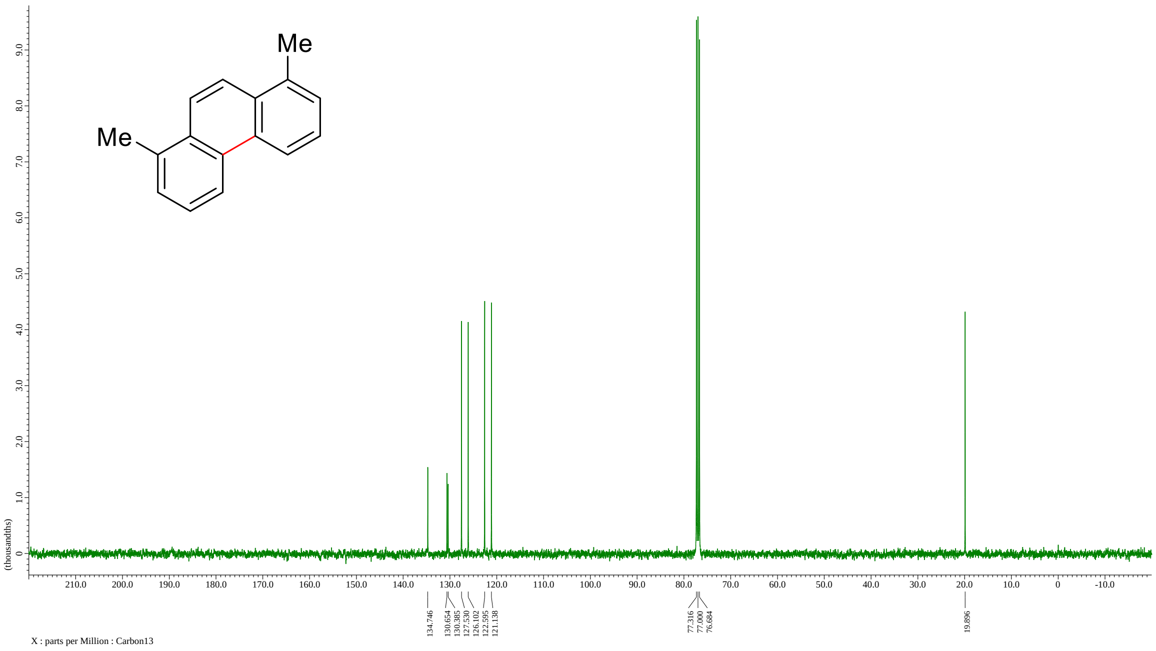

Supplementary Fig. 53 ^{13}C NMR (100 MHz, $CDCl_3$) of 1,8-dimethylphenanthrene (2f)

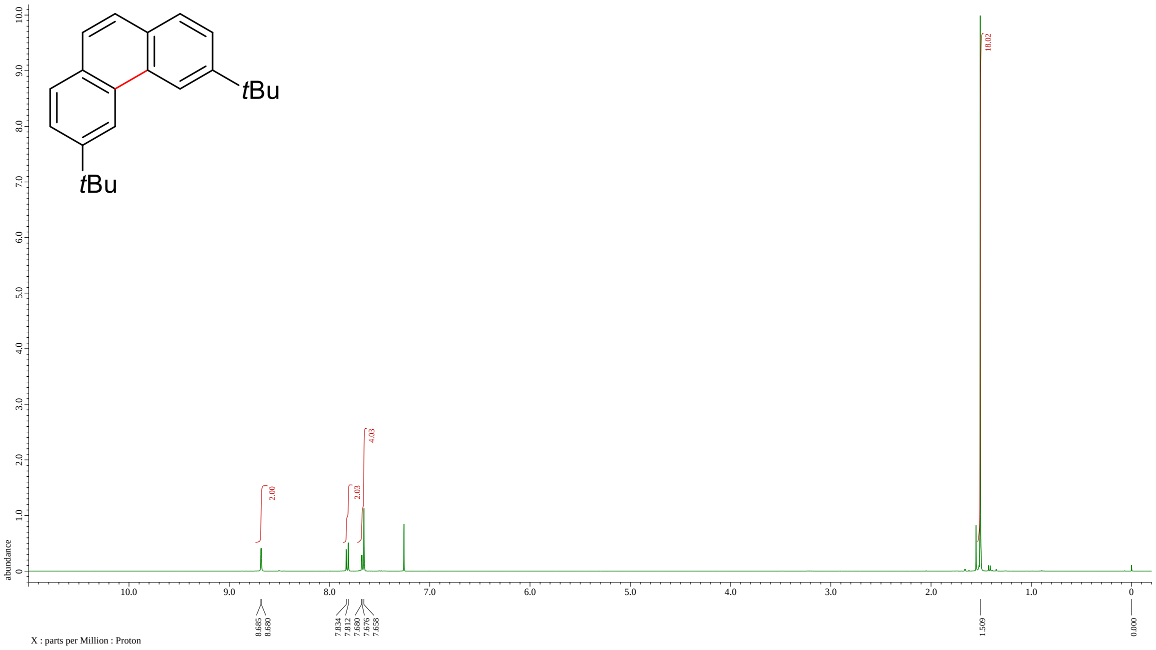

Supplementary Fig. 54 ^{1}H NMR (400 MHz, $CDCl_3$) of 3,6-di-*tert*-butylphenanthrene (2g)

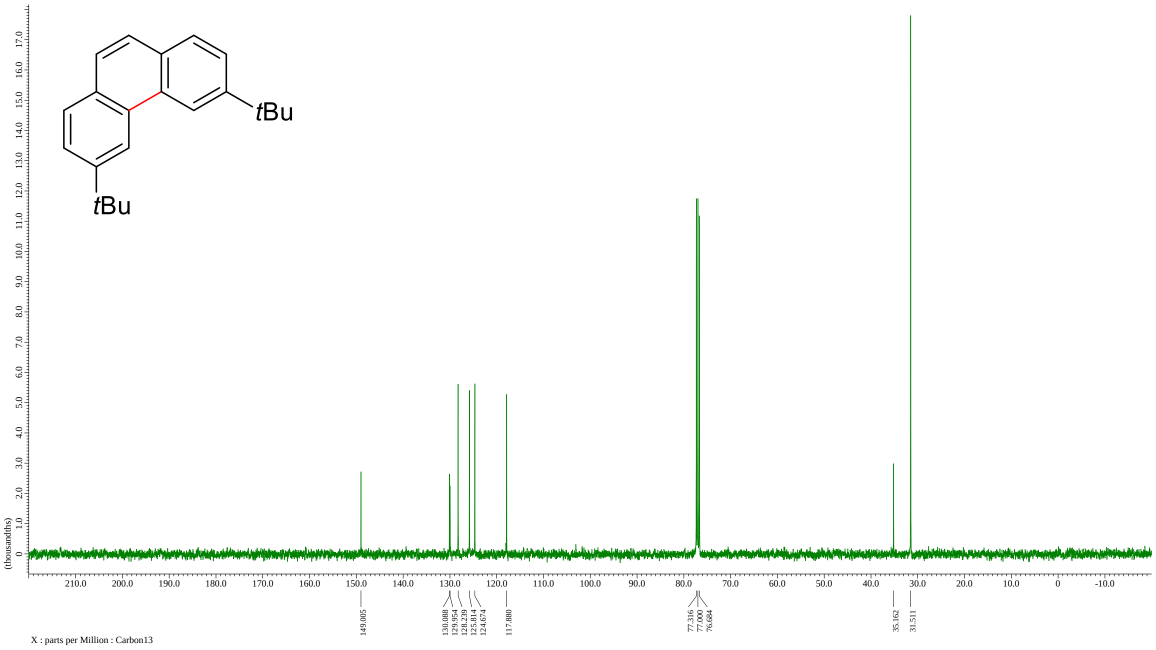

Supplementary Fig. 55 ^{13}C NMR (100 MHz, $CDCl_3$) of 3,6-di-*tert*-butylphenanthrene (2g)

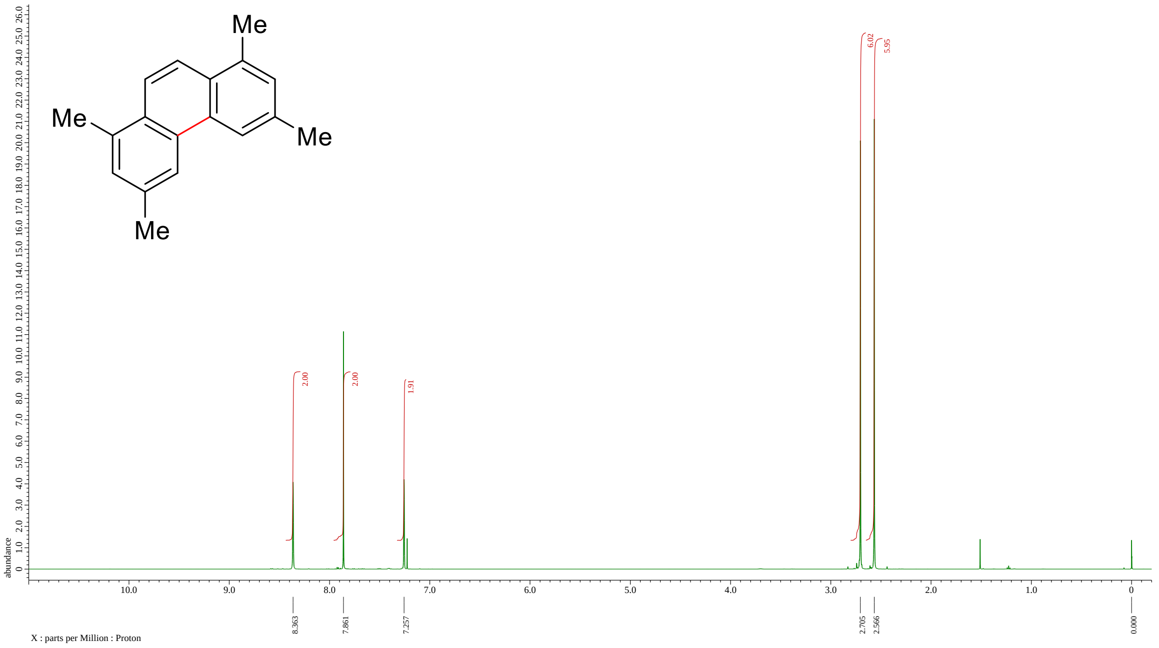

Supplementary Fig. 56 ^{1}H NMR (400 MHz, $CDCl_3$) of 1,3,6,8-tetramethylphenanthrene (2h)

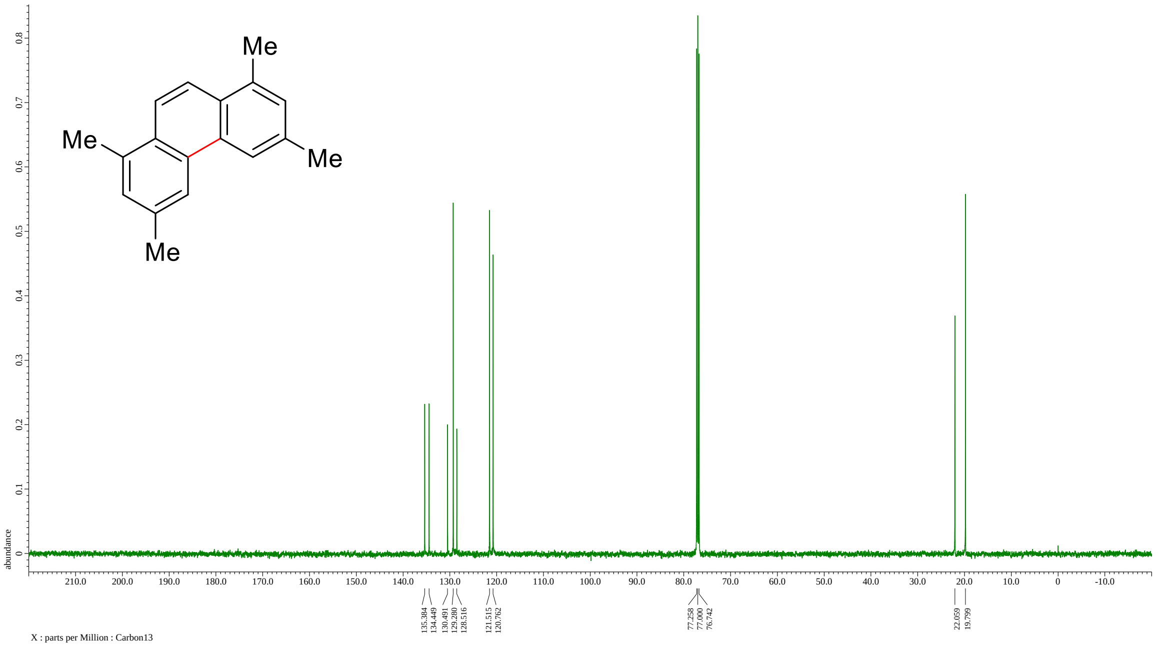

Supplementary Fig. 57 ^{13}C NMR (100 MHz, $CDCl_3$) of 1,3,6,8-tetramethylphenanthrene (2h)

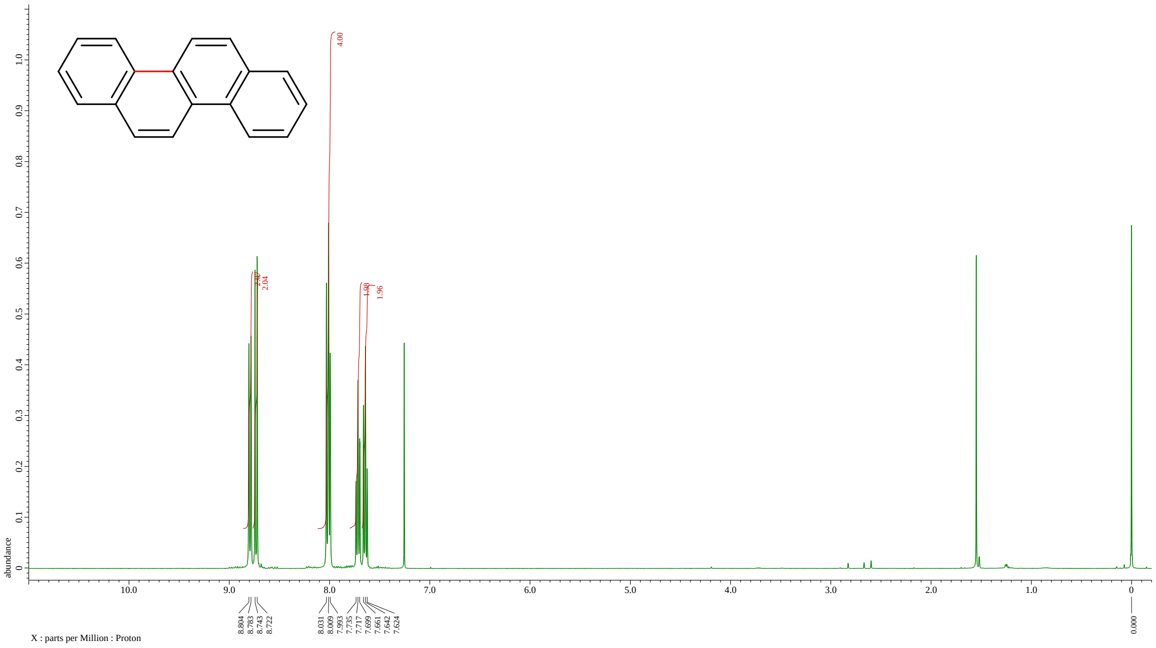

Supplementary Fig. 58 ^{1}H NMR (400 MHz, $CDCl_3$) of chrysene (2i)

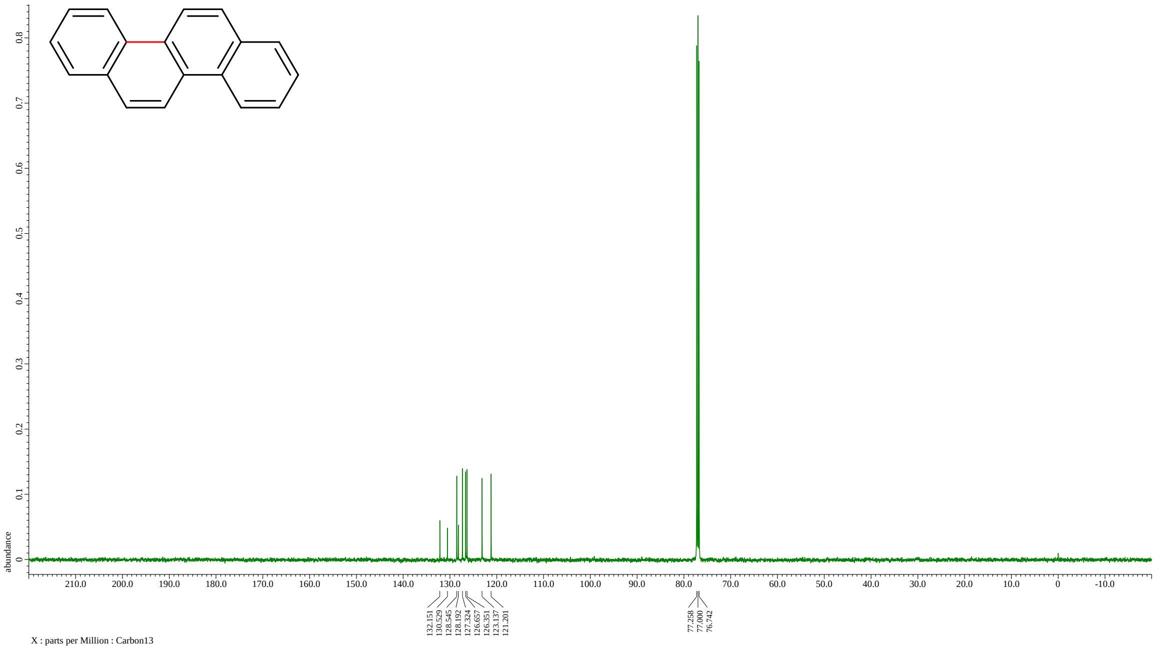

Supplementary Fig. 59 ^{13}C NMR (125 MHz, $CDCl_3$) of chrysene (2i)

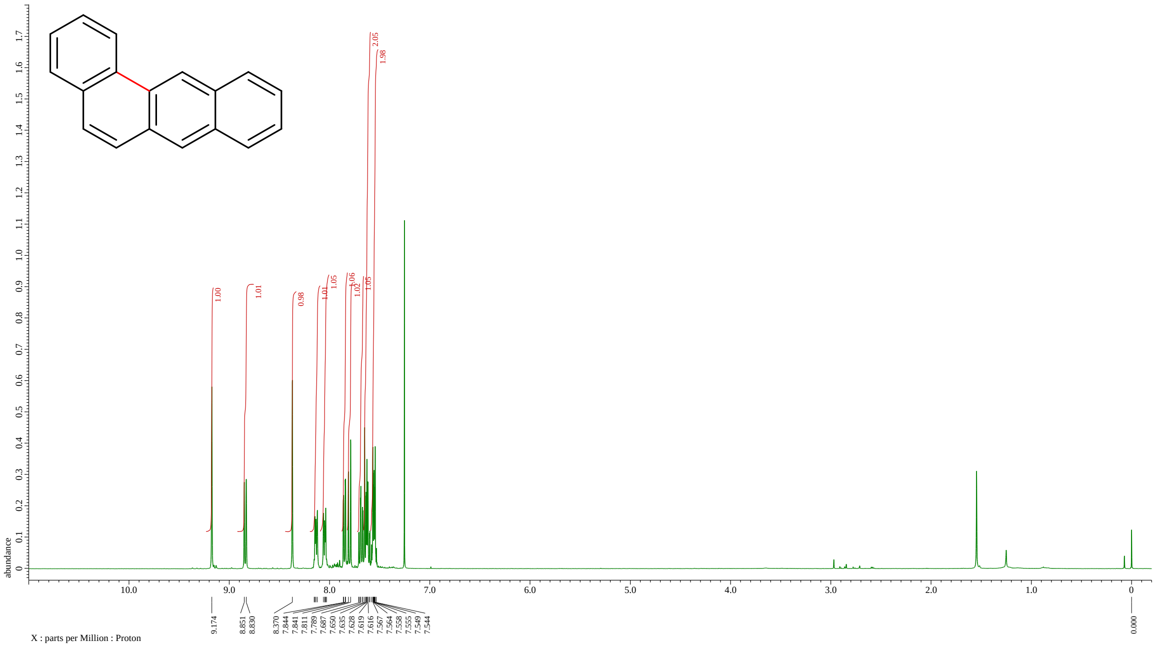

Supplementary Fig. 60 ^{1}H NMR (400 MHz, $CDCl_3$) of benzo[*a*]anthracene (2j)

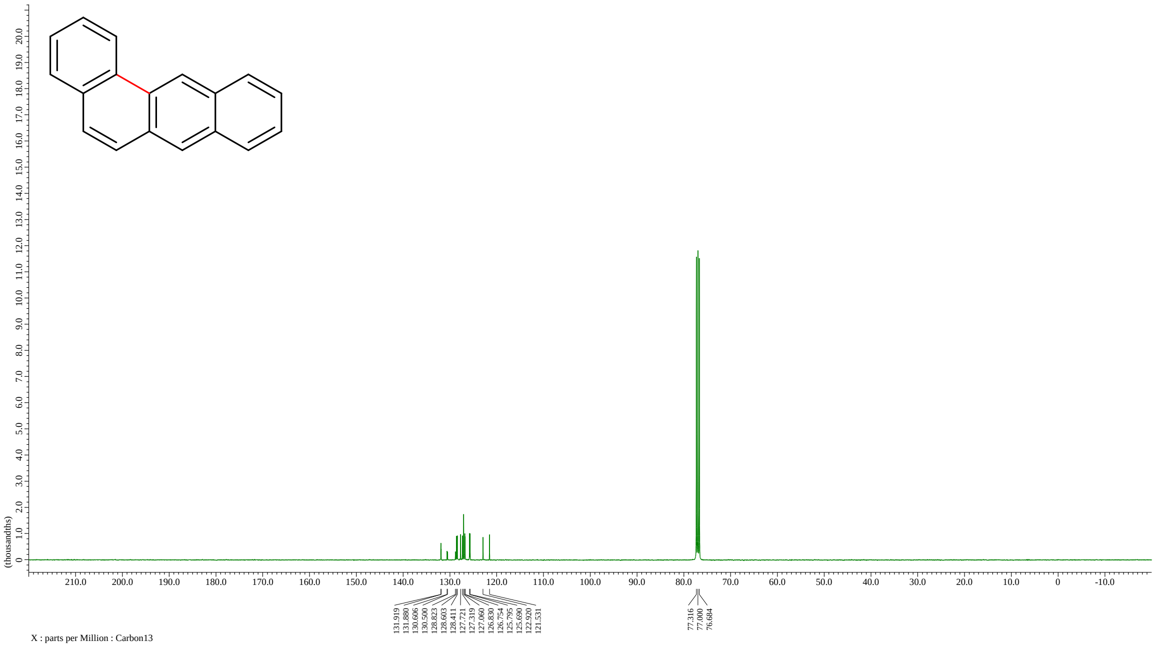

Supplementary Fig. 61 ^{13}C NMR (100 MHz, $CDCl_3$) of benzo[*a*]anthracene (2j)

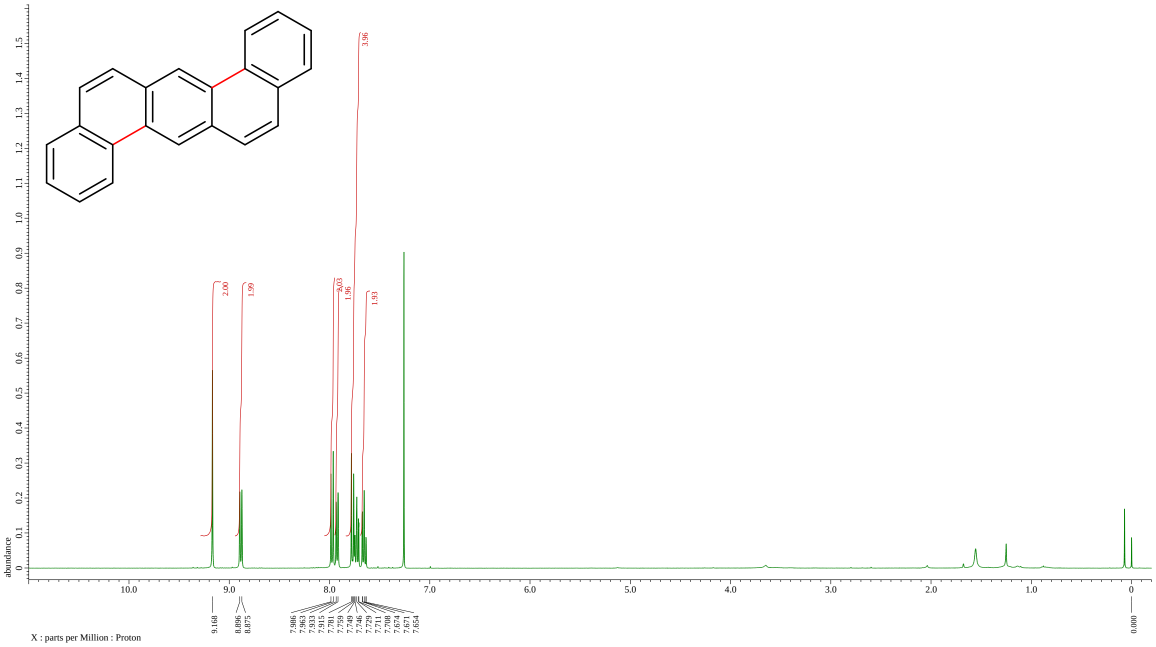

Supplementary Fig. 62 ^{1}H NMR (400 MHz, $CDCl_3$) of dibenzo[*a*,*h*]anthracene (2k)

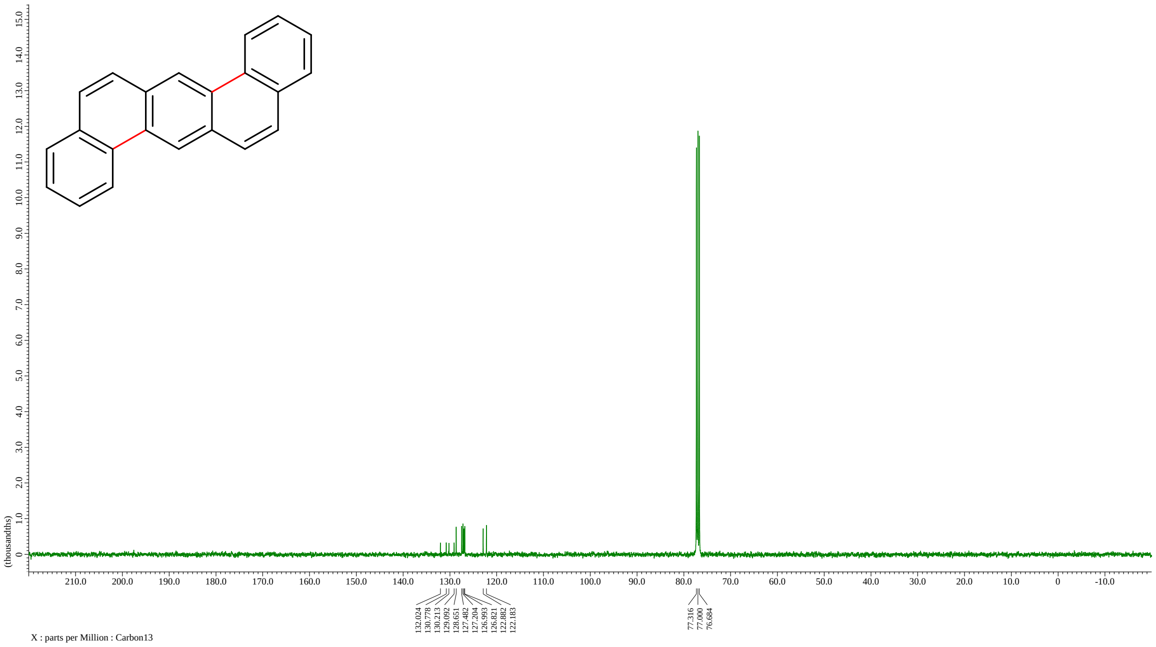

Supplementary Fig. 63 ^{13}C NMR (100 MHz, $CDCl_3$) of dibenzo[*a*,*h*]anthracene (2k)

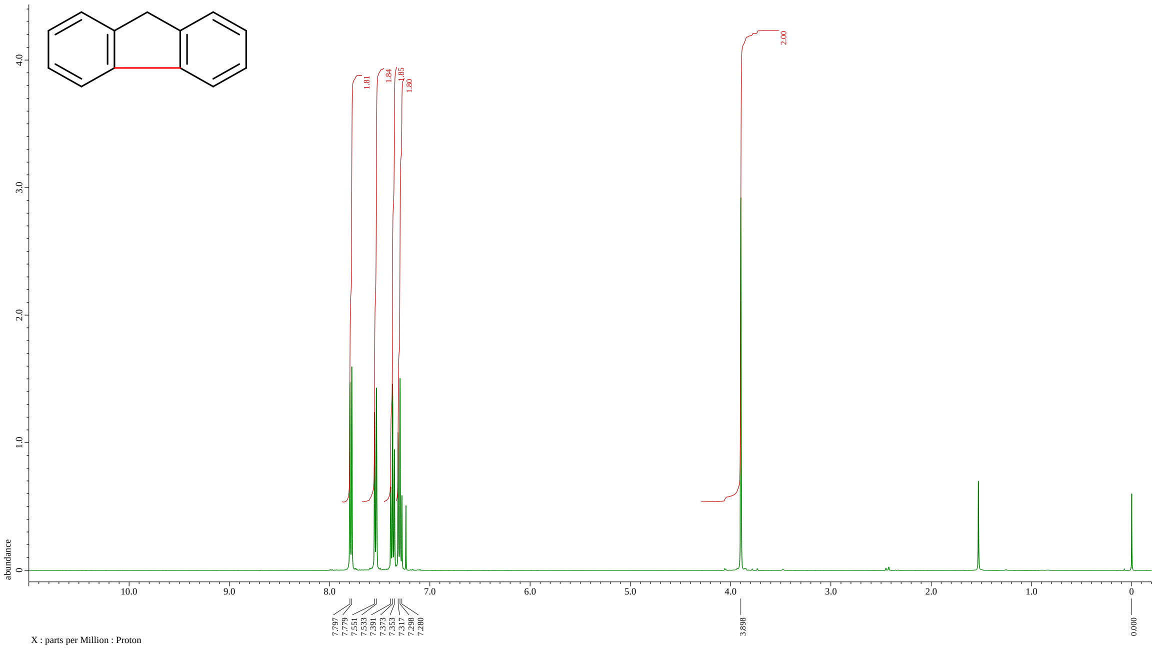

Supplementary Fig. 64 ^{1}H NMR (400 MHz, $CDCl_3$) of 9*H*-fluorene (7)

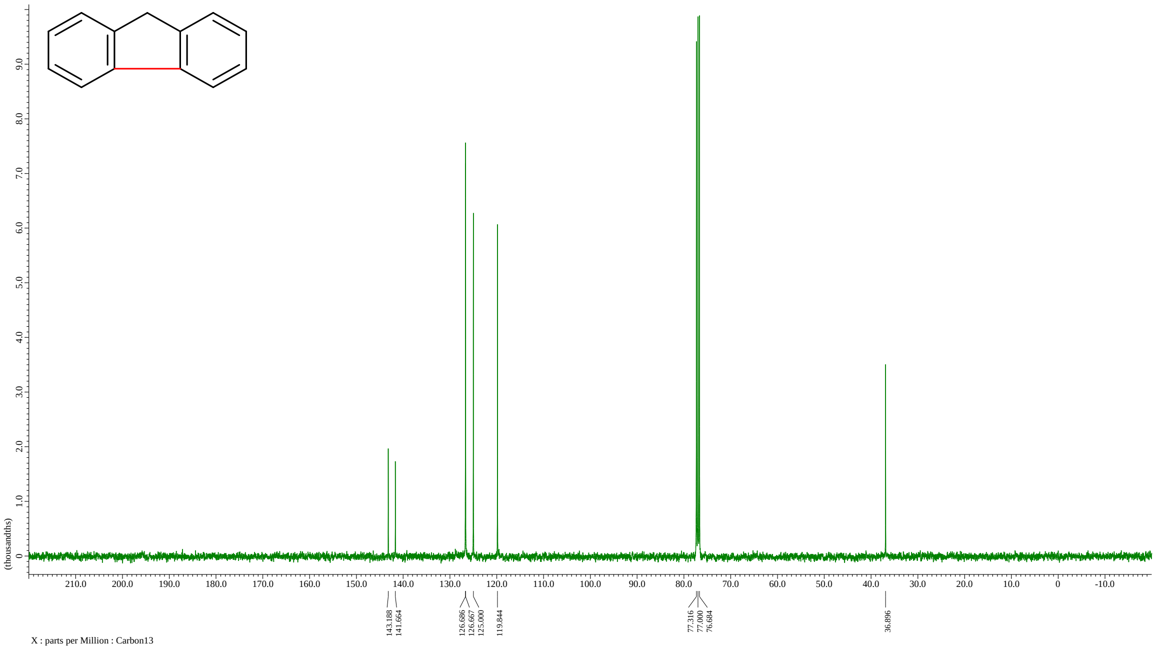

Supplementary Fig. 65 ^{13}C NMR (100 MHz, $CDCl_3$) of 9*H*-fluorene (7)

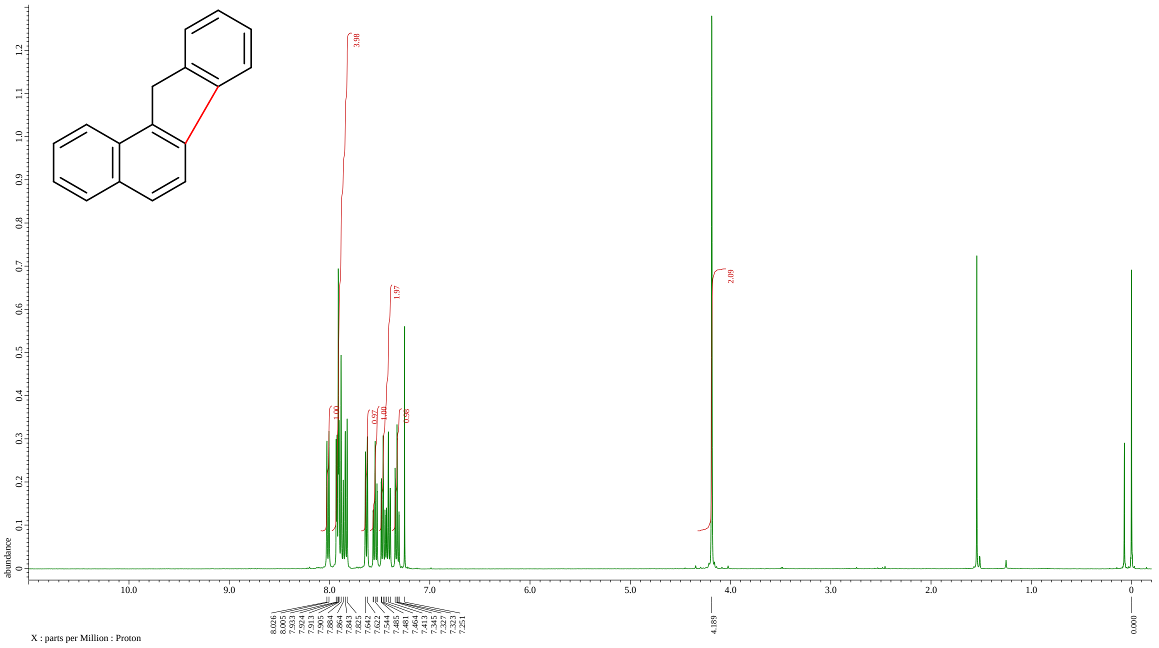

Supplementary Fig. 66 ^{1}H NMR (400 MHz, $CDCl_3$) of benzo[*a*]fluorene (9)

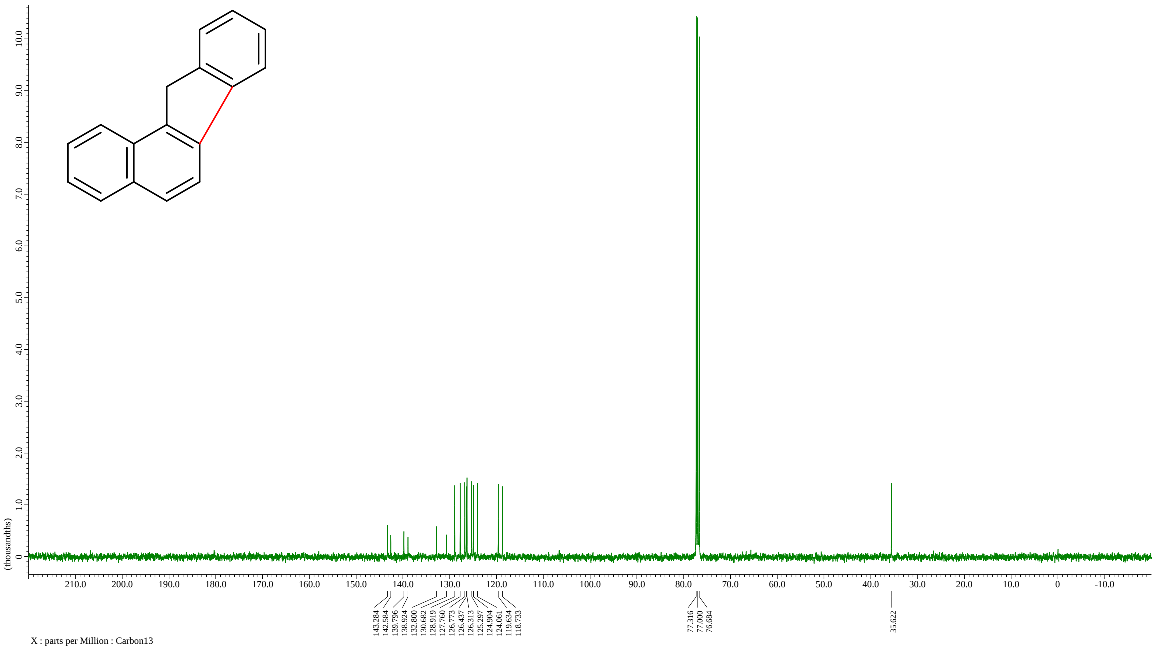

Supplementary Fig. 67 ^{13}C NMR (100 MHz, $CDCl_3$) of benzo[*a*]fluorene (9)

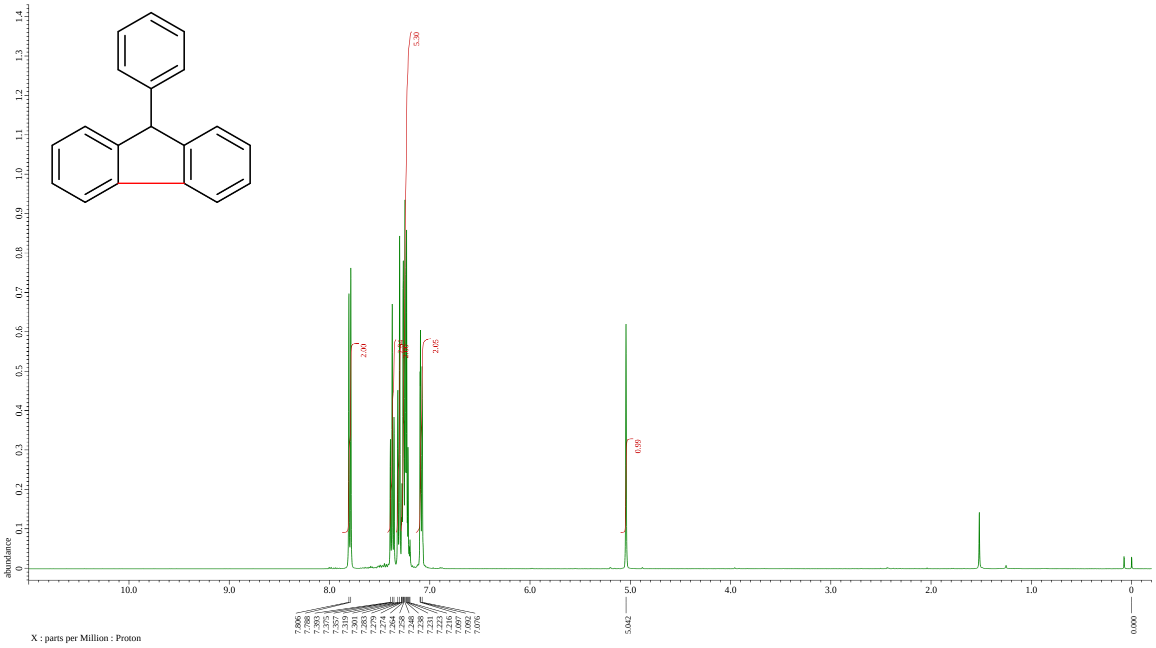

Supplementary Fig. 68 ^{1}H NMR (400 MHz, $CDCl_3$) of 9-phenylfluorene (11)

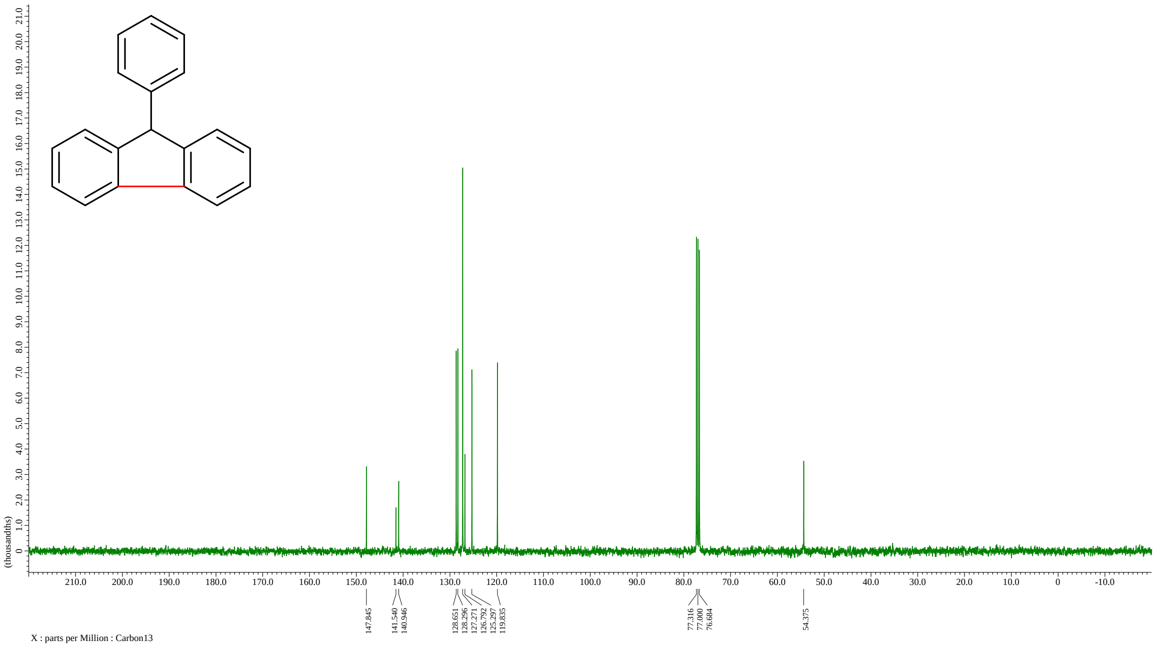

Supplementary Fig. 69 ^{13}C NMR (100 MHz, $CDCl_3$) of 9-phenylfluorene (11)

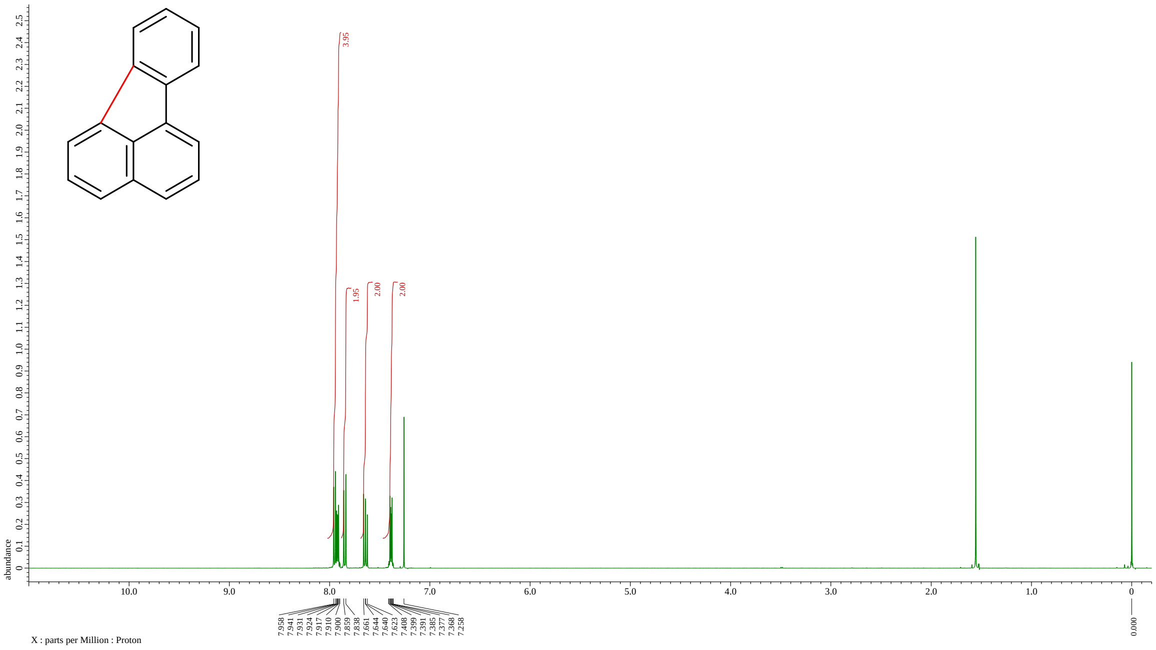

Supplementary Fig. 70 ^{1}H NMR (400 MHz, $CDCl_3$) of fluoranthene (13)

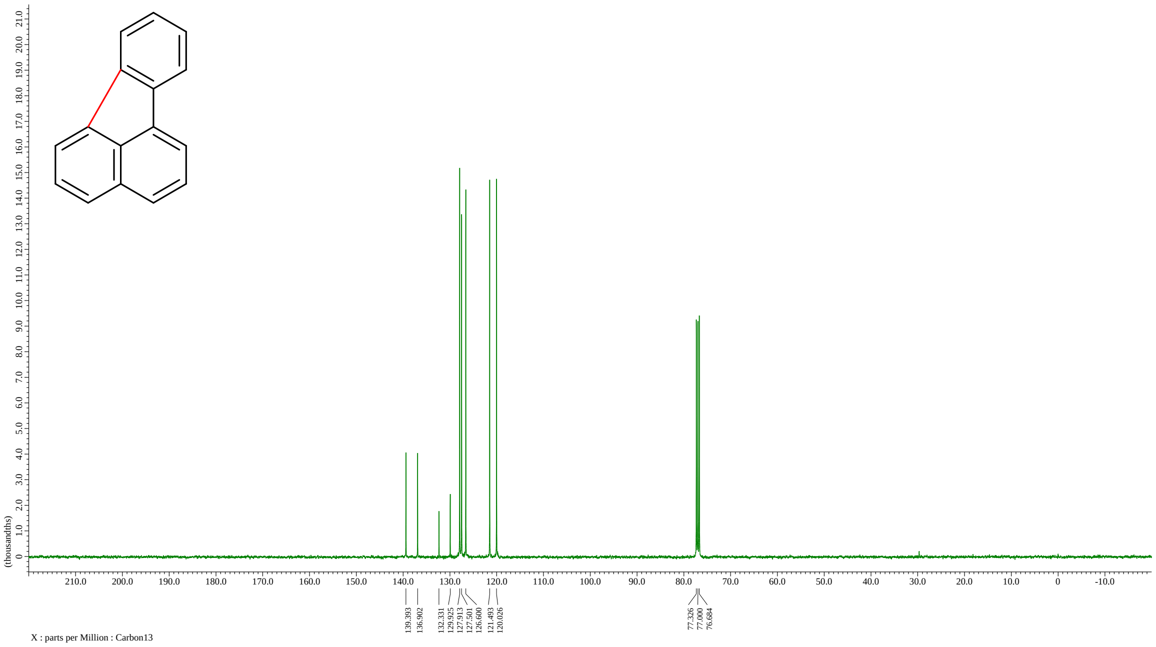

Supplementary Fig. 71 ^{13}C NMR (100 MHz, $CDCl_3$) of fluoranthene (13)

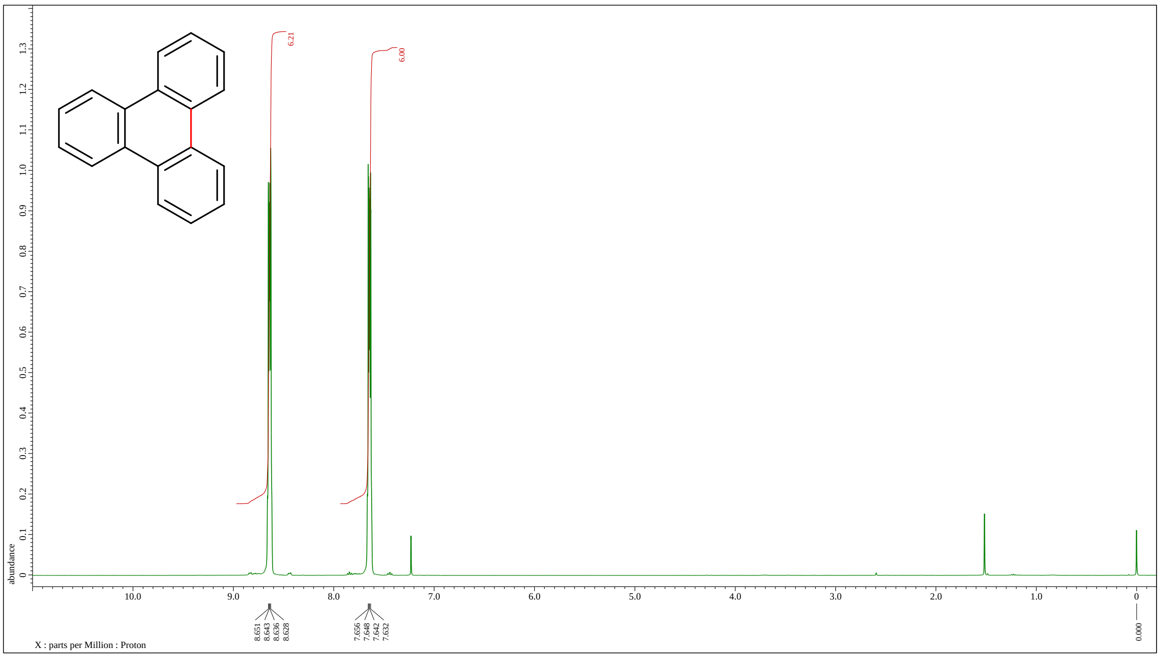

Supplementary Fig. 72 ^{1}H NMR (400 MHz, $CDCl_3$) of triphenylene (15)

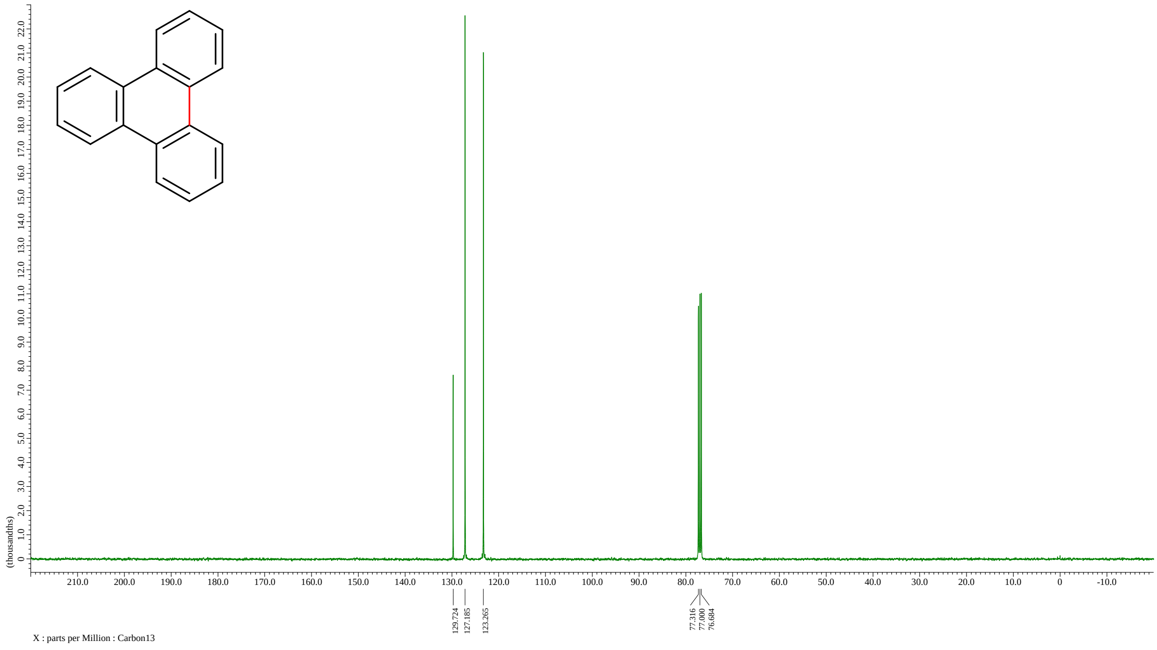

Supplementary Fig. 73 ^{13}C NMR (100 MHz, $CDCl_3$) of triphenylene (15)

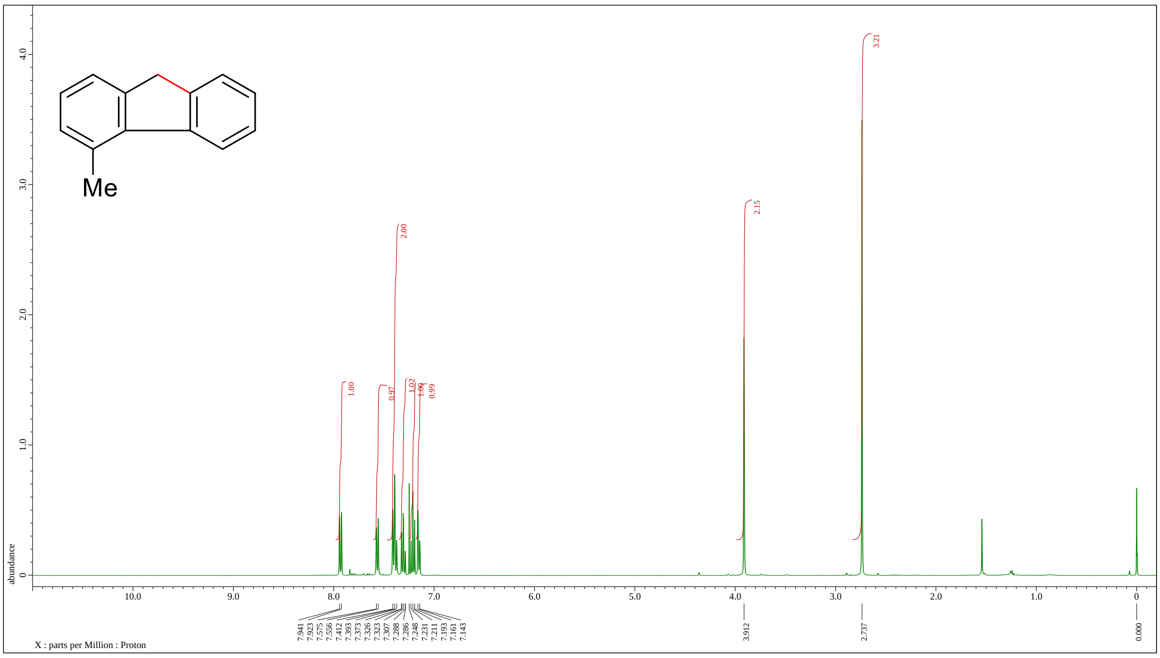

Supplementary Fig. 74 ^{1}H NMR (400 MHz, $CDCl_3$) of 4-methylfluorene (18)

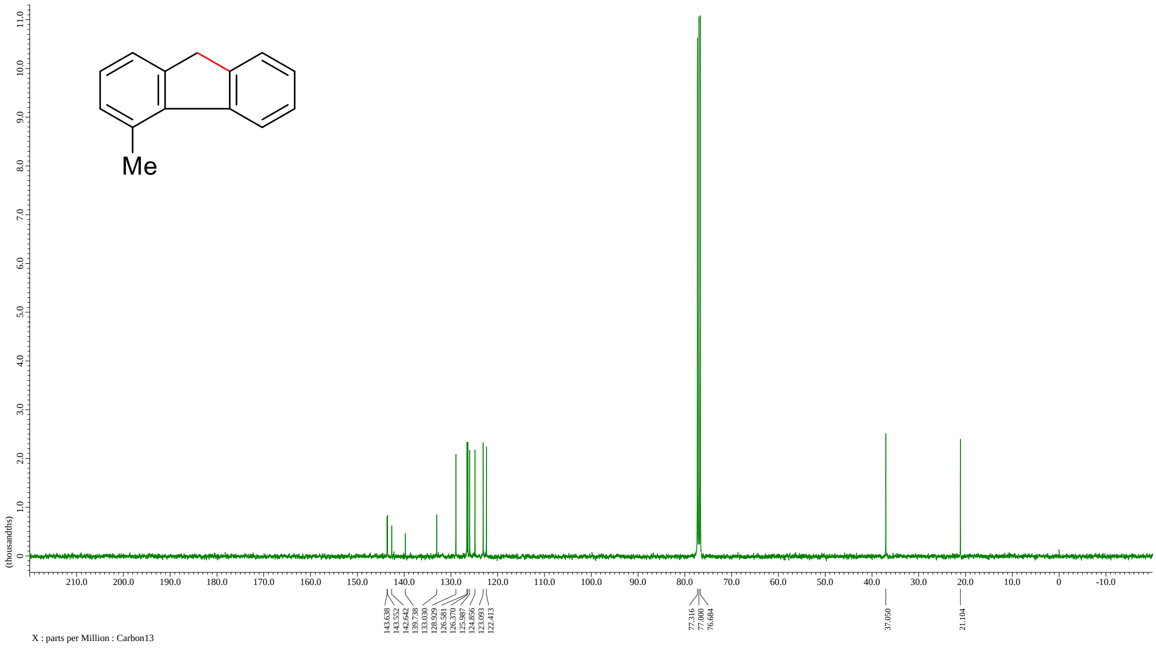

Supplementary Fig. 75 ^{13}C NMR (100 MHz, $CDCl_3$) of 4-methylfluorene (18)

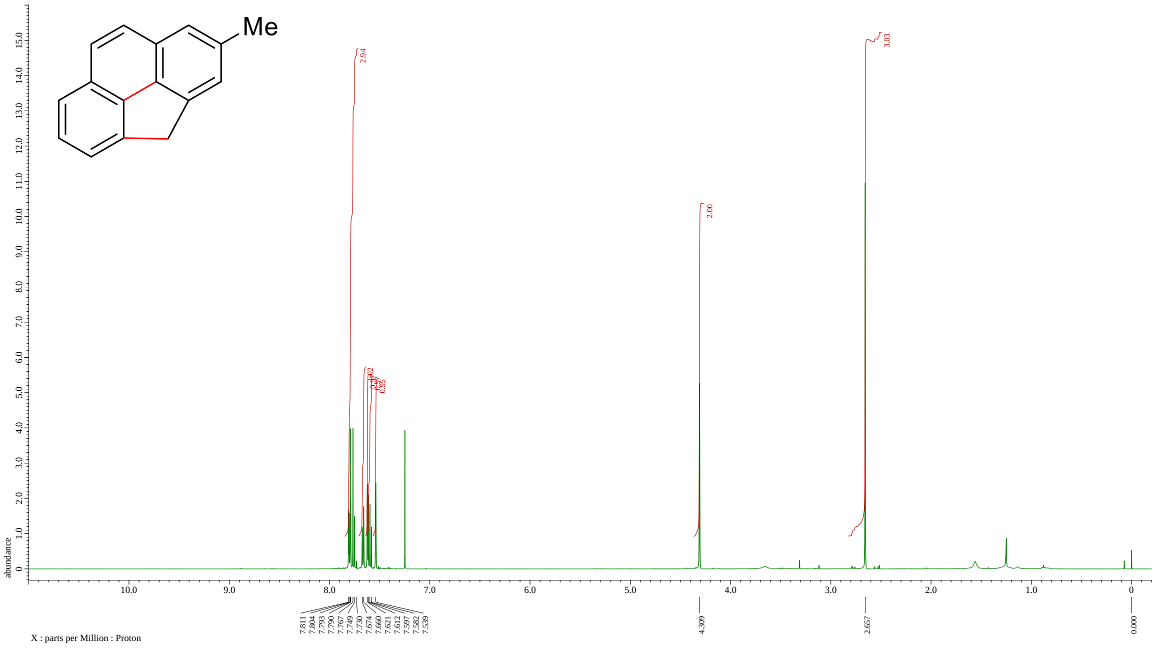

Supplementary Fig. 76 ^{1}H NMR (500 MHz, $CDCl_3$) of 2-methyl-4*H*-cyclopenta[*d,e,f*]phenanthrene (2l)

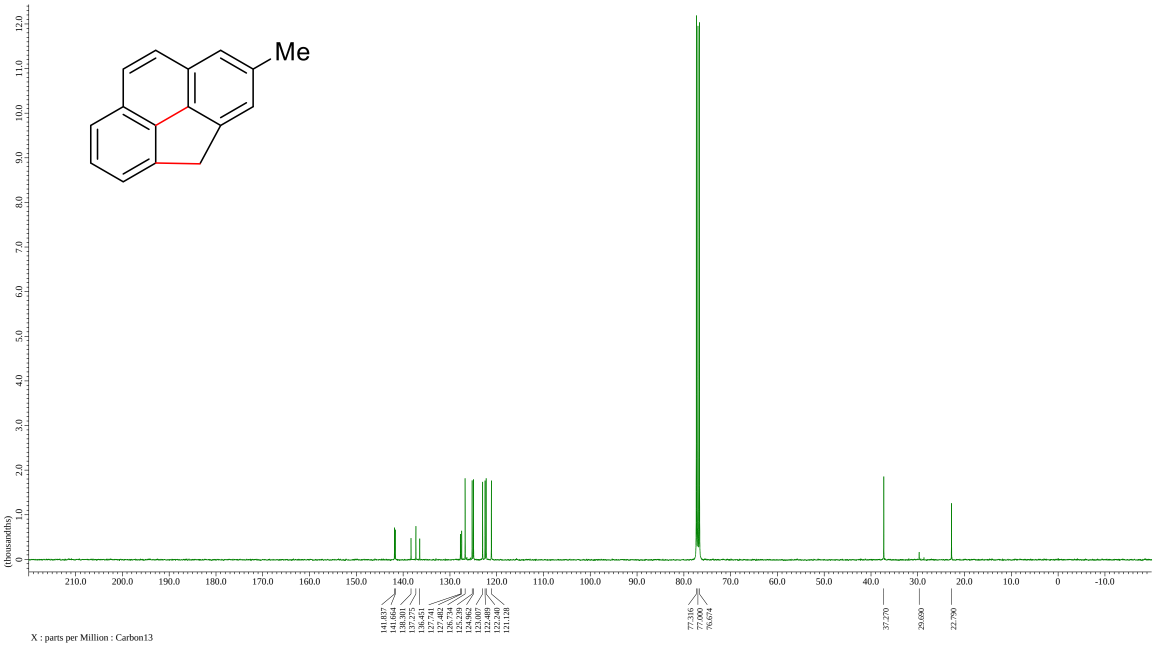

Supplementary Fig. 77 ^{13}C NMR (100 MHz, $CDCl_3$) of 2-methyl-4*H*-cyclopenta[*d,e,f*]phenanthrene (2l)

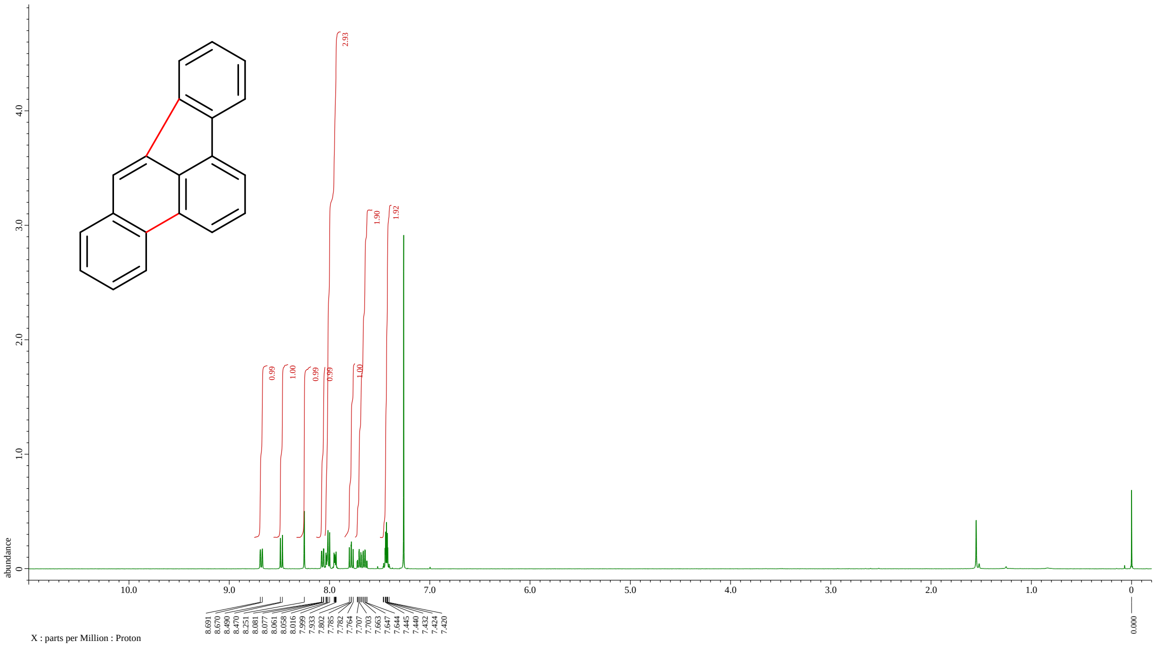

Supplementary Fig. 78 ^{1}**H NMR (400 MHz, CDCl$_3$) of benzo[*b*]fluoranthene (2m)**

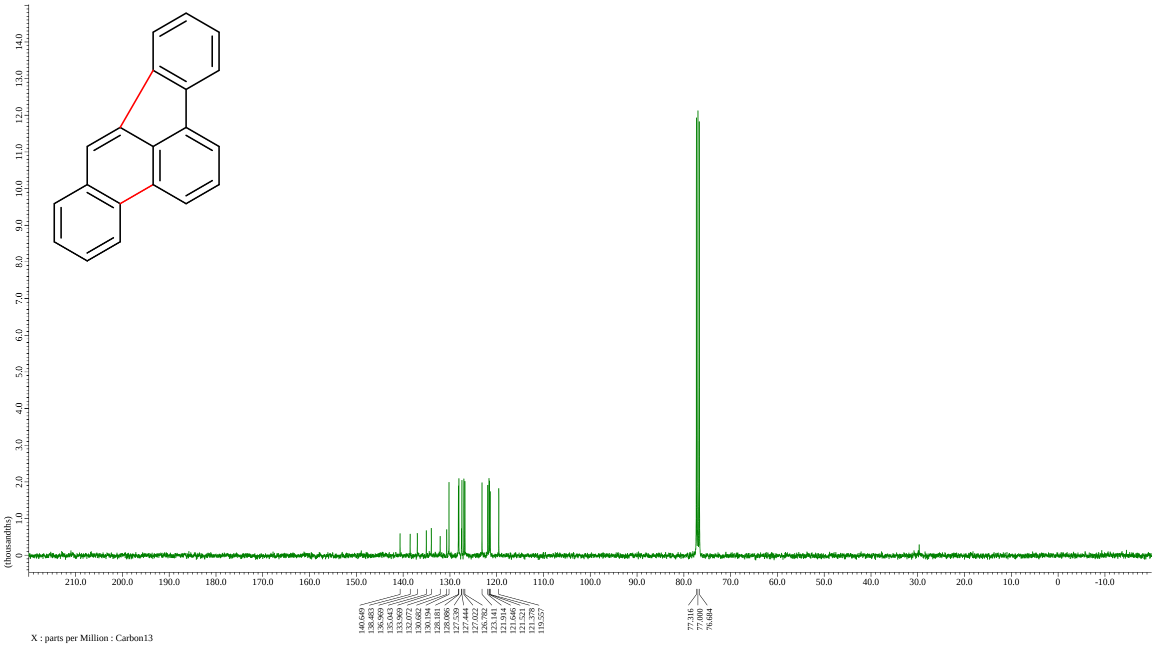

Supplementary Fig. 79 ^{13}**C NMR (100 MHz, CDCl$_3$) of benzo[*b*]fluoranthene (2m)**

Supplementary References

[1] Chen, Q., Fu, L. & Nishihara, Y. *Chem. Commun.* **56**, 7977–7980 (2020).

[2] Gallop, C. W. D., Chen, M. T. & Navarro, O. *Org. Lett.* **16**, 3724–3727 (2014).

[3] Prusty, N. et al. *Org. Lett.* **23**, 9041–9046 (2021).

[4] Krishna, J., Reddy, A. G. K. & Satyanarayana, G. *Adv. Synth. Catal.* **357**, 3597–3610 (2015).

[5] Busacca, C. A. et al. *Org. Lett.* **11**, 5594–5597 (2009).

[6] Hu, H., Yang, F. & Wu, Y. *J. Org. Chem.* **78**, 10506–10511 (2013).

[7] Liu, Y., Hu, L., Chen, H. & Du, H. *Chem. Eur. J.* **21**, 1–8 (2015).

[8] Li, X. et al. *Org. Lett.* **23**, 3304–3309 (2021).

[9] Finke, A. D., Elleby, E. C., Boyd, M. J., Weissman, H. & Moore, J. S. *J. Org. Chem.* **74**, 8897–8900 (2009).

[10] Sai, M. *Adv. Synth. Catal.* **360**, 4330–4335 (2018).

[11] Zhang, J., Lu, G., Xu, J., Sun, H. & Shen, Q. *Org. Lett.* **18**, 2860–2863 (2016).

[12] Kawamoto, T., Sato, A. & Ryu, I. *Org. Lett.* **16**, 2111–2113 (2014).

[13] Li, D. H. et al. *Organometallics* **38**, 2539–2552 (2019).

[14] Barbero, N. & Martin, R. *Org. Lett.* **14**, 796–799 (2012).

[15] Chen, M., Zhao, X., Yang, C., Wang, Y. & Xia, W. *RSC Adv.* **7**, 12022–12026 (2017).

[16] Fujita, T. et al. *Eur. J. Org. Chem.* **2017**, 262–265 (2017).

[17] Antien, K. et al. *Chem. Eur. J.* **25**, 2852–2858 (2019).

[18] Cai, X., Brown, S., Hodson, P. & Snieckus, V. *Can. J. Chem.* **82**, 195–205 (2004).

[19] Zhang, Q. et al. *J. Am. Chem. Soc.* **136**, 5057–5064 (2014).

[20] Kuninobu, Y., Tatsuzaki, T., Matsuki, T. & Takai, K. *J. Org. Chem.* **76**, 7005–7009 (2011).

[21] Chen, Y.-Z. et al. *Tetrahedron* **70**, 1748–1762 (2014).

[22] Bhojgude, S. S., Thangaraj, M., Suresh, E. & Biju, A. T. *Org. Lett.* **16**, 3576–3579 (2014).

[23] Shen, H.-C., Tang, J.-M., Chang, H.-K., Yang, C.-W. & Liu, R.-S. *J. Org. Chem.* **70**, 10113–10116 (2005).

[24] Morimoto, K. et al. *Angew. Chem. Int. Ed.* **51**, 5359–5362 (2012).

[25] Itoh, M. et al. *J. Org. Chem.* **78**, 1365–1370 (2013).

[26] Jakab, G., Tancon, C., Zhang, Z., Lippert, K. M. & Schreiner, P. R. *Org. Lett.* **14**, 1724–1727 (2012).

[27] Yamaguchi, M., Higuchi, M., Tazawa, K. & Manabe, K. *J. Org. Chem.* **81**, 3967–3974 (2016).

[28] Murai, M., Ogita, T. & Takai, K. *Chem. Commun.* **55**, 2332–2335 (2019).

[29] Shi, G., Chen, D., Jiang, H., Zhang, Y. & Zhang, Y. *Org. Lett.* **18**, 2958–2961 (2016).
